# Identification of tissue-specific, abiotic stress-responsive gene expression patterns in wine grape (*Vitis vinifera *L.) based on curation and mining of large-scale EST data sets

**DOI:** 10.1186/1471-2229-11-86

**Published:** 2011-05-18

**Authors:** Richard L Tillett, Ali Ergül, Rebecca L Albion, Karen A Schlauch, Grant R Cramer, John C Cushman

**Affiliations:** 1 Department of Biochemistry and Molecular Biology, MS330, University of Nevada, Reno, NV 89557-0330, USA; 2 Biotechnology Institute, Ankara University, Merkez Laboratuvari, Rektorluk Binasi Arkasi, 06100 Ankara, Turkey

## Abstract

**Background:**

Abiotic stresses, such as water deficit and soil salinity, result in changes in physiology, nutrient use, and vegetative growth in vines, and ultimately, yield and flavor in berries of wine grape, *Vitis vinifera *L. Large-scale expressed sequence tags (ESTs) were generated, curated, and analyzed to identify major genetic determinants responsible for stress-adaptive responses. Although roots serve as the first site of perception and/or injury for many types of abiotic stress, EST sequencing in root tissues of wine grape exposed to abiotic stresses has been extremely limited to date. To overcome this limitation, large-scale EST sequencing was conducted from root tissues exposed to multiple abiotic stresses.

**Results:**

A total of 62,236 expressed sequence tags (ESTs) were generated from leaf, berry, and root tissues from vines subjected to abiotic stresses and compared with 32,286 ESTs sequenced from 20 public cDNA libraries. Curation to correct annotation errors, clustering and assembly of the berry and leaf ESTs with currently available *V. vinifera *full-length transcripts and ESTs yielded a total of 13,278 unique sequences, with 2302 singletons and 10,976 mapped to *V. vinifera *gene models. Of these, 739 transcripts were found to have significant differential expression in stressed leaves and berries including 250 genes not described previously as being abiotic stress responsive. In a second analysis of 16,452 ESTs from a normalized root cDNA library derived from roots exposed to multiple, short-term, abiotic stresses, 135 genes with root-enriched expression patterns were identified on the basis of their relative EST abundance in roots relative to other tissues.

**Conclusions:**

The large-scale analysis of relative EST frequency counts among a diverse collection of 23 different cDNA libraries from leaf, berry, and root tissues of wine grape exposed to a variety of abiotic stress conditions revealed distinct, tissue-specific expression patterns, previously unrecognized stress-induced genes, and many novel genes with root-enriched mRNA expression for improving our understanding of root biology and manipulation of rootstock traits in wine grape. mRNA abundance estimates based on EST library-enriched expression patterns showed only modest correlations between microarray and quantitative, real-time reverse transcription-polymerase chain reaction (qRT-PCR) methods highlighting the need for deep-sequencing expression profiling methods.

## Background

The study of gene function in the wine grape (*Vitis vinifera *L.) has been fundamentally advanced by the availability of whole genome sequences of two Pinot Noir cultivars (clones 115 and PN40024) [1,2] as well as BAC-based physical maps [3]. To study wine grape gene function, multiple transcriptomic approaches have been developed [4,5], including expressed sequence tags (ESTs) [6], massively parallel signature sequencing (MPSS) [7], small RNA deep sequencing [8], Illumina sequencing [9], and multiple oligonucleotide microarray platforms [10-13].

Most *V. vinifera *varieties are ranked as moderately sensitive to sensitive to salinity stress [14-17] with Cl^- ^anion toxicity having the greatest impact on growth and vine health [18]. In contrast, *V. vinifera *is relatively water-deficit stress tolerant. Regulated-deficit irrigation can be used advantageously to inhibit vine growth without significant effects on fruit yield and has been reported to improve grape quality through the elevation of a variety of metabolites including anthocyanins and proanthocyanins [19-22]. mRNA and enzyme expression profiles during development and in response to abiotic stress effects have been studied intensively in wine grape berries [11,12,23-30]. Additional studies have examined mRNA expression patterns in response to abiotic stresses in leaves and shoot tissues [10,31], plant-pathogen interactions [13,32,33], and the events associated with *Vitis *bud endodormancy [34-36].

The roots of terrestrial plants are vital organs for the acquisition of water and essential minerals. As such, roots serve as the first site of perception and/or injury for many types of abiotic stress, including water deficiency, salinity, nutrient deficiency, and heavy metals [37-39]. *Vitis *roots also accumulate a number of unique stilbene and oligostilbene defense compounds, chemical species not found in seed or other phytoalexin-rich tissues [40,41]. Despite the importance of roots, the study of *V. vinifera *root tissues has been rather limited in contrast to the study of berry tissues. In a comparative EST study, Moser and colleagues generated 1555 ESTs from *V. vinifera *cv. Pinot Noir root tissue and found them enriched for genes with functions in primary metabolism and energy [42]. Using a 12 K CombiMatrix custom array, Mica and colleagues profiled the expression of microRNAs (miRNAs), small (19-24 nt) non-coding RNAs that negatively regulate gene expression post-transcriptionally in multiple organs. This study showed that roots had nine and four miRNAs with either significantly increased or decreased relative abundance, respectively, relative to leaves and early inflorescences [8]. A framework physical or genetic map has also been developed for wine grape, using resistant and susceptible crosses, to locate genetic determinants associated with resistance to the root pathogen phylloxera [43]. EST transcriptional profiling has recently been used to identify genes that might be involved in resistance to *Rhizobium vitis *in the semi-resistant *Vitis *hybrid 'Tamnara' [44].

In grapevine, more than 350,000 EST sequences have been generated and analyzed to identify gene expression related to a wide range of processes including berry development in wine grape [30,45] and in table grape [46], tissue-specific gene expression [6,42], the fulfillment of chilling requirements in dormant grape buds [34], and the characterization of resistance to pathogens such as *Xylella fastidiosa *[47] and *Rhizobium vitis *[44]. To discern how steady-state transcript accumulation changes in response to multiple environmental stress treatments, we generated a total of 45,784 ESTs from leaf and berry tissues from vines subjected to abiotic stresses (e.g., salinity, cold, heat, water deficit, and anoxia). These were compared with 32,286 ESTs within 20 libraries derived from leaf and berry tissues deposited in the public databases. Clustering and assembly of leaf and berry ESTs with all available *V. vinifera *full-length transcripts and ESTs returned a total of 13,278 unique sequences, with 2302 singletons and 10,976 clusters mapping to known gene models. Of these 10,976 unique clusters, 739 transcripts were found to have significant differential expression among the libraries examined. Comparison of *in silico *digital expression analysis with transcript abundance estimates obtained by Affymetrix *Vitis *GeneChip^® ^genome microarrays and quantitative real-time reverse transcription-polymerase chain reaction (qRT-PCR) revealed that EST frequency counts were in moderate agreement with microarray or qRT-PCR analysis. Given the relative lack of ESTs available for grape root tissues, 16,452 ESTs were sequenced from roots of young vines (10 cm in length), grown under unstressed conditions as well as under cold, salinity, and water deficit stress. The major categories of genes expressed in root tissues were defined and 135 genes with root-specific or highly enriched root expression patterns were identified.

## Results

### EST library analysis from abiotically stressed tissues of *Vitis vinifera*

cDNA libraries derived from abiotically stressed leaves (Library ID 10208) and berries (Library ID 12435) of *V. vinifera *cv. Chardonnay, were sequenced to generate 24,400 and 21,384 ESTs, respectively (Table 1). In addition, a total of 16,452 ESTs were sequenced from a normalized cDNA library synthesized from Magenta box grown root tissues from cv. Cabernet Sauvignon exposed to control, water deficit, cold, and salinity stress conditions (see Methods section for details) (Library ID 22274). In total, 66,236 expressed sequence tags (ESTs) were generated (Table 1). The leaf and berry libraries were described previously in the context of flower and berry development [6]. In addition, five unstressed leaf libraries, representing a total of 8642 ESTs, 13 whole berry with seeds libraries derived from unstressed source tissues at various stages of berry development, representing a total of 31,840 ESTs, and two root libraries, representing a total of 1657 ESTs, present within the UniGene database [48] were compiled (Table 1). These EST collections were used as tools to identify transcripts encoding abiotic stress responsive transcripts in leaves and berries and root-specific or root-enriched transcripts.

**Table 1:** cDNA Library Attributes

Tissue	dbEST Library ID	cDNA Orientation (5'/3')	Submitted Description	Developmental Stage	ESTs as per dbEST	Unique clones
Stressed Leaf	10208	both	An expressed sequence tag database for abiotic stressed leaves of *Vitis vinifera *cv. Chardonnay	Juvenile & adult	24,400	21,499
Stressed Berry	12435	both	An expressed sequence tag database for abiotic stressed berries of *Vitis vinifera *cv. Chardonnay	Mixed: 8, 9, 11, 13, 15, 16 weeks DAF	21,384	18,963

Leaf	12752	both	Cabernet Sauvignon Leaf - CA32EN	Mid-season	2,669	1,465
Leaf	12753	both	Cabernet Sauvignon Leaf-CA48EN	Mid-season	2,051	1,104
Leaf	12948	both	Cabernet Sauvignon Leaf - CA48LN	Late Season	2,248	1,441
Leaf	12949	both	Cabernet Sauvignon Leaf - CA41LN	Late Season	1,146	739
Leaf	14446	5'	Grape Leaf pBluescript Library	Juvenile	528	528

				*Leaf subtotal*	*8642*	*5277*

Berry	4059	both	Grape berries Lambda Zap II Library	Véraison	105	96
Berry	8669	3'	Green Grape berries Lambda Zap II Library	Pre-véraison	1,989	1,989
Berry	8670	3'	Ripening Grape berries Lambda Zap II Library	Post-véraison	3,268	3,267
Berry	8671	both	Véraison Grape berries Lambda Zap II Library	Véraison	96	96
Berry	11063	3'	Véraison Grape berries SuperScriptTM Plasmid Library	Véraison	623	623
Berry	11064	3'	Véraison Grape berries Lambda Zap II Library	Véraison	1,691	1,691
Berry	12754	both	Cabernet Sauvignon Berry - CAB2SG	Pre-véraison	4,429	2,339
Berry	13015	both	Cabernet Sauvignon Berry Stage I - CAB3	Pre-véraison	3,414	1,955
Berry	13016	both	Cabernet Sauvignon Berry - CAB4	Pre-véraison	3,836	2,155
Berry	13017	both	Cabernet Sauvignon Berry Post-Véraison - CAB7	Post-véraison	3,558	1,911
Berry	14444	5'	Grape Berry pSPORT1 Library	Véraison	1,743	1,743
Berry	20043	n.d.	Clusters 4 cm (VvC3)	Pre-véraison	4,053	4,053
Berry	20044	n.d.	Berries Véraison stage (VvC4)	Véraison	3,035	3,035

				*Berry subtotal*	*31840*	*24953*

Root	14445	5'	Grape Root pSPORT1 Library	One year-old root	1,555	1,555
Root	16696	n.d.	*Vitis vinifera *Cabernet Sauvignon root	n.d.	102	102
Stressed Root	22274	5'	VVM - Normalized Cabernet Sauvignon root	Young vines	16,452	16,452

**Total**					**104,402**	**88,828**

Libraries generated or used in the present study. The Stressed Leaf (SL) and Stressed Berry (SB) libraries were generated previously [6], the Stressed Root "VVM" library was generated specifically for this study, and all other libraries were obtained from the dbEST database maintained by the NCBI. Tissues, dbEST library identifier, sequencing direction, and library descriptions are provided. Unique clones were identified from the ESTs of bidirectionally sequenced libraries as described in the "Methods" and "Results" sections.

n.d. not determined

To create up-to-date annotations, each EST was matched with the corresponding "tentative consensus" (TC) contig sequence from the *Vitis vinifera *Gene Index (*Vv*GI, version 6, July 30, 2008, Dana Farber Cancer Institute) [49] and predicted peptide sequences from the Genoscope 8.4X *Vitis vinifera *cv. Pinot Noir (GSVIV) genome assembly, August 8, 2007 [1]. A newer version of VVGI (7.0, 4/17/2010) was released since this analysis was undertaken. However, this release is substantially similar to 6.0, containing the same 25,497 gene models derived from the NCBI RefSeq source and only 4851 additional ESTs and was not expected to substantially alter the findings presented. A newer 12X coverage draft of the *Vitis vinifera *genome has also become available. However, some gene models annotated in this 12X draft were found to contain greater frequencies of intron-exon splices not supported by EST evidence (data not shown) and, therefore, the 12X draft was not used. Because the mixed stress normalized root library was generated using a normalization technique that would, in effect, reduce the apparent expression of the most abundant transcripts, and because few other unstressed root ESTs were available for comparison, characterization of the genes in the root EST library was performed in a separate analysis.

#### Identifying EST redundancy

In estimation of gene expression patterns inferred from EST frequencies, which are the number of times the transcript of gene x_i _is observed in relation to the total number of random observations of all genes, (x_i _/ ∑x), any ESTs from a single clone sequenced from both the 5' and 3' directions must be counted exactly once to avoid overestimation of the frequency of genes. cDNA library sequencing strategies varied among sources, with some ESTs being generated from only single-pass 5' or 3' reads, whereas other libraries were subjected to bi-directional and/or same-direction re-sequencing of picked clones. In the abiotically stressed leaf library (Library ID 10208), 2802 ESTs had been sequenced twice (representing 1401 paired reads). Another 2250 ESTs had been sequenced three times (750 triplets). Eliminating this redundancy reduced the EST total from 24,400 ESTs to 21,499 unique clones (Table 1). The GSVIV gene identifiers of paired clone ends were compared with the expectation that gene IDs would agree between multiple ESTs from the same transcript. Of the 1401 pairs of clones from this abiotically stressed leaf library, 114 (7%) pairs matched the annotation of different genes and the ID with greatest confidence score was retained. Similarly, only 60 of the 750 triplicated clones (8%) within this library were in disagreement as to gene identity. The total clone redundancy and gene assignment error rates were similar for ESTs from the stressed berry library (Library ID 12435). In this EST collection, 2402 ESTs had been sequenced twice (representing 1201 pairs), 1821 ESTs had been sequenced three times (607 triplets), and two clones had been sequenced four times each. Eliminating this redundancy reduced the EST total from 21,384 ESTs to 18,963 unique clones (Table 1). Of the 1201 pairs and 607 triplicates, 107 (9%) and 68 (11%) were in disagreement, respectively.

**Figure 1:**
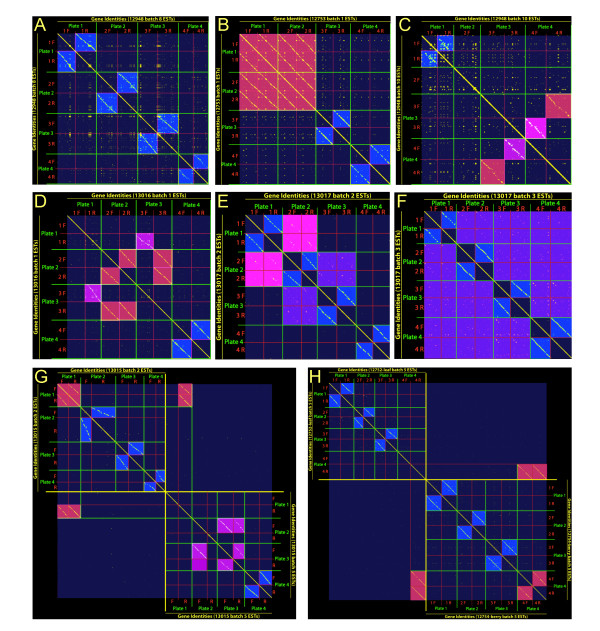
**Correcting erroneous EST identities in bi-directionally sequenced leaf and berry libraries with dot plots**. Contig names assigned to ESTs from bi-directionally sequenced libraries were plotted in two dimensions to identify "motifs of self-similarity" analogous to dot-plot sequence alignments. The sequencing batch, plate order, and well position were recapitulated from dbEST submission files as a sequential list arranged as *1f, 1r, 2f, 2r, 3f, 3r, 4f, 4r*, and plotted against itself in the x and y axes. **A) **Diagonals indicate four sets of plates from Library ID 12948, batch 8 are named and paired correctly (blue); **B) **Library ID 12753, batch 1, all combinations of plates *1f, 1r, 2f *and *2r *are duplicates (salmon), plates 3 and 4 are correctly paired (blue); **C) **Library ID 12948, batch 10 plate 1f matches 1r (blue), plate 2f and 2r did not match, plate 3f matches 4r (salmon), 4f matches 3r (magenta); **D) **Berry Library ID 13016, batch 1, plate 3r matches with 2f and 2r (salmon), 1r matches with 3f (magenta), 1f has no match, plate 4 is paired correctly (blue); **E) **Library ID 13017, batch 2, Plates 1 and 2 display partial matching (pink), plates 2 and 3 also partially match (purple); **F) **Berry Library ID 13017 batch 3, partial matching between all four plates (purple); **G) **Berry Library ID 13015, batch 2, plate 1 matches batch 5 plate 1r (salmon); other plate match errors are also apparent in lower right hand quandrant (magenta); **H)**, Leaf Library ID 12752, batch 5, plate 4r matches Berry Library ID 12754, batch 5, plates 4fr (salmon).

This method of redundancy elimination was extended next to those bi-directionally sequenced clones from the non-stressed leaf and berry libraries obtained from the UniGene database (Table 1). Many errors were found in the annotated compositions of leaf (Library IDs: 12752, 12753, 12948, and 12949) and berry libraries (Library IDs: 12754, 13015, 13016 and 13017). The errors and the corrections made are explained below as presented in Figure 1 and summarized in Table 2. For the Cabernet Sauvignon leaf library CA48LN (Library ID 12948), we were able to organize 1486 ESTs into 743-paired reads. Within these pairs, > 68% (509) could not be assigned to the same gene. Similarly, high rates of disagreement were found within other libraries listed in Table 2. As these rates were higher than those observed in paired reads from abiotically stressed leaf or berry libraries, the cause or causes of these higher error rates were investigated further.

**Table 2:** Correction of errors in the identifications of ESTs in a set of libraries

Error category	Specific error type	# of Errors	See also
*Well pairing "slips"*			
**Leaf **Library ID 12948		10	Figure 1A
**Leaf **Library ID 12949		1	
*Incorrect plate pairings*			
**Leaf **Library ID 12753	Plate quadruplicated	1	Figure 1B
**Berry **Library ID 12754	Plate quadruplicated	1	
**Leaf **Library ID 12948	Plate pair swap	2	Figure 1C
**Berry **Library ID 13015	Plate pair swap	2	
	Plate triplicated	3	Figure 1G
**Berry **Library ID 13016	Plate triplicated	1	Figure 1D
	Plate pair swap	1	
*Partial plate duplications*			
**Berry **Library ID 13016		6	
**Berry **Library ID 13017		28	Figure 1E, 1F
*Sequences originated from a different library*			
**Leaf **Library ID 12752 and **Berry **Library ID 12754	Plate of "leaf" ESTs actually a triplicate of berry Lib.12754 ESTs	1	Figure 1H

Errors in the supplied annotation of a set of cDNA clones that were sequenced bidirectionally were identified and corrected to generate accurate counts of EST frequency. Errors are categorized by the scope of the error, from "well slips" between single pairs of 5' and 3' 96-well plates of ESTs, through incorrectly identified pairs of plates of increasing scope. The number of times each error occurred (pairs or larger groups of 96-well plates affected) and was corrected is shown. Errors that are visualized by dot-plot in Figure 1 are cross-referenced.

The cDNA libraries presented in Table 2 were bidirectionally sequenced and had annotation that allowed for the partial reconstruction of the workflow by which they were prepared and sequenced originally [50] with clone names deposited to NCBI such as "CA48LN09IF-A9, 5'end." This annotation identifies the library "CA48LN," a batch number ("09," the plate within that batch (I), location on a 96-well plate (A9) and direction (5'). All ESTs in a given library shared the library stem, batches generally contained four plates (I-IV), and 80% of plates were sequenced from both 5' and 3' directions. When the forward and reverse pairs of ESTs in Library ID 12948 were organized by their 96-well plate well order (A1,A2,...,A12,...,H1,...,H12), various patterns of "well slip" were identified, wherein the gene ID for well A9 (5') matched the gene ID of well A10 (3'), A10 (5') matched A11 (3'), and so forth. The distance of these "well slips" was neither uniform nor consistent.

To determine all pairs of ESTs with incorrectly paired wells, a method was devised that would identify robustly "well slips" of non-uniform distances, analogous to the dot-plot method of local nucleotide sequence alignment [51]. In this method, the gene IDs of ESTs were arranged from A1-H12 for each 5' and 3' plate and plotted along two axes with a dot designating wherever the gene IDs were identical (Figure 1). The dot plot proved effective at identifying forward-reverse pairing in plates with "well slips," such as in Figure 1A, wherein the four forward and reverse plates of "batch 09" in leaf Library ID 12948 were plotted in the order 1f, 1r, 2f, 2r, 3f, 3r, 4r along both the × and y axes. The main diagonal bisecting the plot, where the ordered list is identical to itself, is flanked by four offset diagonals that illustrate where the forward and reverse plate pairs match (1f≈1r, 2f≈2r, *etc*.). The matching clearly distinguished pairs of plates through the variable "well slips" in Library ID 12948.

This matching process was repeated for all plate batches (generally four forward and four reverse plates per batch) of the libraries listed in Table 2 and other error types besides the "well slips" seen in Library ID 12948 were uncovered. Some plates were duplicated, as seen in Figure 1B, wherein all combinations of the forward and reverse of four individual plates matched in berry Library ID 12753 (1f≈1r≈2f≈2r). Were these errors not identified, the ESTs of plate 1 and 2 would have been added both to the frequency totals of the genes therein (i.e., counting twice what should only be counted once), resulting in an overestimation of the frequency of those transcripts in the library. Other pairs of plates showed a less complete duplication pattern as seen as the inchoate diagonals between plates 1 and 2 (pink) and between 2 and 3 (purple) in Figure 1E, and all four plates (purple) in Figure 1F. In other cases, a plate did not match the annotated reverse, but a different plate instead, such as the pair-swapping of Library ID 12948 (3f≈4r and 4f≈3r) in Figure 1C and triplication (2f≈2r≈3r) / mis-pairing (3f≈1r) in berry Library ID 13016 (Figure 1D). Where identified, these partial duplications and mismatched plates were handled just as the full duplications were, with the EST counts reduced to reflect the true number of independent clones involved.

The same analytical method was then extended to compare every plate in a library to all other plates in that library. One additional case of unexpected matching was found, where plates from one batch match the plates of a different batch in the same library (Figure 1G). Lastly, we extended the method to compare every plate in every library against all other plates in all other libraries, even those annotated as arising from different tissues. From this, a single instance was found where a plate in the leaf Library ID 12752 (plate 4r) was identical to a pair of plates from berry Library ID 12754 (Figure 1H). The genes encoded on these plates were consistent with those found in mature berry library (e.g., cell wall proteins, ripening-related proteins, and no photosynthesis genes), but not a leaf library, leading to the conclusion that a cDNA library misassignment error had occurred, and leading to the exclusion of these data from our analyses. To uncover other possible library assignment errors, every plate from all libraries in the present study were compared against all other libraries (e.g., bud tissues, petioles, flowers, and pathogen infected leaves) that were not considered for our abiotic stress analysis, but no further spurious pairings were detected (data not shown). Upon exhaustively identifying all observable patterns of errors, 5' ESTs were paired with their 3' partners and the unique clones within each library were counted (Table 1). In total, errors in the identification/annotation of 5558 of 23,351 ESTs (24%) were discovered from the libraries listed in Table 2.

#### Estimating gene expression by EST frequency

**Figure 2:**
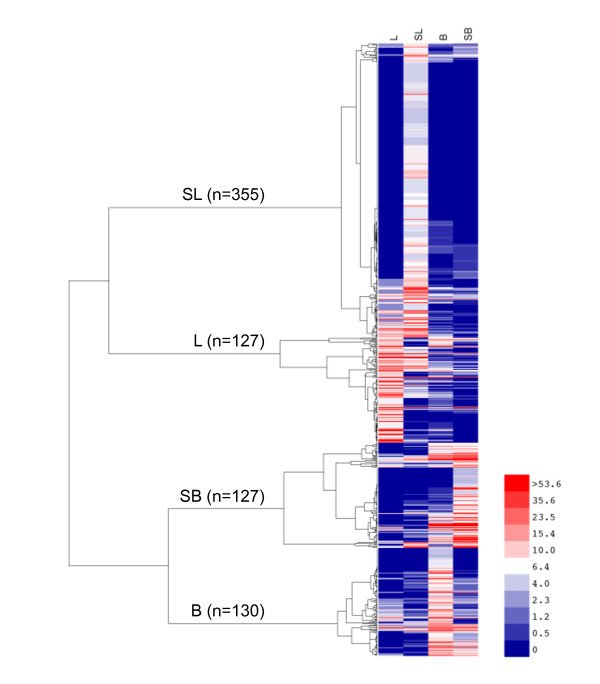
**Heat-map and two-dimensional hierarchical clustering of EST frequencies in 739 differentially expressed genes among cDNA libraries from stressed and unstressed leaves and berries**. Color shown is given in normalized EST frequency per 10,000 ESTs, scale from blue at *f *= 0 to white to red at *f *> 53.6 (inset). Four major clusters that correspond to single-type predominance are labeled (with the number of genes within the cluster) stressed leaf **(SL)**, leaf **(L)**, Stressed Berry **(SB)**, Berry **(B)**.

In order to measure differences in gene expression patterns among stressed and unstressed leaves and berries, the EST frequency within each GSVIV gene ID (or UniGene ID, in cases where no GSVIV gene model could be assigned) was calculated for each leaf, berry, stressed leaf, and stressed berry library. The EST frequencies of the five leaf libraries were combined by weighted mean, as were the 13 berry frequencies [52]. Differential gene expression was then calculated using the combined EST frequency counts for genes using the IDEG6 web tool [53]. The chi-squared test (χ^2^) was used as the test statistic, as recommended when conducting statistical comparisons of more than two groups [54]. At a p-value cutoff of < 0.001, 739 genes were estimated to have differential expression among the libraries compared. The 739 genes were then organized by hierarchical clustering, using a function of the Pearson correlation coefficient as the distance metric and the average agglomeration method (Figure 2). The sets of genes clustered first between tissue type, as seen by the first branching in the dendrogram, and then by control or abiotic stress condition, as seen in the next two branches. At this distance the four clusters generally correlated to transcript abundance profiles within a single library type with the largest cluster of 355 transcripts corresponding to tissues of stressed leaves (SL). The leaf cluster (L) contained 127 genes, whereas stressed berry (SB) and unstressed berry clusters (B) contained 127 and 130 genes, respectively. The annotation, gene models, and relative frequencies of all 739 genes are listed by cluster in Additional Files 1, 2, 3 and 4. The high number of transcripts present within the stressed leaf cluster might reflect the depth to which this library was sequenced, the variety of abiotic stresses to which these source tissues were subjected, and the diversity of transcripts expressed within the grape leaf transcriptome under abiotic stresses [10].

**Figure 3:**
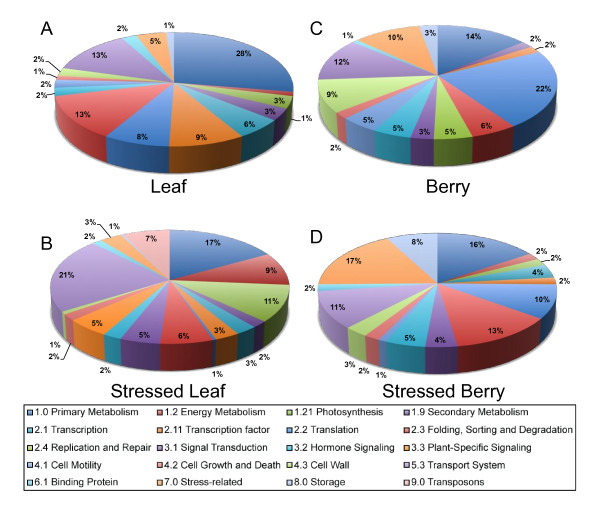
**Functional categories of differentially expressed transcripts identified by EST frequency analysis**. Functional assignments of genes found in the four major clusters of differentially expressed genes. At the chosen hierarchy depth / distance, the four clusters correspond, in large part, to maximal frequencies within **A**) Leaf, **B**) Stressed Leaf, **C**) Berry, and **D**) Stressed Berry cDNA libraries. Assignments are based upon the data available at *Vitis*Net http://www.sdstate.edu/aes/vitis/pathways.cfm[93]. Chart colors progress clockwise from the top.

Of these 739 genes with differential expression among the cDNA library clusters, 637 were matched successfully to GSVIV gene/protein identifiers, which were then matched with the annotation files associated with *Vitis*Net [55]. *Vitis*Net networks were combined into categories of their major networks, with metabolic networks divided into primary metabolism, photosynthesis, secondary metabolism, and hormone biosynthesis, the latter category being grouped with the hormone signaling category. Gene IDs that were "out-of-network", but that had functional annotations associated with them in the *Vitis*Net master list were also incorporated into the functional category designations. In Figure 3, the functional categories of genes identified within the four major clusters are shown.

Without over-interpretation, some key differences among the functional categories of genes prominent within each organ/condition are clearly apparent. For example, unstressed leaves (Figure 3A) were distinguished by a large proportion (28%) of primary metabolic genes with some photosynthetic genes, such as RUBISCO small subunit and plastidic photosynthetic electron transport components being extremely over represented. Transcripts for non-specific lipid-transfer protein, metallothionein, early light-induced protein (ELIP1), and several unknown genes were also highly represented within this cluster along with 23S rRNA (Additional File 2). In stressed leaf, 11% of transcripts encoded photosynthesis-related functions, including plastidic ATP synthase and electron transport chain subunits, suggesting that higher demands and/or damage might occur under stress that must be repaired (Figure 3B). Consistent with this suggestion is the over representation of several families of low molecular heat shock proteins. Leaves under abiotic stress expressed a greater proportion of specific transport genes (21%) (Additional File 1). Interestingly, the activity of transposons is apparently de-repressed in stressed leaves as judged by the preponderance (7%) of a centromere-specific class of retrotransposons. Similar abiotic induction of retroelements in non-germline tissue has been described in Solanaceous species and the ABA-induction of the Tnt1A promoter in *Arabidopsis thaliana *[56]. The unstressed berry cluster possessed overrepresented transcripts encoding genes with functions involved in primary metabolism, translation, cell wall-related proteins (9%), and transport (12%) (Figure 3C, Additional File 4). In contrast, the stressed berry cluster (Figure 3D) had the highest proportion of genes annotated as "stress-responsive" (17%) including overrepresented transcripts encoding xyloglucan endotransglucosylase/hydrolases, a DEAD box RNA helicase, and seed storage proteins including albumins and globulins and several highly abundant unknown proteins (Additional File 3).

#### Correlation with microarray data

**Figure 4:**
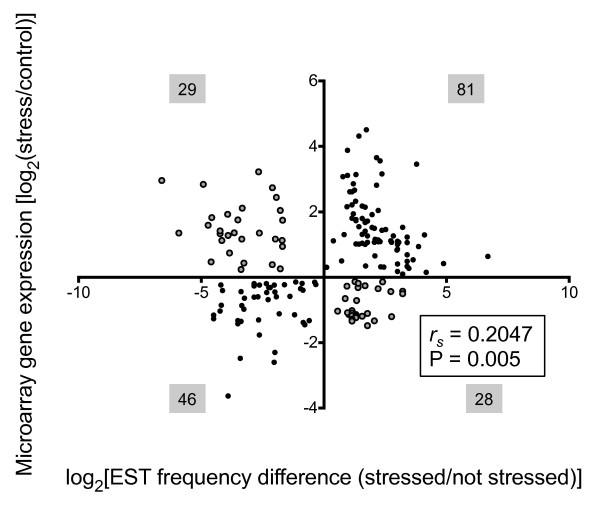
**Scatterplot of EST frequencies compared with microarray expression levels**. Log_2_-transformed frequency distributions of ESTs from mixed stressed leaf (e.g., water deficit, NaCl, heat, high light) and berry (water deficit stress) and unstressed leaf and berry tissue were compared to 184 Affymetrix^® ^*Vitis *GeneChip^® ^log_2_-abundance ratios of chilling, osmotic (mannitol), and salt stress, and water-deficit-stressed leaf [10,31] and water-deficit-stressed whole berry tissues [24]. Differences in gene model EST frequencies between stressed and unstressed library pairs (i.e., stressed berries compared with unstressed berries) were plotted along a log_2 _scale as well. The Spearman rank correlation, *r_s_*, was 0.2047, with likelihood P = 0.005). Filled and gray circles indicate agreement and disagreement in directional concordance, respectively. The total number of genes present in each Cartesian quadrant are shown in gray-shaded boxes.

Next, differences in transcript expression patterns estimated by EST frequency were compared with a second platform, the Affymetrix^® ^*Vitis *GeneChip^® ^microarray. Of the 739 transcripts described above, microarray probeset identifiers could be assigned for 489 of them. All differentially expressed genes available from microarray experiments in which similar stresses were imposed were collected. For leaf tissue, within which our stressed leaf library included a mixture of drought, NaCl, heat, and light stressed tissue, two experiments were used as a source for microarray data: an experiment in which drought and salt stress were applied over a 16 d period [10] and an experiment that analyzed rapid changes (≤ 24 h) in gene expression under osmotic stress (mannitol), NaCl, and chilling exposure [31]. For the berry libraries, microarray data from a drought stressed berry time course experiment of Chardonnay and Cabernet Sauvignon [27] were compared with EST frequency data. Following the example of van Ruissen and colleagues [57], probeset expression values were then compared with EST frequencies using only those probesets for which significant differences were observed between stressed and unstressed tissues in the original microarray experiments. Using this method, 184 comparisons of significantly different changes were plotted (Figure 4). Overall correlation between the microarray and frequency-based expression measures was modest. The non-parametric Spearman rank correlation was modestly positive, at (*r_s _*= 0.2), but with a P < 0.005, indicating that this similarity, while modest, is extremely unlikely to be due to chance alone. Pearson correlation was similar (*r *= 0.21). In other studies comparing microarray to EST or similar tag-based technologies, modest Spearman and Pearson correlations have been observed [58]. Following the example of Li and colleagues, the directional concordance, which is the directional agreement in either increased or decreased relative transcript abundance in response to stress treatment, among the 184 significant genes common to both microarray or EST sampling detection methods was determined. In their comparison of SAGE tags with microarrays in multiple human tissues, these authors found 75% directional concordance among significant genes [58]. Similarly, for our 184 shared genes, the directional concordance was 69% or more than two agreements per disagreement.

**Figure 5:**
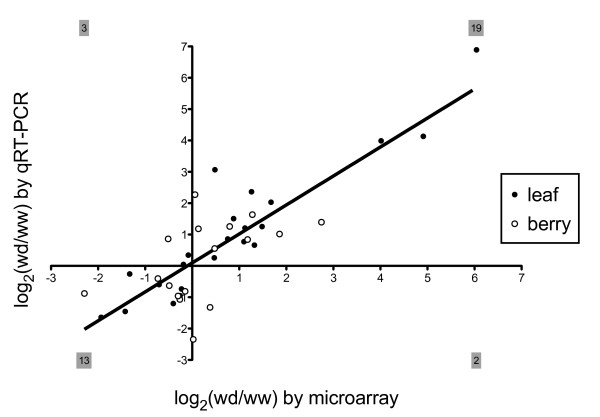
**Expression of stress-related genes in *V. vinifera *leaves and berries as detected by microarray and qRT-PCR**. Log_2_-transformed values of Affymetrix^® ^*Vitis *GeneChip^® ^signal intensities (x-axis) and real time-RT-PCR expression log_2_-ratios (y-axis) of 22 genes in leaf tissue (filled circles), as well as 17 genes in berry tissue (open circles) of water deficit (wd) and well watered (ww) vines. A linear regression has slope *m *= 0.92 and Pearson correlation *r *= 0.85 for the total data set of 39 pairs of log_2_-ratios [10,24]. The totals of genes present in each Cartesian quadrant are shown in gray-shaded boxes. qRT-PCR data were derived from three biological replicates.

In order to verify the gene expression ratios determined by microarray analysis, qRT-PCR was performed on the set of genes listed in Additional file 5. These genes were selected at random and represented genes expressed preferentially in either leaf or berry tissues. Relative mRNA expression for 17 and 22 transcripts was assayed in drought-stressed and well-watered berry tissue and leaf tissue, respectively. A linear regression of the log_2_-ratios of those genes found strong correlation between transcript abundance measured by microarray and qRT-PCR methods (Pearson correlation, *r *= 0.85) and a very high degree of directional concordance (34/39 genes or 87%) (Figure 5).

### Identification of root-enriched genes

**Figure 6:**
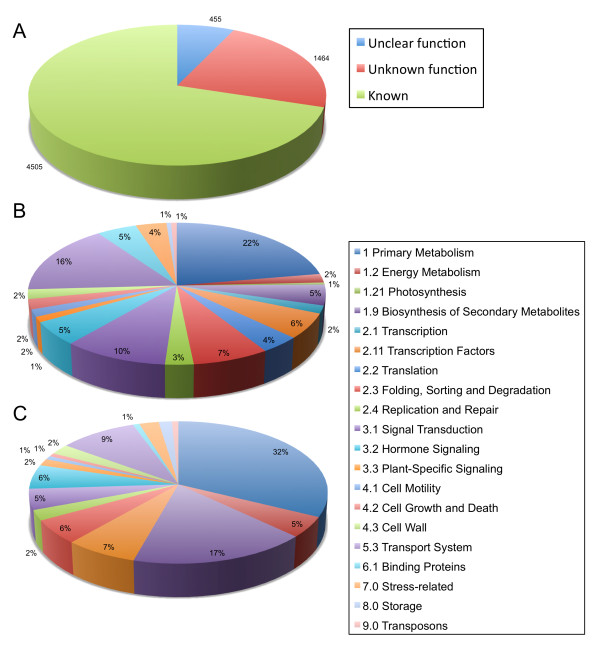
**Functional categories of genes in the VVM root cDNA library and within a root-enriched subset**. Functional assignments for genes from the Cabernet Sauvignon root EST library, VVM, were made using *Vitis*Net annotation. A) Proportion of genes identified in VVM for which functions are unclear, unknown, or are known as described within *Vitis*Net annotation; B) Classification of the functions of all 4505 genes from the above "known" category in VVM; C) the functional assignments of 135 transcripts estimated to be differentially expressed in root tissues from the Audic-Claverie test [60].

The 16,452 ESTs sequenced from the normalized abiotic stressed Cabernet Sauvignon root cDNA library (VVM) were matched to their *Vv*GI ver. 6 consensus sequence contigs [59] and, when possible, to the 8.4X genomic GSVIV gene/protein identifiers and matched with the annotation files associated with *Vitis*Net [55], resulting in the identification of 6424 non-redundant transcripts. Of these, 6002 were mapped successfully to 8.4X GSVIV gene models, whereas the remaining 307 singletons and 115 *Vv*GI contigs did not match GSVIV gene models. The cDNA library normalization method was successful in generating a highly complex library, with 3449 (54%) unique transcripts being represented by EST singletons. Annotation of the 6424 non-redundant root transcripts revealed 4505 (70%) had known functions, 455 (7%) matched a previously annotated gene model, but the function was unclear, and 1464 (22.8%) had unknown functions, with no homology matches to any previously described gene (Figure 6A). The functional categories were assigned for the 4505 transcripts with known functions (Figure 6B). Overall, the VVM normalized library contained a high diversity of transcripts with the functional categories of primary metabolism, signal transduction, and transport systems being well represented (Figure 6B).

Next, the 16,452 VVM Cabernet Sauvignon root ESTs plus an additional 1657 ESTs from two Cabernet Sauvignon root libraries (Library ID 14445, 16696; Table 1) were analyzed for either root-specific or root-enriched transcripts. These root cDNA libraries were compared with a total of 291,233 ESTs from 114 libraries comprising the NCBI UniGene dataset http://www.ncbi.nlm.nih.gov/UniGene/lbrowse2.cgi?TAXID=29760[48] with the exception of five EST libraries derived from *in vitro *or cell cultures (Library ID 10498, 15513), mixed organ (e.g., root and leaf together) cDNA libraries (Library ID 20007, 20010), or an amplified fragment length polymorphism (AFLP) cDNA library (Library ID 20099). Relative EST frequency counts were calculated as previously described using weighted averages for the combination of libraries grouped into either "root" or "non-root" groups. EST frequency counts for genes with two or more ESTs within one or both of the library sets (singletons were removed) and corresponding differential gene expression patterns were calculated with the IDEG6 web tool using the Audic-Claverie statistic (AC), *p*-value < 0.01. Bonferroni multiple-testing correction was applied to consider only *p*-values < 3.0 × 10^-6 ^[53,60]. The comparison of root ESTs against all non-root ESTs resulted in the initial identification of 255 genes that had *p*-values below the significance threshold. Furthermore, the AC statistic identified 135 "root-enriched" transcripts that showed greater frequencies in root compared with non-root tissues as listed in Table 3. In addition, 119 of the 255 genes were identified as being enriched in the non-root libraries. Because a normalized root cDNA library was analyzed, these 119 genes were not considered further as the normalization process was expected to result in a systematic underrepresentation of highly abundant root transcripts. Evaluation of the functional categories of the 135 root-enriched genes showed that genes for primary and secondary metabolism as well as transport processes were more numerous compared with the entire root EST collection (Figure 6C).

**Table 3:** 135 genes with predicted root-enrichment expression profiles by Audic-Claverie statistic

Gene ID	Gene Description	Gene Function (via *Vitis*Net)	Root EST count (frequency)	Non-root EST count (frequency)	AC statistic
GSVIVP00030165001	Lectin	7.0 Stress-related	68 (40.1)	32 (1.2)	< 1E-06
GSVIVP00027836001	Curculin (mannose-binding) lectin	6.0 Binding	59 (34.8)	5 (0.2)	< 1E-06
**GSVIVP00024394001**	**Aquaporin TIP1;4**	**5.3 Transport System**	**57 (33.6)**	**2 (0.1)**	**< 1E-06**
GSVIVP00029248001	Aquaporin TMP-C	5.3 Transport System	57 (33.6)	15 (0.6)	< 1E-06
GSVIVP00015118001	Aspartic proteinase nepenthesin-1	2.3 Folding, Sorting & Degradation	47 (27.7)	38 (1.4)	< 1E-06
GSVIVP00036222001	Endochitinase 1, basic	1.0 Primary Metabolism	33 (19.5)	1 (0.1)	< 1E-06
GSVIVP00032953001	Glutamine synthetase (cytosolic) 2	1.2 Energy Metabolism	32 (18.9)	26 (1)	< 1E-06
**GSVIVP00018661001**	**Resveratrol O-methyltransferase**	**1.9 Secondary Metabolism**	**30 (17.7)**	**0 (0)**	**< 1E-06**
GSVIVP00013365001	Mannitol dehydrogenase	1.0 Primary Metabolism	26 (15.3)	26 (1)	< 1E-06
GSVIVP00006171001	Phosphate-induced protein 1	Unclear	26 (15.3)	37 (1.4)	< 1E-06
GSVIVP00015200001	Phosphate-induced protein 1	Unclear	24 (14.2)	5 (0.2)	< 1E-06
**GSVIVP00027842001**	**(E,E)-alpha-Farnesene synthase**	**1.9 Secondary Metabolism**	**23 (13.6)**	**0 (0)**	**< 1E-06**
GSVIVP00004164001	IAA beta-glucosyltransferase	3.2 Hormone Signaling	20 (11.8)	1 (0.1)	< 1E-06
GSVIVP00037746001	C2 domain-containing	3.1 Signal Transduction	20 (11.8)	7 (0.3)	< 1E-06
GSVIVP00036564001	Carboxylesterase	1.0 Primary Metabolism	19 (11.2)	0 (0)	< 1E-06
GSVIVP00011776001	Polyphenol oxidase II, chloroplast	5.3 Transport System	19 (11.2)	17 (0.7)	< 1E-06
GSVIVP00020241001	Unknown	Unknown	19 (11.2)	30 (1.1)	< 1E-06
GSVIVP00013172001	Octicosapeptide/Phox/Bem1p (PB1)	Unknown	18 (10.6)	12 (0.5)	< 1E-06
GSVIVP00030638001	Xyloglucan endotransglycosylase	4.3 Cell Wall	18 (10.6)	28 (1)	< 1E-06
GSVIVP00036411001	RD22	7.0 Stress-related	17 (10)	5 (0.2)	< 1E-06
GSVIVP00021415001	Glutathione S-transferase 8	1.0 Primary Metabolism	16 (9.4)	14 (0.5)	< 1E-06
GSVIVP00009226001	Stilbene synthase	1.9 Secondary Metabolism	15 (8.8)	7 (0.3)	< 1E-06
GSVIVP00017772001	ATP synthase beta chain 2	5.3 Transport System	15 (8.8)	18 (0.7)	< 1E-06
GSVIVP00025990001	Caffeic acid O-methyltransferase	1.9 Secondary Metabolism	14 (8.3)	22 (0.8)	< 1E-06
GSVIVP00011267001	Flavonol sulfotransferase	1.9 Secondary Metabolism	13 (7.7)	0 (0)	< 1E-06
GSVIVP00002185001	DNA-binding protein	2.4 Replication & Repair	13 (7.7)	1 (0.1)	< 1E-06
GSVIVP00036600001	Nitrite reductase	1.2 Energy Metabolism	13 (7.7)	5 (0.2)	< 1E-06
GSVIVP00034550001	Unknown protein	Unknown	13 (7.7)	7 (0.3)	< 1E-06
GSVIVP00018662001	Orcinol O-methyltransferase 2	1.9 Secondary Metabolism	12 (7.1)	0 (0)	< 1E-06
GSVIVP00022812001	Germin	8.0 Storage	12 (7.1)	0 (0)	< 1E-06
GSVIVP00019908001	7S globulin precursor, basic	2.3 Folding, Sorting & Degradation	12 (7.1)	4 (0.2)	< 1E-06
GSVIVP00021582001	E8 protein	3.2 Hormone Signaling	12 (7.1)	4 (0.2)	< 1E-06
GSVIVP00013571001	Strictosidine synthase	1.9 Secondary Metabolism	12 (7.1)	5 (0.2)	< 1E-06
GSVIVP00020905001	Aldose 1-epimerase	1.0 Primary Metabolism	12 (7.1)	10 (0.4)	< 1E-06
GSVIVP00002589001	Unknown protein	Unknown	12 (7.1)	11 (0.4)	< 1E-06
GSVIVP00004581001	Carboxyesterase 20	1.0 Primary Metabolism	11 (6.5)	1 (0.1)	< 1E-06
GSVIVP00027736001	4-Amino-4-deoxychorismate lyase	1.0 Primary Metabolism	11 (6.5)	1 (0.1)	< 1E-06
GSVIVP00036840001	Ferulate-5-hydroxylase	1.9 Secondary Metabolism	11 (6.5)	3 (0.1)	< 1E-06
GSVIVP00001860001	UDP-glucose:anthocyanidin 5,3-O-glucosyltransferase	1.9 Secondary Metabolism	11 (6.5)	4 (0.2)	< 1E-06
GSVIVP00032824001	Aspartic proteinase nepenthesin-2	2.3 Folding, Sorting & Degradation	11 (6.5)	4 (0.2)	< 1E-06
GSVIVP00023389001	WRKY DNA-binding protein 11	2.11 Transcription Factors	11 (6.5)	6 (0.3)	< 1E-06
GSVIVP00031491001	UDP-glucose glucosyltransferase	1.0 Primary Metabolism	10 (5.9)	1 (0.1)	< 1E-06
**GSVIVP00037558001**	**Flavonol O-glucosyltransferase**	**1.9 Secondary Metabolism**	**10 (5.9)**	**1 (0.1)**	**< 1E-06**
GSVIVP00036143001	Monooxygenase	Unclear	10 (5.9)	1 (0.1)	< 1E-06
GSVIVP00017017001	Trans-cinnamate 4-monooxygenase	1.9 Secondary Metabolism	10 (5.9)	2 (0.1)	< 1E-06
GSVIVP00033062001	Senescence-associated gene (SAG101)	4.2 Cell Growth & Death	10 (5.9)	3 (0.1)	< 1E-06
GSVIVP00018298001	Phosphate translocator protein2, plastid	5.3 Transport System	10 (5.9)	7 (0.3)	< 1E-06
GSVIVP00005745001	Octicosapeptide/Phox/Bem1p (PB1) domain	Unknown	10 (5.9)	13 (0.5)	< 1E-06
GSVIVP00005849001	Anthocyanidin 3-O-glucosyltransferase	1.9 Secondary Metabolism	10 (5.9)	16 (0.6)	< 1E-06
GSVIVP00036485001	CYP82C4	1.0 Primary Metabolism	9 (5.3)	1 (0.1)	< 1E-06
**GSVIVP00002954001**	**Cinnamyl-alcohol dehydrogenase**	**1.9 Secondary Metabolism**	**9 (5.3)**	**1 (0.1)**	**< 1E-06**
GSVIVP00031199001	Cytokinin-O-glucosyltransferase 2	1.9 Secondary Metabolism	9 (5.3)	3 (0.1)	< 1E-06
GSVIVP00015320001	**Nitrate reductase 2 (NR2)**	3.1 Signal Transduction	9 (5.3)	3 (0.1)	< 1E-06
GSVIVP00025346001	beta-1,3-Glucanase	1.0 Primary Metabolism	9 (5.3)	4 (0.2)	< 1E-06
GSVIVP00013928001	Phenylalanine ammonia-lyase	1.9 Secondary Metabolism	9 (5.3)	8 (0.3)	< 1E-06
GSVIVP00005194001	Stilbene synthase	1.9 Secondary Metabolism	9 (5.3)	8 (0.3)	< 1E-06
GSVIVP00001453001	Salt tolerance zinc finger	2.11 Transcription Factors	9 (5.3)	11 (0.4)	< 1E-06
GSVIVP00037055001	Metal-nicotianamine transporter YSL7	5.3 Transport System	9 (5.3)	11 (0.4)	< 1E-06
GSVIVP00020070001	Sulfate adenylyltransferase 3	1.2 Energy Metabolism	9 (5.3)	13 (0.5)	< 1E-06
GSVIVP00000463001	Cinnamyl alcohol dehydrogenase	1.9 Secondary Metabolism	9 (5.3)	14 (0.5)	< 1E-06
GSVIVP00024717001	Peroxidase	1.0 Primary Metabolism	8 (4.7)	0 (0)	< 1E-06
GSVIVP00031214001	Cytokinin-O-glucosyltransferase 2	1.9 Secondary Metabolism	8 (4.7)	0 (0)	< 1E-06
GSVIVP00034489001	2-Oxoglutarate-dependent dioxygenase	Unclear	8 (4.7)	0 (0)	< 1E-06
GSVIVP00036965001	Glutathione S-transferase 10 GSTU10	1.0 Primary Metabolism	8 (4.7)	1 (0.1)	< 1E-06
GSVIVP00018322001	Glucosyltransferase-2	1.0 Primary Metabolism	8 (4.7)	2 (0.1)	< 1E-06
GSVIVP00019233001	Isoflavone reductase	1.9 Secondary Metabolism	8 (4.7)	2 (0.1)	< 1E-06
GSVIVP00029527001	Unknown protein	Unknown	8 (4.7)	2 (0.1)	< 1E-06
GSVIVP00003722001	Zinc finger (C3HC4-type RING finger)	2.11 Transcription Factors	8 (4.7)	3 (0.1)	< 1E-06
GSVIVP00010417001	Zinc finger (C3HC4-type RING finger)	6.0 Binding	8 (4.7)	5 (0.2)	< 1E-06
GSVIVP00034781001	Kelch repeat-containing F-box	Unknown	8 (4.7)	5 (0.2)	< 1E-06
GSVIVP00020913001	Aldose 1-epimerase	1.0 Primary Metabolism	8 (4.7)	6 (0.3)	< 1E-06
GSVIVP00022605001	Nicotianamine synthase	1.0 Primary Metabolism	8 (4.7)	7 (0.3)	< 1E-06
GSVIVP00023306001	p-Coumaroyl shikimate 3'-hydroxylase 1	1.9 Secondary Metabolism	8 (4.7)	7 (0.3)	< 1E-06
GSVIVP00002706001	Unknown protein	Unknown	8 (4.7)	7 (0.3)	< 1E-06
GSVIVP00031130001	DNA-damage-repair/toleration (DRT102)	2.4 Replication & Repair	8 (4.7)	8 (0.3)	< 1E-06
GSVIVP00024773001	Acyl-CoA synthetase (Acyl-activating 18)	5.3 Transport System	8 (4.7)	9 (0.4)	< 1E-06
GSVIVP00000809001	Phosphoesterase	Unclear	8 (4.7)	11 (0.4)	< 1E-06
GSVIVP00010326001	Esterase/lipase/thioesterase family	Unclear	8 (4.7)	12 (0.5)	2E-06
GSVIVP00015215001	UDP-glycosyltransferase 85A1	1.0 Primary Metabolism	7 (4.1)	0 (0)	< 1E-06
GSVIVP00026343001	NADPH HC toxin reductase	1.0 Primary Metabolism	7 (4.1)	0 (0)	< 1E-06
GSVIVP00036190001	Catechol O-methyltransferase	1.0 Primary Metabolism	7 (4.1)	0 (0)	< 1E-06
GSVIVP00010293001	F-box domain containing	2.3 Folding, Sorting & Degradation	7 (4.1)	0 (0)	< 1E-06
GSVIVP00025242001	Aspartyl protease	2.3 Folding, Sorting & Degradation	7 (4.1)	0 (0)	< 1E-06
GSVIVP00026388001	Pectinesterase family	4.3 Cell Wall	7 (4.1)	0 (0)	< 1E-06
GSVIVP00015805001	AT-hook DNA-binding protein	Unknown	7 (4.1)	0 (0)	< 1E-06
**GSVIVP00008086001**	**Myb family TF-like b**	**2.11 Transcription Factors**	**7 (4.1)**	**1 (0.1)**	**< 1E-06**
GSVIVP00017803001	Laccase	5.3 Transport System	7 (4.1)	1 (0.1)	< 1E-06
GSVIVP00036529001	Open stomata 1 (OST1)	3.1 Signal Transduction	7 (4.1)	2 (0.1)	< 1E-06
GSVIVP00024338001	Fasciclin arabinogalactan-protein (FLA4)	4.3 Cell Wall	7 (4.1)	2 (0.1)	< 1E-06
GSVIVP00024285001	Zinc transporter (ZIP2)	5.3 Transport System	7 (4.1)	2 (0.1)	< 1E-06
GSVIVP00017555001	UDP-glycosyltransferase 89B2	1.0 Primary Metabolism	7 (4.1)	3 (0.1)	< 1E-06
GSVIVP00018198001	Patatin	8.0 Storage	7 (4.1)	3 (0.1)	< 1E-06
GSVIVP00029089001	Kelch repeat-containing	Unknown	7 (4.1)	3 (0.1)	< 1E-06
GSVIVP00012218001	Myb divaricata	2.11 Transcription Factors	7 (4.1)	4 (0.2)	< 1E-06
GSVIVP00031610001	Unknown protein	Unknown	7 (4.1)	4 (0.2)	< 1E-06
GSVIVP00023356001	alpha-L-Arabinofuranosidase	1.0 Primary Metabolism	7 (4.1)	5 (0.2)	< 1E-06
GSVIVP00028303001	beta-1,3-Glucanase precursor	1.0 Primary Metabolism	7 (4.1)	5 (0.2)	< 1E-06
GSVIVP00030576001	Receptor-like protein kinase	3.1 Signal Transduction	7 (4.1)	5 (0.2)	< 1E-06
GSVIVP00019610001	IAA-amido synthetase GH3.2	3.2 Hormone Signaling	7 (4.1)	5 (0.2)	< 1E-06
GSVIVP00024987001	Allergen Alt a 7	7.0 Stress-related	7 (4.1)	5 (0.2)	< 1E-06
GSVIVP00002843001	Kelch repeat-containing F-box	Unknown	7 (4.1)	5 (0.2)	< 1E-06
GSVIVP00001138001	Flavonoid 3-monooxygenase	1.9 Secondary Metabolism	7 (4.1)	6 (0.3)	< 1E-06
GSVIVP00021523001	Aspartyl protease	2.3 Folding, Sorting & Degradation	7 (4.1)	6 (0.3)	< 1E-06
GSVIVP00023266001	Serine carboxypeptidase K10B2.2	2.3 Folding, Sorting & Degradation	7 (4.1)	8 (0.3)	< 1E-06
GSVIVP00003796001	Glycosyl hydrolase family 1	1.0 Primary Metabolism	6 (3.5)	0 (0)	< 1E-06
GSVIVP00005841001	UDP-glucose glucosyltransferase	1.0 Primary Metabolism	6 (3.5)	0 (0)	< 1E-06
GSVIVP00006924001	Peroxidase	1.0 Primary Metabolism	6 (3.5)	0 (0)	< 1E-06
GSVIVP00023878001	CYP94A1	1.0 Primary Metabolism	6 (3.5)	0 (0)	< 1E-06
GSVIVP00023969001	Class III peroxidase 40	1.0 Primary Metabolism	6 (3.5)	0 (0)	< 1E-06
GSVIVP00037866001	Peroxidase	1.0 Primary Metabolism	6 (3.5)	0 (0)	< 1E-06
**GSVIVP00006201001**	**AP2/ERF114**	**2.11 Transcription Factors**	**6 (3.5)**	**0 (0)**	**< 1E-06**
GSVIVP00033054001	Protein phosphatase 2C	3.1 Signal Transduction	6 (3.5)	0 (0)	< 1E-06
GSVIVP00000122001	Chromosome maintenance MAG2	2.4 Replication & Repair	6 (3.5)	1 (0.1)	< 1E-06
GSVIVP00018988001	Transposon protein	9.0 Transposons	6 (3.5)	1 (0.1)	< 1E-06
GSVIVP00014792001	Carboxylesterase	1.0 Primary Metabolism	6 (3.5)	3 (0.1)	< 1E-06
GSVIVP00033506001	beta-1,3-Glucanase	1.0 Primary Metabolism	6 (3.5)	3 (0.1)	< 1E-06
GSVIVP00014758001	Calmodulin-binding protein AR781	3.1 Signal Transduction	6 (3.5)	3 (0.1)	< 1E-06
GSVIVP00019639001	Peroxidase 73	1.0 Primary Metabolism	6 (3.5)	4 (0.2)	< 1E-06
GSVIVP00009234001	Stilbene synthase	1.9 Secondary Metabolism	6 (3.5)	4 (0.2)	< 1E-06
GSVIVP00020035001	MLK/Raf-related protein kinase 1	3.1 Signal Transduction	6 (3.5)	4 (0.2)	< 1E-06
**GSVIVP00025363001**	**Myb family TF-like a**	**2.11 Transcription Factors**	**5 (2.9)**	**0 (0)**	**< 1E-06**
**GSVIVP00026190001**	**NGA1 TF (NGATHA1)**	**2.11 Transcription Factors**	**5 (2.9)**	**0 (0)**	**< 1E-06**
GSVIVP00037318001	Myb divaricata	2.11 Transcription Factors	5 (2.9)	0 (0)	< 1E-06
GSVIVP00007503001	ACC oxidase homolog 1	3.2 Hormone Signaling	5 (2.9)	0 (0)	< 1E-06
GSVIVP00006975001	Kinesin family member 2/24	4.1 Cell Motility	5 (2.9)	0 (0)	< 1E-06
GSVIVP00021432001	Laccase	5.3 Transport System	5 (2.9)	0 (0)	< 1E-06
GSVIVP00012703001	Aquaporin TIP2;2	5.3 Transport System	5 (2.9)	0 (0)	< 1E-06
GSVIVP00008708001	Monooxygenase (MO3)	1.0 Primary Metabolism	5 (2.9)	0 (0)	< 1E-06
GSVIVP00020827001	AAA-type ATPase	Unclear	5 (2.9)	0 (0)	< 1E-06
GSVIVP00017947001	Unknown protein	Unknown	5 (2.9)	0 (0)	< 1E-06
GSVIVP00021666001	Unknown protein	Unknown	5 (2.9)	0 (0)	< 1E-06
GSVIVP00001266001	Unknown	Unknown	5 (2.9)	0 (0)	< 1E-06
GSVIVP00017730001	CYP77A5P	1.0 Primary Metabolism	5 (2.9)	1 (0.1)	< 1E-06
GSVIVP00006293001	Jasmonate O-methyltransferase	3.2 Hormone Signaling	5 (2.9)	1 (0.1)	< 1E-06
GSVIVP00020849001	ABC transporter B member 11	5.3 Transport System	5 (2.9)	1 (0.1)	< 1E-06

**Figure 7:**
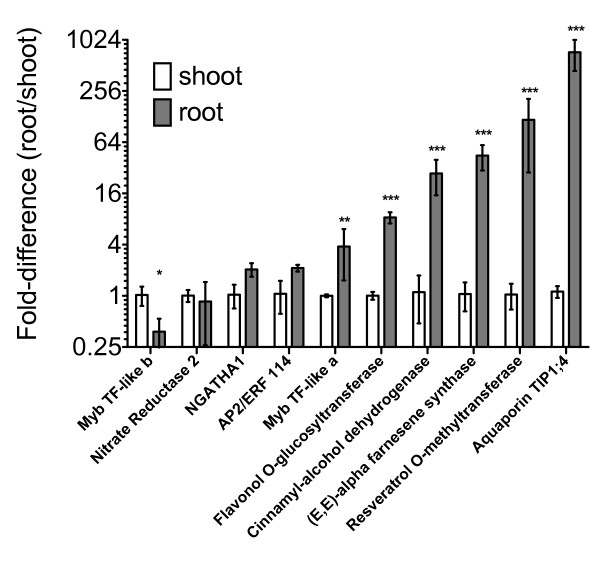
**Expression of candidate root-specific genes in roots and shoots of Cabernet Sauvignon**. qRT-PCR analysis of ten selected transcripts in shoot (white bars) and root (gray bars) tissues. Transcript abundances derived from three biological replicates were normalized to an actin reference gene and fold differences were standardized to shoot expression values. Error bars represent standard error. Two-way ANOVA (gene, tissue) was performed followed by post-test Bonferroni-corrected *t-*statistics. Significant differences in gene expression (root compared with shoot) are indicated by asterisks. * denotes p < 0.05; ** denotes p < 0.01; *** denotes p < 0.001. Fold-differences are drawn on log scale. The tested genes are listed below in the order that they appear on the graph from left to right, with the number of root ESTs compared with non-root ESTs in parentheses. Myb family transcription factor-like b, (7 compared with 1); Nitrate reductase 2, (9 compared with 3); NGATHA1 transcription factor, (5 compared with 0); (AP2/ERF transcription factor, 6 compared with 0); Myb family transcription factor-like a, (5 compared with 0); Flavonol 3-O-glucosyltransferase, (10 compared with 1); Cinnamaldehyde dehydrogenase, (9 compared with 1); (*E, E*)-alpha-Farnesene synthase, (23 compared with 0); Resveratrol O-methyltransferase, (30 compared with 0); Aquaporin TIP1;4, (57 compared with 2).

Root-enriched genes were identified by EST frequency comparison of *Vitis vinifera *roots compared with all other tissues, using the Audic-Claverie (AC) statistic [60]. Gene identifier (ID) from GSVIV, gene description, gene function (from *Vitis*Net annotation), frequency in root and non-root cDNA libraries, and AC confidence statistic are presented. Genes tested for root-enriched expression by real-time qRT-PCR are indicated in bold (Figure 7).

### Validation of root-enriched genes

In order to confirm root expression patterns estimated by EST frequency, the expression of a set of putative root-specific or root-enriched genes was selected for validation by qRT-PCR. Gene-specific primers were designed for ten of the 135 highly root-enriched transcripts. Genes were selected not only for those with very high root EST count, but also for those gene with lower frequencies, but still considered statistically significant. The gene-specific primers used are listed in Additional File 6. Relative transcript abundance for each gene was tested within root and shoot tissue of Cabernet Sauvignon (Figure 7). Two-way ANOVA by gene and tissue was performed, and both were significant (P < 0.0001). After ANOVA, individual Bonferroni corrected t-statistics were computed for each individual gene between root and shoot tissues. Of these ten transcripts, six were found to be significantly more abundant in roots than shoots by Student's t-statistic (p < 0.01). Transcript abundances ranged from 3.8- to 730-times greater abundance in roots than shoots.

The most highly root-enriched transcript encoded an uncharacterized *Vitis *tonoplast intrinsic protein TIP1;4 (GSVIVP00024394001) and was detected at 730-times greater transcript abundance in roots than in shoot tissue. This correlates well with the estimated expression by EST frequencies, where it was found with a frequency of ~33.6 tags per ten thousand (tp10k) in roots compared with 0.1 tp10k in non-root tissues (57 root ESTs compared with 2 non-root ESTs). A resveratrol O-methyltransferase (ROMT, GSVIVP00018661001) that was found with a frequency of 17.7 tp10k in roots (30 root ESTs compared with 0 non-root EST) was expressed 120-fold greater in root than in shoot as estimated by qRT-PCR. Similarly, a terpene synthase (TPS) gene, (*E, E*)-alpha-farnesene synthase [61], was found with a frequency of 13.6 tp10k in roots (23 root ESTs compared with 0 non-root EST) and was 44-fold more abundant in root than shoot as assessed by qRT-PCR. A cinnamyl-alcohol dehydrogenase (9 root ESTs compared with 1 non-root EST) was expressed 27-fold greater in roots than in shoots. A flavonol 3-O-glucosyltransferase (10 root ESTs compared with 1 non-root EST) showed a 8.3-fold greater abundance in roots than in shoots. Lastly, a Myb transcription factor-like a gene (5 root ESTs compared with 0 non-root EST) was tested to evaluate the selected significance cutoff. This transcript was detected at 3.8-fold greater abundance in roots than in shoots (significant, p < 0.05). In contrast, three of the genes tested (e.g., AP2/ERF114, NGATHA1, Nitrate Reductase 2) failed to demonstrate a significant difference as measured by the multiple test-corrected t-statistic, and a single transcript, a second Myb transcription factor-like b gene (7 root ESTs compared with 1 non-root EST), was determined to be 2.6-fold less abundant in roots than in shoots (p < 0.05) (Figure 7). For all ten genes tested, the Spearman rank correlation between the two measures of gene expression (EST frequency compared with qRT-PCR) was high (*r_s _*= 0.78, p = 0.005). Although only ten genes were tested, estimation of transcript abundance by EST frequency was apparently effective in identifying genes with root-specific expression, despite the majority of root ESTs coming from a normalized library source.

## Discussion

### Data mining to discover *Vitis vinifera *stress-adaptive genes

In order to identify novel transcripts that respond to multiple environmental stress treatments, EST libraries generated by us and those derived from public sources were carefully curated and mined to obtain estimates of transcript abundance based on EST frequencies. A total of 21,499 and 18,963 unique ESTs derived from non-normalized cDNA libraries from mixed abiotic stress leaf and water-deficit stressed berry tissues, respectively, were compared with 5277 and 24,953 unique ESTs derived from cDNA libraries generated with unstressed leaf and berry tissues (Table 1). Tag frequency-based detection of differentially expressed genes is a well-established methodology for ESTs [53,60,62], SAGE [57], and MPSS [7], and continues to be an important tool in the era of "next-generation" deep sequencing of transcriptomes [63]. Aside from the removal of redundant ESTs derived from bi-directional and/or same direction resequencing of individual cDNA clones, one of the main issues encountered during the data curation process was the discovery of various types of naming errors within and across plated clone libraries. With the aid of a simple dot-plot method analogous to that used for local nucleotide sequence alignments [51], gene IDs could be aligned and readily visualized to discover incorrectly paired plates (or portions of plates) containing "well slip" naming errors that would have overestimated the number of ESTs actually present within a particular cDNA library of interest due to duplicated sequencing of plates within the same library (Table 2, Figure 1A-G). Application of this technique also allowed for the discovery of a misassigned plate of ESTs from a leaf cDNA library to a berry cDNA library, an error that would have confounded the accuracy of EST counting with regard to a particular tissue (Figure 1H).

Comparing EST frequency counts from cDNA libraries of mixed or water-deficit stressed leaf and berry tissues, respectively, with those from cDNA libraries from unstressed leaf and berry tissues, a total of 739 transcripts were identified and clustered into four main clusters (Figure 2, Additional Files 1, 2, 3 and 4). Of these, 637 (86%) transcripts could be annotated and assigned to functional categories (Figure 3). Each cluster contained distinct functional groups that reflected clearly the tissue type and treatment condition in question. For example, transcripts encoding the CBL-interacting protein kinase 10 (CIPK10) were overrepresented in both the stressed leaf (SL) and stressed berries (SB) clusters. CIPK10 participates in the calcineurin B-like (CBL) calcium sensor protein-CIPK network that decodes calcium signals in response to environmental perturbations [64]. The *Arabidopsis *CIPK10 is localized to the nucleus and cytoplasm when expressed as a GFP fusion in *Nicotiana benthamiana *leaves [65]. CBL-CIPK interactions are crucial for the regulation of ion homeostasis during salinity stress and other forms of environmental stress, not only at the plasma membrane and tonoplast, but also at the cytoplasm, and nucleus [65]. The increased abundance of CIPK10 transcripts in these stress-specific cDNA libraries indicates this CIPK might play a role in stress adaptation in both *Vitis *leaves and berries. Several other stress-specific transcripts appeared to be over-represented in both stress libraries including RD22, a salt-, dehydration-, and ABA-responsive gene in grape berries [66] (Additional File 1 and 3). In addition to the genes discussed earlier that were enriched within the stressed berry (SB) cluster, several pathogenesis-related (PR) proteins, such as three thaumatin genes, a class IV chitinase gene, two osmotin genes, and Snakin-1, a cysteine-rich peptide that exhibits broad-spectrum antimicrobial activity *in vitro *and fungal and bacterial pathogen resistance *in vivo *[67], were also enriched in this cluster. The identification of this collection of PR proteins using the EST frequency counting approach outlined here clearly illustrates its practical utility in the discovery of genetic determinants important for biotic and abiotic stress responses. A large number of unknown genes with discrete, cluster-specific expression patterns were also identified, particularly within the stressed leaf (SL) cluster. Such unknown genes can serve as primary targets for future, detailed investigations into gene function.

### Validation of EST frequency counts by microarray analysis

In order to validate the efficacy of the EST frequency counting method, 489 out of 739 transcripts could be identified on the Affymetrix^® ^*Vitis *GeneChip^® ^microarray and thus compared using these two distinct technical approaches. The remaining 250 transcripts had no match, and thus, were potentially not described previously as being abiotic stress responsive in *Vitis*. Between the two platforms, expression data for 184 transcripts could be compared where significant differences in gene expression patterns were observed using both technologies. Like previous reports comparing tag and hybridization measures [63], a modest (*r *= 0.21), but significant correlation between the two platforms was observed (Figure 4). Further comparison between the two methods revealed a directional concordance of 69%, indicating that the two platforms agreed to a greater extent in terms of their general gene expression trends. What might account for these rather modest correlations? First, these low correlations might be related partly to differences in the reported magnitude of increased or decreased transcript abundance. However, for every two genes that were reported increased or decreased significantly by both platforms, one gene changed significantly in opposite directions (Figure 4). Thus, magnitude can only account for part of the disagreement. Second, the use of public data sets, which are highly diverse, might introduce biases in gene representation. In earlier studies that have mined public datasets, such as in a comparison of EST reads generated by 454 pyrosequencing with microarray mRNA profiles in two porcine tissues, four-to-one concordance (160 compared with 38) ratios were observed [63] or in a comparison of SAGE tags with microarrays mRNA profiles within a set of human tissues, three-to-one concordance ratios were observed [58]. In the present study, while major systematic errors within the public data sets were corrected in an attempt to capture correct frequency counts for unstressed leaf and berry libraries (Figure 1; Tables 1, 2), these public data sets contained large differences in grapevine cultivar, age, developmental stage, season, terroir, and sample preparation that were likely to introduce biases in gene representation. Third, the relative complexity of our mixed stress leaf library might be a source of bias, because the source tissue for this library included RNA from UV- and heat-treated leaves, treatments for which corresponding microarray data were unavailable for comparison. The presence of genes strongly or exclusively regulated by UV or heat stress would be expected to contribute to the population of the significant-by-EST transcripts with which no corresponding microarray data could be compared.

### EST-based gene discovery in *Vitis *roots

To redress the relative paucity of available grape root sequence data, more than 16,000 ESTs were generated from a normalized cDNA library (VVM) constructed from Cabernet Sauvignon root tissues exposed to cold, salinity, and water deficit stress (Table 1). During its preparation, this library was normalized with the aim of increasing the number of different and low-abundance root genes identified [68]. The 16,452 ESTs assemble into 6424 unique transcripts, of which 3449 (>53%) were represented just once. Because normalized libraries are biased, resulting in an under-counting of abundant transcripts and over-counting of rare ones, they violate the assumption of random sampling, and as such, are not usually considered for use in tag frequency analyses of gene expression [6,42]. Recognizing that library normalization would, at a minimum, underestimate the true relative expression of most root transcripts, the identification of root-specific or root-abundant EST was attempted by EST frequency counting. A total of 18,109 root-derived ESTs were compared with 291,233 ESTs from 114 non-root cDNA libraries. This analysis resulted in the identification of 135 "root-enriched" transcripts with significantly greater EST frequencies in roots than other tissues as determined by the AC statistic (Table 3). Validation of a set of 10 candidate root genes with varying degrees of apparent root enrichment by qRT-PCR confirmed six genes to be significantly more abundant in grapevine roots than in shoots (Figure 7). The correlation between estimated EST frequencies and qRT-PCR expression ratios was strong (*r_s _*= 0.78) and significant (P = 0.005). Shoot tissue was used to confirm broadly, but not exhaustively, that expression patterns were root-enhanced. Confirmation of the root-specific expression patterns of these candidate genes will require that additional non-root tissue types (e.g., stems, flowers, berries, etc.) be tested on a gene-by-gene basis.

Chief among the qRT-PCR-validated root genes is a gene encoding an aquaporin/tonoplast intrinsic protein 1;4 (*Vv*TIP1;4) that was expressed as much as 730-fold more in roots than in shoots. *Vv*TIP1;4 has been previously identified from genomic sequence by two groups [69,70], but has not yet been characterized functionally. Another root-enriched gene, which showed 120-fold greater mRNA abundance in roots than in shoots by qRT-PCR, encodes a putative resveratrol-O-methyltransferase (ROMT), which is 78% identical and 88% similar to a known *Vitis *ROMT [71]. The ROMT characterized by Schmidlin and colleagues was observed to doubly O-methylate molecules of resveratrol into pterostilbene, a phytoalexin with 5-10 times greater *in vitro *fungitoxicity than resveratrol [71]. This root-expressed ROMT is also structurally distinct from a ROMT recently characterized in red berries. The red berry ROMT transcript was more abundant in the red grape Cabernet Sauvignon than the white Chardonnay and had peak expression two weeks after véraison in the red cultivar only [72]. A terpene synthase (TPS) was highly expressed in roots with a 44-fold greater relative abundance in root than in shoots. Martin and colleagues identified this TPS to be an (*E, E*)-alpha-farnesene synthase in a thorough survey to characterize *V. vinifera *TPS genes [61]. This TPS exhibited activity that was unique among the 39 characterized, producing only (*E, E*)-alpha-farnesene when fed farnesene diphosphate (FPP), rather than a mixture of multiple products. A cinnamyl-alcohol dehydrogenase (CAD) gene was also confirmed to be 27-fold more abundant in roots than in shoots. CAD genes are crucial for the synthesis of the lignin compounds in wood formation, but some CAD genes might possess other activities or functions. In *Arabidopsis*, the activity of the promoters of some AtCAD genes has been observed in cells where CAD-mediated lignification does not appear to take place, including young root tips [73]. Lastly, an UDP-Glucose O-glucosyltransferase (UGT) gene was 8.3-fold more abundant in roots than in shoots. When compared to the position-specific scoring matrices (PSSMs) found in NCBI's Conserved Domain Database (CDD) [74], this UGT was most similar to the PLN02554 group of UGTs, which are classified as flavonol 3-O-glucosyltransferases (EC 2.4.1.91). However, determining the exact catalytic activities of UGTs generally requires biochemical characterization as even single amino acid changes in UGT proteins can alter regioselectivity (e.g., which hydroxyl group is glycosylated) or UDP-sugar substrate preference [75,76]. Four other candidate genes were also surveyed, but none were found to exhibit significant, root-enriched mRNA expression at p < 0.01.

## Conclusions

Abiotic stresses, especially water-deficit stress, have major impacts on vine growth and berry development that ultimately can impact wine quality. Here, EST frequency counts were exploited to identify candidate genes with mRNA expression profiles altered by abiotic stresses by comparing large EST collections from cDNA libraries prepared from leaf and berries harvested from vines subjected to mixed abiotic stresses to publicly available EST collections from these same tissues harvested from unstressed vines. This analysis identified 739 transcripts with significant differential expression in abiotically stressed leaves and berries. Comparison of EST frequency counts of these genes with available microarray expression data identified 184 genes, which also showed significant differences between stressed, and unstressed tissues. While the correlation in expression patterns was modest at best, 69% of genes exhibited directional concordance. Furthermore, the EST frequency counting approach led to the identification of many novel candidate genes whose stress-induced mRNA expression patterns had not been described previously. To identify genes preferentially or exclusively expressed in *Vitis *roots, a tissue that had previously been largely uncharacterized, 16,452 EST were characterized from a normalized, abiotically stressed cDNA library from Cabernet Sauvignon. Comparison of these ESTs with publicly available EST collections from non-root tissues allowed for the identification of 135 root-enriched transcripts, a majority of which showed root-preferential mRNA expression when validated by qRT-PCR. This root-enriched EST collection will serve as a rich resource not only for future studies into the abiotic stress-response networks operating within roots, but also for future genotyping efforts of *Vitis *rootstock that differ in salinity or drought tolerance characteristics or for manipulation of root stock traits in wine grape.

## Methods

### Plant material

Total RNA was extracted from abiotically stressed *V. vinifera *cv. Chardonnay leaf and berry tissue 8, 9, 11, 13, 15, 16 weeks after flowering) using a modified Tris-LiCl protocol as previously described [77]. Root tissue was collected from 10 cm high *V. vinifera *cv. Cabernet Sauvignon cuttings grown in autoclaved, sterile 77 mm × 77 mm × 97 mm (W × L × H) Magenta GA-7 boxes (Magenta Corp., Chicago, IL) containing 80 ml of 1% Plant Tissue Culture Agar (#A111, Phytotechnology Laboratories, Shawnee Mission, KS) with Murashige & Skoog modified Basal Medium w/ Gamborg Vitamins (#M404, Phytotechnology Laboratories), 1.5% sucrose at pH 5.7 [78,79] grown under fluorescent lamps providing a photon flux density of 50 μmol m^-2 ^s^-1 ^on a 16-h light (24°C)/8-h dark (18°C) cycle. Roots were detached from non-stressed plants and subjected to control conditions (bathed in liquid MS media as above), water deficit stress conditions by exposure to air (for 2 and 4 h), cold (1.5°C), and 150 mM NaCl (in liquid MS media as above) stress for 2, 4 and 6 h. The 6 h time point for water-deficit stress exposure was not used because intact RNA could not be recovered from root tissue after 4 h of stress.

### Leaf and Berry cDNA Library Construction, sequencing and processing

The preparation of the leaf (Library ID 10208) and berry (Library ID 12534) cDNA libraries was described previously [6]. The frozen, ground tissue of Chardonnay leaf and berry were homogenized in a buffer containing 200 mM Tris-HCl, pH = 8.5, 1.5% (w/v) lithium dodecyl sulfate, 300 mM LiCl, 10 mM sodium EDTA, 1% w/v sodium deoxycholate, and 1% v/v NP-40. Following autoclaving, 2 mM aurintricarboxylic acid, 20 mM dithiotheitol (DTT), 10 mM thiourea, and 2% w/v polyvinylpolypyrrolidone were added immediately before use. Following precipitation with sodium acetate and isopropanol precipitation, samples were extracted once with 25:24:1 phenol:chloroform:isoamyl and then twice with 24:1 chloroform:isoamyl prior to performing LiCl precipitations to remove DNA contamination. Poly(A)+ RNA was purified from 500 mg of total RNA using the Micro-FastTrack™ 2.0 mRNA Isolation Kit (Invitrogen, Inc., Carlsbad, CA) according to the manufacturer's instructions. cDNA was synthesized from 1-5 μg of poly(A)+ RNA using a Lambda Uni-Zap-XR cDNA synthesis kit according to the manufacturer's recommended protocol (Stratagene, La Jolla, CA). The directionally cloned (EcoRI/XhoI) cDNA libraries generated were then mass-excised *in vivo *and the resulting plasmids (pBluescript II) were propagated in the *E. coli *SOLR host strain. Individual cDNA clones containing inserts were amplified using the TempliPhi DNA Sequencing Template Amplification kit (Amersham Biosciences Corp., Piscataway, NJ) and sequenced using the dideoxy chain-termination method on an Applied Biosystems 3700 automated DNA sequencing system using the Prism™ Ready Reaction Dyedeoxy™ Terminator Cycle Sequencing kit (Applied Biosystems Division, Perkin-Elmer, Foster City, CA). The T3 primer (5'- GGGAAATCACTCCCAATTAA-3') and the T7 primer (5'-GTAATACGACTCACTATAGGGC-3') were used for 5' reads and 3' reads of cDNA clones, respectively. Oligo-dT primer (T_22_M) was used for 3' sequencing reads of cDNA clones containing poly-A tails.

Raw single-pass sequence data were retrieved from a Geospiza Finch server and downloaded to the EST Analysis Pipeline (ESTAP) [80] for cleansing and analysis. Following removal of vector and low quality sequences, all sequences ≤ 50 bp in length were discarded. Remaining sequences were clustered using d2_cluster [81] and CAP3 algorithms [82] using default parameters established for ESTAP.

### Root cDNA library construction

A third mixed cDNA library ("VVM", Library ID 22274) was constructed using total RNA from cold, water-deficit, 150 mM NaCl stressed and control condition roots. Total RNAs from different treatments were extracted and equal quantities were pooled before mRNA selection. Poly(A)+ mRNA was isolated from total RNA using the Oligotex Direct mRNA kit (Qiagen, Valencia, CA). cDNA synthesis was conducted by converting poly(A)+ mRNA to double-stranded cDNA with the 5'-AACTGGAAGAATTCGCGGCCGCTCGCATTTTTTTTTTTTTTTTTTV-3' (V = A,C,G) primer and Superscript III reverse transcriptase (Invitrogen). Double-stranded cDNAs were size-selected (more than 600 bp), modified with EcoRI adaptors (AATTCCGTTGCTGTCG - Promega #C1291) and digested with NotI. The cDNAs were then directionally cloned into EcoRI-NotI digested pBluescript II SK+ phagemid vector (Stratagene, Inc., La Jolla, CA). The total number of white colony forming units (cfu) before amplification was 3.0 × 10^6^. Blue colonies (empty vectors) were less than 10% of the total colonies present on plates. Purified plasmid DNA from the primary library was converted to single-stranded circles and used as the template for PCR amplification using the T7 (5'-TAATACGACTCACTATAGGG-3') and T3 (5'-AATTAACCCTCACTAAAGGG-3') priming sites flanking the cloned cDNA inserts as previously described [68]. The purified PCR products, representing the entire cloned cDNA population, were used as a driver for normalization. Hybridization between the single-stranded library (50 ng) and the PCR products (500 ng) was carried out for 44 hours at 30°C. Unhybridized single-stranded DNA circles were separated from hybridized DNA rendered partially double-stranded and electroporated into *Escherichia coli *DH10B cells to generate the normalized library. The total number of clones with insert was 1.6 × 10^6 ^cfu. Background levels of empty clones were less than 10%. cDNA library normalization and construction was performed by the W.M. Keck Center for Comparative and Functional Genomics at the Roy J. Carver Biotechnology Center at the University of Illinois at Urbana-Champaign. Normalization efficiency was verified by random sampling and sequencing of 96 and 285 clones from both the primary and the normalized libraries, respectively, and comparing their redundancy rates.

### Root EST sequencing and data analysis

EST sequencing of the normalized root cDNA library was performed using a T7 sequencing primer (5'-TAATACGACTCACTATAGGG-3') on either an Applied Biosystems 3700 automated DNA sequencing system (Applied Biosystems Division, Perkin-Elmer) at Beckman Coulter, Inc., Genomic Services (Danvers, MA; formerly Agencourt Biosciences, Inc. Beverly, MA) or on Beckman CEQ8000 and CEQ8800 sequencers (Beckman Coulter Inc., Brea, CA) at the Central Lab of the Biotechnology Institute, Ankara University. Sequence chromatograms were processed through *phred *[83] for high-quality base-calls, and screened/masked to omit vector sequence using *cross_match *(-minmatch 10 -minscore 20 -masklevel 100) against NCBI's UniVec with added screening and removal of sequences specific to the cloning adaptor strategy. To precisely identify and fully mask the vector/adaptor region 5' to the inserted cDNA fragment the "canonical adaptor region" (5'-TTGTAAAACGACGGCCAGTGAATTGTAATACGACTCACTATAGGGCGAATTGGGTACCGGG

CCCCCCCTCGAGGTATAAGCTTGATATCGAATTCCGTTGCTGTCG-3'), "2variant39" (5'-GCTTGATATCGAATTCCGTTGCTAATTCCGTTGCTGTCG-3'), "3variant51" (5'-GCTTGATATCGAATTCCGTTGCTGTCGCCGTTGCTGTCTCCGTTGCTGTCG-3'), and "4variant39" (5'-GCTTGATATCGAATTCCGTTGCTGTCGCCGTTGCTGTCG-3') sequences were added to the vector screen file. To detect and mask TGCGA-tagged/NotI/vector regions 3' to the inserted EST, "pB SK- at NotI site" (5'-TGCGAGCGGCCGCCACCGCGGTGGAGCTCCAGCTTTTGTTCCCTTTAGTGAGGGTTAATTTCGA

GCTTGGCGTAATCATGGTCATAGCTGTTTCC-3') and the variant (5'-GATCAGCGGCCGCCACCGCGGTGGAGCTCCAGCTTTTGTTCCCTTTAGTGAGGGTTAATTTCGA

GCTTGGCGTAATCATGGTCATAGCTGTTTCC-3') sequences were added to the vector screen file. A set of Perl programs was designed to process sequences for minimum length (>100 nt), chimera removal, poly-A tail signal identification, Basic Local Alignment Search Tool (BLAST) annotation, and dbEST submission. The submitted high-quality ESTs were provisionally given annotations of each top BLAST hit compared with *nr *(version 11.06.2007) [84]. The root ESTs from library VVM were submitted to dbEST and were assigned the Genbank IDs FC054794-FC071210, and FC072669-FC072703. The library was submitted to dbEST as "VVM" http://www.ncbi.nlm.nih.gov/UniGene/library.cgi?ORG=Vvi&LID=22274[85].

### Datasets used, clustering analysis and annotation

All available *V. vinifera *sequences (including ESTs, expressed transcripts as well as other available DNA sequences in the NCBI database) were extracted from GenBank with Batch Entrez at NCBI (http://www.ncbi.nlm.nih.gov/sites/batchentrez) [86]. Additional information for cDNA libraries was obtained from the NCBI UniGene grape database (http://www.ncbi.nlm.nih.gov/UniGene/UGOrg.cgi?TAXID=29760) [48]. ESTs sequences were then associated with their corresponding "tentative consensus" (TC) contig sequence from the *V. vinifera *Gene Index (VvGI, version 6, Dana Farber Cancer Institute, http://compbio.dfci.harvard.edu/tgi/cgi-bin/tgi/gimain.pl?gudb=grape) [49,59]. Libraries analyzed are listed in Table 1. Assembled TC sequences or individual singleton where no TC could be assigned were then compared to the predicted peptide sequences from the Genoscope 8.4X *V. vinifera *cv. Pinot Noir (GSVIV) genome assembly (http://www.genoscope.cns.fr/externe/Download/Projets/Projet_ML/data/) [1,87]. Database searches using the BLAST were performed using the Tera-BLAST™P algorithm, open penalty 11, extend penalty 1, double-affine Smith-Waterman window 50, and maximum e-value cutoff 1 × 10^-3 ^on TimeLogic DeCypher hardware (Active Motif, Inc., Carlsbad, CA). If no hit for an open reading frame-containing gene model could be found, each EST was associated with its UniGene model or listed as a singleton [88].

### Identification of differentially expressed transcripts

All available EST information for individual ESTs and library of origin were downloaded from UniGene to match paired EST reads from single clone origins. Redundantly represented clones (e.g., two or more ESTs derived from the same clone) were identified from matching clone information parsed from dbEST submission files and verified using the DotPlot (version 2.1.1) plug-in (http://sourceforge.net/projects/dotplot/) [89] for the Eclipse (version 3.4.2) software development environment (http://www.eclipse.org/) [90] with the unique name of their 8.4X gene models plotted plate-wise on two axes to verify pairs of clones. EST totals were then adjusted to reflect the correct totals [91].

The frequency of each gene in each library was calculated by dividing the EST count by library size. The EST frequencies of multiple libraries of the same type (e.g., the multiple unstressed berry libraries) were combined into a single frequency term by the weighted mean, as described by Haverty and colleagues [52]. Differences in gene expression were estimated by EST frequency for genes with at least four ESTs present in the dataset using the web tool "Identifying Differentially Expressed Genes 6" (IDEG6; (http://telethon.bio.unipd.it/bioinfo/IDEG6_form/) [53] with the recommended chi-squared test for multiple library comparisons with a p-value cut-off of < 0.0001. With these settings, IDEG6 calculates the likelihood that the frequency distribution of each gene would be expected by chance and reports the frequencies (transcripts/10,000) of genes below the cut-off. Hierarchical clustering of differentially expressed genes was performed using the Cluster software package [92], using the function (1 - Pearson correlation coefficient) as the pairwise distance metric and the average agglomeration method. The differentially expressed genes were matched to probesets found on the Affymetrix *Vitis *GeneChip^® ^microarray [55] and were then compared by Spearman rank correlation to the expression data of the significantly changed genes of multiple Affymetrix microarray experiments in which abiotic stress conditions were tested at multiple time points [10,27,31]. For the microarray probeset expression values, the time point/condition with the greatest fold-change was used for comparison and probesets with contradictory responses to stress (expression significantly increased in one condition, but significantly decreased in another) were not considered. Functional annotation was then assigned using the pathways, networks and out-of-network annotations found in *Vitis*Net software http://www.sdstate.edu/aes/vitis/pathways.cfm[55,93]. The VVM library sequences were compared to non-root EST libraries in a separate analysis, again with the IDEG6 web tool (http://telethon.bio.unipd.it/bioinfo/IDEG6_form/) [94] using the recommended Audic-Claverie (AC) statistic for comparisons of pairs, p-value < 0.01, with Bonferroni multiple-testing correction adjustment determined by the IDEG6 software (adjusted *p*-value cutoff of < 3.0 × 10^-6^) [53,60].

### Quantitative Real-time Reverse Transcriptase-PCR

Frozen leaf and shoot tissues were ground in liquid nitrogen by mortar and pestle and total RNA was extracted from the frozen powder using a Qiagen RNeasy plant mini kit (Qiagen Inc., Valencia, CA) with on-column DNase treatment according to manufacturers' instructions. Frozen berry and root tissue RNA was extracted using a Qiagen RNeasy Plant Midi kit, except that the manufacturer's instructions were modified by the addition of 2% polyethylene glycol (MW > 20,000 kD, Sigma-Aldrich, Inc., St. Louis, MO) to reduce polyphenol contamination [77]. RNA integrity was confirmed by electrophoresis on 1.5% agarose gels containing formaldehyde. cDNA was synthesized using an iScript cDNA Synthesis Kit (Bio-Rad Laboratories, Inc., Hercules, CA) according to manufacturers' instructions with a uniform 1 μg RNA/reaction volume reverse-transcribed. Gene-specific primers for real-time qRT-PCR were selected using Primer-BLAST at NCBI http://www.ncbi.nlm.nih.gov/tools/primer-blast/index.cgi?LINK_LOC=BlastHome[95] using RefSeq *V. vinifera *transcripts as input, screened against all other *V. vinifera *RefSeq sequences, and the following Primer3 [96] settings: Tm range 58-60°C, product size = 50-150 bp, primer size = 13-25 nt, max poly-X = 3, G/C content = 30-80%. Primer pairs were selected for an anti-GC clamp, such that no more than two of the last five 3' nucleotides were either G or C, as per qRT-PCR instrument recommendations. Quantitative real-time RT-PCR reactions were prepared using Fast SYBR^® ^Green Master Mix and performed using an ABI PRISM^® ^7500 Sequence Detection System (Applied Biosystems, Inc., Foster City, CA). Expression was determined for triplicate biological replicates using the ΔΔCt method, referenced to a eIF4a endogenous control gene (GSVIV gene model, GSVIVP00034135001) for leaf and berry comparisons or to an actin 7 endogenous control gene (NCBI locus ID, LOC 100232968) for shoot and root comparisons [97]. Primers designed and used in this study along with cognate gene descriptions are listed in additional files 5 and 6.

## Authors' contributions

RLT performed all EST data analyses, Perl programming, mRNA extractions, qRT-PCR, prepared all figures and tables, and wrote the initial manuscript draft. AE performed all root EST sequencing, primary data analysis and submission. RLA performed all root tissue preparations and mRNA extractions and data analyses for root cDNA library construction. KAS performed hierarchical clustering analysis and data interpretation and finalization of the manuscript. GRC participated in the organization of the studies and finalization of the manuscript. JCC conceived and organized the studies, conducted EST data analysis, tracking and submission, data interpretation, and finalized the figures and written manuscript. All authors read and approved the final manuscript.

## Supplementary Material

Additional file 1 **List of genes within the Stressed leaf cluster (SL, n = 355)**. Genes in the **SL **cluster of differentially expressed tags are listed with their *Vitis*Net-derived annotated gene description and functional category. EST frequencies (f, tags per 10,000) are shown for each library type: leaf f(L), stressed leaf f(SL), berry f(B), stressed berry f(SB). Gene IDs are for corresponding 8.4X draft genome identifiers or NCBI UniGene models. Corresponding Affymetrix *Vitis *GeneChip^® ^probeset identifiers are also shown if available. Click here for file

Additional file 2 **List of genes within the Leaf cluster (L, n = 127)**. Genes in the **L **cluster of differentially expressed tags are listed with their *Vitis*Net-derived annotated gene description and functional category. EST frequencies (f, tags per 10,000) are shown for each library type: leaf f(L), stressed leaf f(SL), berry f(B), stressed berry f(SB). Gene IDs are for corresponding 8.4X draft genome identifiers or NCBI UniGene models. Corresponding Affymetrix *Vitis *GeneChip^® ^probeset identifiers are also shown if available. Click here for file

Additional file 3 **List of genes within the Stress Berry cluster (SB, n = 127)**. Genes in the **SB **cluster of differentially expressed tags are listed with their *Vitis*Net-derived annotated gene description and functional category. EST frequencies (f, tags per 10,000) are shown for each library type: leaf f(L), stressed leaf f(SL), berry f(B), stressed berry f(SB). Gene IDs are for corresponding 8.4X draft genome identifiers or NCBI UniGene models. Corresponding Affymetrix *Vitis *GeneChip^® ^probeset identifiers are also shown if available. Click here for file

Additional file 4 **List of genes within the Berry cluster B (B, n = 130)**. Genes in the **B **cluster of differentially expressed tags are listed with their *Vitis*Net-derived annotated gene description and functional category. EST frequencies (f, tags per 10,000) are shown for each library type: leaf f(L), stressed leaf f(SL), berry f(B), stressed berry f(SB). Gene IDs are for corresponding 8.4X draft genome identifiers or NCBI UniGene models. Corresponding Affymetrix *Vitis *GeneChip^® ^probeset identifiers are also shown if available. Click here for file

Additional file 5 **List of primers used for real-time qRT-PCR of shoot and berry gene expression**. Primers were generated for real-time qRT-PCR of genes for comparison with microarray and EST frequency results. Gene name, gene model or contig identifier, forward primer (FP) and reverse primer (RP) sequences, and product size are shown. Click here for file

Additional file 6 **List of primers used for real-time qRT-PCR of root gene expression**. Primers were generated for real-time qRT-PCR corroboration of root-enriched gene expression estimated by EST frequency. Gene name, NCBI gene locus identifier, forward primer (FP) and reverse primer (RP) sequences and product size are shown. Click here for file
