# Supplementary material for: Identification of tissue-specific, abiotic stress-responsive gene expression patterns in wine grape (Vitis vinifera L.) based on curation and mining of large-scale EST data sets
Source: BMC Plant Biol. 2011 May 18;11:86. doi: 10.1186/1471-2229-11-86 (PMC3224124; doi:10.1186/1471-2229-11-86)
Supplement: Additional file 1 — List of genes within the Stressed leaf cluster (SL, n = 355). Genes in the SL cluster of differentially expressed tags are listed with their VitisNet-derived annotated gene description and functional category. EST frequencies (f, tags per 10,000) are shown for each library type: leaf f(L), stressed leaf f(SL), berry f(B), stressed berry f(SB). Gene IDs are for corresponding 8.4X draft genome identifiers or NCBI UniGene models. Corresponding Affymetrix Vitis GeneChip® probeset identifiers are also shown if available. [file 1471-2229-11-86-S1.DOC]

**Additional File 1 – List of genes within the Stressed Leaf cluster (SL, n = 355).**

**Genes in the SL cluster of differentially expressed tags are listed with their *Vitis*Net-derived annotated gene description and functional category. EST frequencies (f, tags per 10,000) are shown for each library type: leaf f(L), stressed leaf f(SL), berry f(B), stressed berry f(SB). Gene IDs are for corresponding 8.4X draft genome identifiers or NCBI UniGene models. Corresponding Affymetrix *Vitis* GeneChip® probeset identifiers are also shown if available.**

| Gene Description | Functional Category | f(L) | f(SL) | f(B) | f(SB) | Gene ID | Probeset |
| --- | --- | --- | --- | --- | --- | --- | --- |
| Glyceraldehyde-3-phosphate dehydrogenase | 1.1 Carbohydrate Metabolism | 1.9 | 8.4 | 2.4 | 1.1 | GSVIVP00002909001 | 1617428_at |
| Proline-rich family protein | Unknown | 1.9 | 7.9 | 1.2 | 1.1 | GSVIVP00002041001 | 1620660_at |
| Glutathione S-transferase 8 | 1.6 Metabolism of Other Amino Acids | 1.9 | 7.9 | 0.8 | 1.6 | GSVIVP00027957001 | 1621636_s_at |
| MPBQ/MSBQ methyltransferase 1 | 1.9 Biosynthesis of Secondary Metabolites | 1.9 | 8.8 | 1.2 | 1.6 | GSVIVP00020095001 | 1608777_at |
| FtsH protease (VAR2) | 5.3 Transport System | 5.7 | 24.2 | 0.4 | 8.1 | GSVIVP00018790001 | 1610238_s_at |
| Unknown protein | Unknown | 0 | 6.5 | 0.4 | 2.1 | GSVIVP00001172001 | 1622242_s_at |
| CYP89A2 | 1.9 Biosynthesis of Secondary Metabolites | 0 | 5.6 | 0 | 2.1 | GSVIVP00013904001 | 1614714_at |
| Ste20-related protein kinase | 3.1 Signal Transduction | 0 | 5.1 | 0 | 1.6 | GSVIVP00000530001 | 1611250_at |
| Endomembrane protein 70, TM4 | 5.3 Transport System | 0 | 6.5 | 0 | 2.1 | GSVIVP00032781001 | 1612522_at |
| Inorganic pyrophosphatase | 1.2 Energy Metabolism | 0 | 7.9 | 0.8 | 3.2 | GSVIVP00026188001 | 1612976_at |
| DnaJ homolog, subfamily B, member 4 | 5.3 Transport System | 0 | 7.4 | 0.8 | 2.1 | GSVIVP00003050001 | 1616811_at |
| Heat shock protein 101 | 5.3 Transport System | 0 | 8.4 | 1.2 | 2.1 | GSVIVP00016219001 | 1615503_at |
| Heat shock protein MTSHP | 2.3 Folding, Sorting & Degradation | 0 | 8.4 | 0.4 | 2.1 | GSVIVP00009608001 | 1620348_at |
| CP12-2 | Unclear | 1.9 | 13.5 | 3.6 | 5.8 | GSVIVP00036954001 | 1614814_s_at |
| Heat shock protein | 2.3 Folding, Sorting & Degradation | 1.9 | 28.4 | 7.2 | 6.9 | GSVIVP00018357001 | --- |
| LHCB4 chlorophyll A-B binding protein CP29 | 1.21 Photosynthesis | 0 | 15.8 | 5.1 | 3.7 | GSVIVP00021499001 | 1614598_at |
| Cellulase | 4.3 Cell Wall | 0 | 13 | 1.6 | 7.9 | GSVIVP00027662001 | --- |
| D-galactoside/L-rhamnose binding lectin | 7.0 Stress | 0 | 6.5 | 2.4 | 0 | GSVIVP00028353001 | --- |
| Periplasmic beta-glucosidase precursor | 1.9 Biosynthesis of Secondary Metabolites | 0 | 6.1 | 2.4 | 0.5 | GSVIVP00027100001 | --- |
| PSI reaction center subunit V (PSAG) | 1.21 Photosynthesis | 0 | 9.8 | 4.4 | 0.5 | GSVIVP00003325001 | 1611924_at |
| Activating signal cointegrator | 1.8 Metabolism of Cofactors & Vitamins | 0 | 5.1 | 1.2 | 0 | GSVIVP00001949001 | 1612660_at |
| Sodium- and lithium-tolerant 1 (SLT1) | 7.0 Stress | 0 | 12.6 | 2.8 | 0 | GSVIVP00024356001 | 1617276_at |
| Unknown protein | Unknown | 0 | 6.1 | 1.6 | 0 | GSVIVP00028110001 | 1618999_at |
| Unknown protein | Unknown | 0 | 4.2 | 0 | 0 | GSVIVP00025966001 | --- |
| Unknown | Unknown | 0 | 4.2 | 0 | 0 | GSVIVP00024183001 | --- |
| Unknown | Unknown | 0 | 4.2 | 0 | 0 | GSVIVP00022372001 | 1620270_at |
| Zinc finger (CCCH-type) family protein | 2.11 Transcription factor | 0 | 4.2 | 0 | 0 | GSVIVP00022218001 | 1606822_at |
| (-)-Germacrene D synthase | 1.9 Biosynthesis of Secondary Metabolites | 0 | 4.2 | 0 | 0 | GSVIVP00021747001 | --- |
| Pectinacetylesterase | 4.3 Cell Wall | 0 | 4.2 | 0 | 0 | GSVIVP00021314001 | 1608198_at |
| YGGT | Unclear | 0 | 4.2 | 0 | 0 | GSVIVP00021213001 | 1618597_at |
| Auxin-independent growth promoter | Unclear | 0 | 4.2 | 0 | 0 | GSVIVP00018300001 | 1612473_at |
| Unknown | Unknown | 0 | 4.2 | 0 | 0 | GSVIVP00017117001 | --- |
| Protein binding protein | 6.1 Binding Protein | 0 | 4.2 | 0 | 0 | GSVIVP00013780001 | 1620847_at |
| Retrotransposon gag protein | 9.0 Transposon | 0 | 4.2 | 0 | 0 | GSVIVP00012563001 | --- |
| Unknown | Unknown | 0 | 4.2 | 0 | 0 | GSVIVP00009013001 | --- |
| Miraculin precursor (MIR) | 2.3 Folding, Sorting & Degradation | 0 | 4.2 | 0 | 0 | GSVIVP00007738001 | --- |
| Ubiquitin carboxyl-terminal hydrolase L5 | 2.3 Folding, Sorting & Degradation | 0 | 4.2 | 0 | 0 | GSVIVP00005967001 | 1610904_at |
| PB1 domain-containing | Unclear | 0 | 4.2 | 0 | 0 | GSVIVP00005745001 | --- |
| Unknown | Unknown | 0 | 4.2 | 0 | 0 | GSVIVP00000752001 | --- |
| Unknown | Unknown | 0 | 4.2 | 0 | 0 | Vvi.9996 | --- |
| Unknown | Unknown | 0 | 4.2 | 0 | 0 | Vvi.9656 | --- |
| Unknown | Unknown | 0 | 4.2 | 0 | 0 | Vvi.11163 | --- |
| Unknown | Unknown | 0 | 4.2 | 0 | 0 | Vvi.11033 | --- |
| Unknown | Unknown | 0 | 4.2 | 0 | 0 | Vvi.11025 | --- |
| Unknown | Unknown | 0 | 4.2 | 0 | 0 | Vvi.10969 | --- |
| Unknown | Unknown | 0 | 4.2 | 0 | 0 | Vvi.10933 | --- |
| Unknown | Unknown | 0 | 4.2 | 0 | 0 | Vvi.10246 | --- |
| Unknown | Unknown | 0 | 8.8 | 0 | 0 | Vvi.10556 | --- |
| Unknown | Unknown | 0 | 4.2 | 0 | 0 | Vvi.10553 | --- |
| Unknown | Unknown | 0 | 6.1 | 0 | 0 | Vvi.10610 | --- |
| Unknown | Unknown | 0 | 4.2 | 0 | 0 | Vvi.10583 | --- |
| Unknown | Unknown | 0 | 6.1 | 0 | 0 | Vvi.10847 | --- |
| Unknown | Unknown | 0 | 4.2 | 0 | 0 | Vvi.10645 | --- |
| Unknown | Unknown | 0 | 6.1 | 0 | 0 | Vvi.10976 | --- |
| Unknown | Unknown | 0 | 4.2 | 0 | 0 | Vvi.10671 | --- |
| Dimethylaniline monooxygenase, N-oxide-forming | 1.10 Other | 0 | 8.8 | 0 | 0 | GSVIVP00005474001 | 1613217_at |
| Unknown | Unknown | 0 | 4.2 | 0 | 0 | Vvi.10735 | --- |
| NtPRp27 secretory protein | 7.0 Stress | 0 | 8.8 | 0 | 0 | GSVIVP00007703001 | 1617510_s_at |
| Unknown | Unknown | 0 | 4.2 | 0 | 0 | Vvi.10909 | --- |
| Unknown | Unknown | 0 | 4.2 | 0 | 0 | Vvi.10776 | --- |
| GAG-POL precursor | 9.0 Transposon | 0 | 13.1 | 0 | 0 | GSVIVP00010167001 | --- |
| Ribosomal protein S2 | 2.2 Translation | 0 | 23.3 | 0 | 0 | GSVIVP00013244001 | --- |
| Unknown | Unknown | 0 | 4.2 | 0 | 0 | Vvi.10811 | --- |
| Unknown | Unknown | 0 | 6.1 | 0 | 0 | GSVIVP00013944001 | 1617777_at |
| Unknown | Unknown | 0 | 4.2 | 0 | 0 | Vvi.10837 | --- |
| ABC transporter C member 12 | 5.3 Transport System | 0 | 6.1 | 0 | 0 | GSVIVP00034195001 | --- |
| Unknown | Unknown | 0 | 4.2 | 0 | 0 | Vvi.10841 | --- |
| Cytochrome c biogenesis orf256 | 5.3 Transport System | 0 | 6.1 | 0 | 0 | GSVIVP00035258001 | 1609672_at |
| Integrase | 9.0 Transposon | 0 | 8.4 | 0 | 0 | GSVIVP00030823001 | --- |
| Glycerophosphoryl diester phosphodiesterase | 1.3 Lipid Metabolism | 0 | 4.2 | 0 | 0 | GSVIVP00027227001 | 1614935_at |
| TCP family transcription factor TCP3 | 2.11 Transcription factor | 0 | 4.2 | 0 | 0 | GSVIVP00027783001 | 1607523_at |
| Unknown protein | Unknown | 0 | 4.2 | 0 | 0 | GSVIVP00029573001 | 1614504_s_at |
| Nitrogen fixation NifU | 1.2 Energy Metabolism | 0 | 4.2 | 0 | 0 | GSVIVP00031337001 | 1607325_at |
| Unknown | 1.5 Amino Acid Metabolism | 0 | 4.2 | 0 | 0 | GSVIVP00033302001 | --- |
| CDC48C | 4.2 Cell Growth & Death | 0 | 4.2 | 0 | 0 | GSVIVP00035083001 | 1622336_at |
| Unknown protein | Unknown | 0 | 4.2 | 0 | 0 | GSVIVP00035580001 | 1614721_at |
| Unknown | Unknown | 0 | 8.4 | 0 | 0 | GSVIVP00013509001 | 1620742_at |
| Lipoxygenase | 1.3 Lipid Metabolism | 0 | 3.7 | 0 | 0 | GSVIVP00022801001 | --- |
| ABC transporter G member 22 | 5.3 Transport System | 0 | 3.7 | 0 | 0 | GSVIVP00022041001 | 1617938_at |
| Protein kinase | 3.1 Signal Transduction | 0 | 3.7 | 0 | 0 | GSVIVP00019573001 | --- |
| Unknown | Unknown | 0 | 3.7 | 0 | 0 | GSVIVP00009445001 | --- |
| Unknown | 6.1 Binding Protein | 0 | 3.7 | 0 | 0 | GSVIVP00008930001 | --- |
| Unknown | Unknown | 0 | 3.7 | 0 | 0 | GSVIVP00008750001 | --- |
| Unknown | Unknown | 0 | 3.7 | 0 | 0 | GSVIVP00005907001 | --- |
| Unknown | Unknown | 0 | 3.7 | 0 | 0 | GSVIVP00005514001 | --- |
| Unknown | Unknown | 0 | 3.7 | 0 | 0 | Vvi.9850 | --- |
| Unknown | Unknown | 0 | 3.7 | 0 | 0 | Vvi.6880 | --- |
| Unknown | Unknown | 0 | 3.7 | 0 | 0 | Vvi.1364 | --- |
| Unknown | Unknown | 0 | 3.7 | 0 | 0 | Vvi.11118 | --- |
| Unknown | Unknown | 0 | 3.7 | 0 | 0 | Vvi.10998 | --- |
| Unknown | Unknown | 0 | 3.7 | 0 | 0 | Vvi.10983 | --- |
| Unknown | Unknown | 0 | 3.7 | 0 | 0 | Vvi.10970 | --- |
| Unknown | Unknown | 0 | 3.7 | 0 | 0 | Vvi.10963 | --- |
| Unknown | Unknown | 0 | 3.7 | 0 | 0 | Vvi.10946 | --- |
| Unknown | Unknown | 0 | 3.7 | 0 | 0 | Vvi.10939 | --- |
| Unknown | Unknown | 0 | 3.7 | 0 | 0 | Vvi.10806 | --- |
| Unknown | Unknown | 0 | 3.7 | 0 | 0 | Vvi.10545 | --- |
| Retrotransposon gag protein | 9.0 Transposon | 0 | 6 | 0 | 0 | GSVIVP00005459001 | --- |
| Unknown | Unknown | 0 | 3.7 | 0 | 0 | Vvi.10509 | --- |
| Unknown | Unknown | 0 | 6 | 0 | 0 | Vvi.11045 | --- |
| Unknown | Unknown | 0 | 3.7 | 0 | 0 | Vvi.10042 | --- |
| Unknown | Unknown | 0 | 6 | 0 | 0 | Vvi.10661 | --- |
| Unknown | Unknown | 0 | 3.7 | 0 | 0 | Vvi.10507 | --- |
| Unknown | Unknown | 0 | 6 | 0 | 0 | Vvi.10857 | --- |
| RNA-dependent RNA polymerase | 2.1 Transcription | 0 | 8.4 | 0 | 0 | GSVIVP00035561001 | 1606553_at |
| Unknown | Unknown | 0 | 3.7 | 0 | 0 | Vvi.10568 | --- |
| Unknown | Unknown | 0 | 6 | 0 | 0 | GSVIVP00021221001 | --- |
| Unknown | Unknown | 0 | 3.7 | 0 | 0 | Vvi.10640 | --- |
| Unknown | Unknown | 0 | 6 | 0 | 0 | GSVIVP00022061001 | --- |
| Unknown | Unknown | 0 | 3.7 | 0 | 0 | Vvi.10647 | --- |
| Unknown protein | Unknown | 0 | 6 | 0 | 0 | GSVIVP00030230001 | 1616568_at |
| Unknown | Unknown | 0 | 3.7 | 0 | 0 | Vvi.10677 | --- |
| Receptor protein kinase | 3.1 Signal Transduction | 0 | 6 | 0 | 0 | GSVIVP00035496001 | 1611811_at |
| Unknown | Unknown | 0 | 3.7 | 0 | 0 | GSVIVP00029468001 | 1611435_s_at |
| Unknown | Unknown | 0 | 3.7 | 0 | 0 | GSVIVP00029763001 | --- |
| Adenylate kinase | 1.4 Nucleotide Metabolism | 0 | 3.7 | 0 | 0 | GSVIVP00029879001 | 1617979_at |
| SAM:carboxyl methyltransferase | Unclear | 0 | 3.7 | 0 | 0 | GSVIVP00030607001 | 1612552_at |
| Unknown | Unknown | 0 | 3.7 | 0 | 0 | GSVIVP00033739001 | --- |
| Receptor-like kinase in flowers 1 | 3.1 Signal Transduction | 0 | 3.7 | 0 | 0 | GSVIVP00035809001 | 1621128_at |
| Subtilisin-like serine protease 2 | 2.3 Folding, Sorting & Degradation | 0 | 3.7 | 0 | 0 | GSVIVP00036104001 | 1612873_at |
| CCR4-NOT transcription complex protein | 2.11 Transcription factor | 0 | 3.7 | 0 | 0 | GSVIVP00037281001 | 1622598_at |
| MATE efflux family protein | 5.3 Transport System | 0 | 3.7 | 0 | 0 | GSVIVP00037398001 | 1618622_at |
| Unknown | Unknown | 0 | 3.7 | 0 | 0 | GSVIVP00038252001 | 1617452_at |
| Unknown | Unknown | 0 | 7.4 | 0 | 0 | Vvi.10611 | --- |
| Unknown | Unknown | 0 | 7.4 | 0 | 0 | Vvi.1604 | --- |
| Retrotransposon gag protein | 9.0 Transposon | 0 | 7.4 | 0 | 0 | GSVIVP00000833001 | --- |
| Unknown | Unknown | 0 | 7.4 | 0 | 0 | GSVIVP00023404001 | --- |
| Inducer of CBF expression 1 (ICE1) | 2.11 Transcription factor | 0 | 5.6 | 0 | 0 | GSVIVP00006182001 | 1621658_at |
| Unknown | Unknown | 0 | 5.6 | 0 | 0 | GSVIVP00003659001 | --- |
| Tetratricopeptide repeat (TPR)-containing | Unclear | 0 | 5.6 | 0 | 0 | GSVIVP00003656001 | 1614997_at |
| GTPase EngC | 3.1 Signal Transduction | 0 | 5.6 | 0 | 0 | GSVIVP00002842001 | 1610400_at |
| Nodulin family protein | 7.0 Stress | 0 | 5.6 | 0 | 0 | GSVIVP00000612001 | 1619405_s_at |
| Unknown | Unknown | 0 | 5.6 | 0 | 0 | Vvi.10858 | --- |
| Vascular plant one zinc finger protein | 2.11 Transcription factor | 0 | 7.9 | 0 | 0 | GSVIVP00003028001 | --- |
| Unknown | Unknown | 0 | 7.9 | 0 | 0 | Vvi.10758 | --- |
| Unknown | Unknown | 0 | 5.6 | 0 | 0 | Vvi.10819 | --- |
| Unknown | Unknown | 0 | 7.9 | 0 | 0 | Vvi.10659 | --- |
| Unknown | Unknown | 0 | 5.6 | 0 | 0 | Vvi.10746 | --- |
| Unknown | Unknown | 0 | 7.9 | 0 | 0 | Vvi.10558 | --- |
| Polyprotein | 9.0 Transposon | 0 | 5.6 | 0 | 0 | GSVIVP00038201001 | --- |
| Unknown | Unknown | 0 | 5.6 | 0 | 0 | Vvi.10918 | --- |
| Unknown | Unknown | 0 | 7.9 | 0 | 0 | GSVIVP00012253001 | 1607493_at |
| Unknown | Unknown | 0 | 5.6 | 0 | 0 | Vvi.11046 | --- |
| NAD(P)H dehydrogenase | 1.2 Energy Metabolism | 0 | 7.9 | 0 | 0 | GSVIVP00035660001 | 1610966_at |
| Pentatricopeptide (PPR) repeat protein | Unclear | 0 | 5.6 | 0 | 0 | GSVIVP00000138001 | 1618591_at |
| PSI P700 chlorophyll A apoprotein A1 | 1.21 Photosynthesis | 0 | 15.8 | 0 | 0 | GSVIVP00024222001 | 1613786_at |
| Unknown protein | Unknown | 0 | 5.6 | 0 | 0 | GSVIVP00030077001 | 1612337_at |
| Unknown | Unknown | 0 | 5.6 | 0 | 0 | GSVIVP00010876001 | --- |
| Prolyl endopeptidase | 2.3 Folding, Sorting & Degradation | 0 | 5.6 | 0 | 0 | GSVIVP00015534001 | 1615777_at |
| Nucleotidyltransferase | 2.1 Transcription | 0 | 5.6 | 0 | 0 | GSVIVP00017746001 | --- |
| Retrotransposon protein, Ty1-copia | 9.0 Transposon | 0 | 5.6 | 0 | 0 | GSVIVP00019821001 | --- |
| Embryo defective 1345 (emb1345) | 4.2 Cell Growth & Death | 0 | 5.6 | 0 | 0 | GSVIVP00021618001 | 1619674_at |
| Unknown | Unknown | 0 | 9.3 | 0 | 0 | GSVIVP00011374001 | --- |
| Unknown | Unknown | 0 | 9.3 | 0 | 0 | GSVIVP00011275001 | --- |
| Gag-pol polyprotein | 9.0 Transposon | 0 | 12.1 | 0 | 0 | GSVIVP00003756001 | --- |
| Unknown | Unknown | 0 | 12.1 | 0 | 0 | Vvi.9904 | --- |
| Unknown | Unknown | 0 | 10.7 | 0 | 0 | GSVIVP00026300001 | --- |
| Armadillo repeat-containing protein | Unclear | 0 | 10.7 | 0 | 0 | GSVIVP00005497001 | 1611649_at |
| Unknown | Unknown | 0 | 9.3 | 0 | 0 | GSVIVP00037421001 | --- |
| Chitinase, class IV | 1.1 Carbohydrate Metabolism | 0 | 9.3 | 0 | 0 | GSVIVP00034632001 | --- |
| Unknown protein | Unclear | 0 | 9.3 | 0 | 0 | GSVIVP00022539001 | --- |
| Unknown | Unknown | 0 | 13.5 | 0 | 0 | Vvi.10328 | --- |
| Unknown | Unknown | 0 | 13.5 | 0 | 0 | Vvi.9691 | --- |
| DEAD/DEAH box helicase (RH22) | 5.3 Transport System | 0 | 4.7 | 0 | 0 | GSVIVP00028888001 | 1609859_at |
| Unknown protein | Unknown | 0 | 4.7 | 0 | 0 | GSVIVP00025243001 | 1617816_at |
| GDP-mannose transporter | 5.3 Transport System | 0 | 4.7 | 0 | 0 | GSVIVP00024140001 | 1609106_at |
| Unknown protein | Unclear | 0 | 4.7 | 0 | 0 | GSVIVP00020494001 | 1619046_at |
| MIF4G domain-containing protein | 2.1 Transcription | 0 | 4.7 | 0 | 0 | GSVIVP00020303001 | 1616619_at |
| inorganic pyrophosphatase | 1.2 Energy Metabolism | 0 | 4.7 | 0 | 0 | GSVIVP00020253001 | --- |
| High-chlorophyll-fluorescence 101 (HCF101) | 5.3 Transport System | 0 | 4.7 | 0 | 0 | GSVIVP00016780001 | 1609667_at |
| CYP71A26 | 1.9 Biosynthesis of Secondary Metabolites | 0 | 4.7 | 0 | 0 | GSVIVP00015956001 | --- |
| Unknown | Unknown | 0 | 4.7 | 0 | 0 | GSVIVP00009666001 | --- |
| Unknown | Unknown | 0 | 4.7 | 0 | 0 | GSVIVP00008715001 | --- |
| Unknown | Unknown | 0 | 4.7 | 0 | 0 | Vvi.646 | --- |
| Unknown | Unknown | 0 | 4.7 | 0 | 0 | Vvi.11057 | --- |
| Unknown | Unknown | 0 | 4.7 | 0 | 0 | Vvi.10920 | --- |
| Unknown | Unknown | 0 | 4.7 | 0 | 0 | Vvi.10824 | --- |
| Unknown | Unknown | 0 | 4.7 | 0 | 0 | Vvi.10582 | --- |
| Unknown | Unknown | 0 | 4.7 | 0 | 0 | Vvi.10526 | --- |
| Unknown | Unknown | 0 | 4.7 | 0 | 0 | Vvi.10524 | --- |
| Unknown | Unknown | 0 | 4.7 | 0 | 0 | Vvi.10504 | --- |
| CYP86A2 | 1.9 Biosynthesis of Secondary Metabolites | 0 | 6.5 | 0 | 0 | GSVIVP00019386001 | 1618164_at |
| Unknown | Unknown | 0 | 6.5 | 0 | 0 | GSVIVP00020511001 | 1616951_at |
| Unknown | Unknown | 0 | 6.5 | 0 | 0 | GSVIVP00026528001 | --- |
| Unknown | Unknown | 0 | 6.5 | 0 | 0 | GSVIVP00029115001 | --- |
| Erythroid differentiation factor 1 | 4.2 Cell Growth & Death | 0 | 4.7 | 0 | 0 | GSVIVP00031829001 | 1609048_at |
| Unknown | Unknown | 0 | 4.7 | 0 | 0 | GSVIVP00032517001 | --- |
| Lipoxygenase | 1.3 Lipid Metabolism | 0 | 4.7 | 0 | 0 | GSVIVP00034068001 | 1617922_at |
| NADH dehydrogenase subunit 4 | 1.2 Energy Metabolism | 0 | 4.7 | 0 | 0 | GSVIVP00035614001 | 1620893_at |
| Unknown | Unknown | 0 | 6.5 | 0 | 0 | Vvi.10543 | --- |
| Unknown | Unknown | 0 | 6.5 | 0 | 0 | Vvi.10636 | --- |
| Unknown | Unknown | 0 | 6.5 | 0 | 0 | Vvi.10800 | --- |
| Unknown | Unknown | 0 | 6.5 | 0 | 0 | Vvi.10883 | --- |
| Unknown | Unknown | 0 | 6.5 | 0 | 0 | Vvi.11042 | --- |
| S-locus lectin protein kinase | 3.1 Signal Transduction | 0 | 6.5 | 0 | 0 | GSVIVP00006933001 | 1611358_at |
| D-galactoside/L-rhamnose binding SUEL lectin | 7.0 Stress | 0 | 13 | 0 | 0 | GSVIVP00006382001 | --- |
| Unknown | Unknown | 0 | 14.4 | 0 | 0 | Vvi.9836 | --- |
| Unknown | Unknown | 0 | 9.8 | 0 | 0 | GSVIVP00019082001 | --- |
| Unknown | Unknown | 0 | 5.1 | 0 | 0 | Vvi.10521 | --- |
| Unknown | Unknown | 0 | 5.1 | 0 | 0 | Vvi.10578 | --- |
| Unknown | Unknown | 0 | 5.1 | 0 | 0 | Vvi.10634 | --- |
| Unknown | Unknown | 0 | 5.1 | 0 | 0 | Vvi.10656 | --- |
| Unknown | Unknown | 0 | 5.1 | 0 | 0 | Vvi.10836 | --- |
| Unknown | Unknown | 0 | 5.1 | 0 | 0 | Vvi.10851 | --- |
| Unknown | Unknown | 0 | 5.1 | 0 | 0 | Vvi.10991 | --- |
| Unknown | Unknown | 0 | 5.1 | 0 | 0 | Vvi.9880 | --- |
| Unknown | Unknown | 0 | 5.1 | 0 | 0 | GSVIVP00005138001 | --- |
| integrase | 9.0 Transposon | 0 | 5.1 | 0 | 0 | GSVIVP00012020001 | --- |
| WAK receptor protein kinase | 3.1 Signal Transduction | 0 | 5.1 | 0 | 0 | GSVIVP00014711001 | 1612661_at |
| Chlorophyll a oxygenase (CAO) | 1.8 Metabolism of Cofactors & Vitamins | 0 | 5.1 | 0 | 0 | GSVIVP00015396001 | 1620634_at |
| Retrotransposon protein, unclassified | 9.0 Transposon | 0 | 5.1 | 0 | 0 | GSVIVP00017639001 | --- |
| Polyprotein | 9.0 Transposon | 0 | 5.1 | 0 | 0 | GSVIVP00032582001 | --- |
| Retrotransposon, centromere-specific | 9.0 Transposon | 0 | 25.6 | 0 | 0 | GSVIVP00009118001 | 1615256_at |
| Unknown | Unknown | 0 | 5.1 | 0 | 0 | GSVIVP00038608001 | 1611942_at |
| Pectinacetylesterase | 4.3 Cell Wall | 0 | 4.7 | 0.8 | 0 | GSVIVP00022503001 | 1613666_at |
| Unknown protein | Unknown | 0 | 5.1 | 0.8 | 0 | GSVIVP00030886001 | 1619937_s_at |
| Retrotransposon protein, unclassified | 9.0 Transposon | 0 | 5.6 | 0.8 | 0 | GSVIVP00035665001 | --- |
| Unknown protein | Unknown | 0 | 6.5 | 0.8 | 0 | GSVIVP00025298001 | 1608069_at |
| Unknown | Unknown | 0 | 6 | 0.8 | 0 | Vvi.7056 | --- |
| Unknown | Unknown | 0 | 12.1 | 1.6 | 0 | Vvi.7133 | --- |
| Thylakoid lumenal protein | Unclear | 0 | 4.7 | 0.4 | 0 | GSVIVP00007289001 | 1608606_at |
| Ribosomal protein L29 | 2.2 Translation | 0 | 4.7 | 0.4 | 0 | GSVIVP00016145001 | 1616941_at |
| Cycloartenol synthase | 1.3 Lipid Metabolism | 0 | 4.7 | 0.4 | 0 | GSVIVP00016985001 | 1618177_at |
| SAM decarboxylase proenzyme | 1.5 Amino Acid Metabolism | 0 | 4.7 | 0.4 | 0 | GSVIVP00017121001 | 1612003_at |
| Ubiquinol-cytochrome c reductase cyt c1 | 1.2 Energy Metabolism | 0 | 4.7 | 0.4 | 0 | GSVIVP00034552001 | 1617641_at |
| CYP72A1 | 1.9 Biosynthesis of Secondary Metabolites | 0 | 5.1 | 0.4 | 0 | GSVIVP00000208001 | 1609184_at |
| Epoxide hydrolase | 7.0 Stress | 0 | 5.1 | 0.4 | 0 | GSVIVP00037242001 | 1621789_s_at |
| Tubulin gamma Misato | 4.1 Cell Motility | 0 | 4.2 | 0.4 | 0 | GSVIVP00009705001 | 1617278_at |
| Unknown protein | Unknown | 0 | 4.2 | 0.4 | 0 | GSVIVP00014661001 | 1621735_at |
| Transaldolase | 1.1 Carbohydrate Metabolism | 0 | 4.2 | 0.4 | 0 | GSVIVP00027074001 | --- |
| Cytochrome c biogenesis protein family | 5.3 Transport System | 0 | 4.2 | 0.4 | 0 | GSVIVP00027598001 | 1610699_at |
| Knotted1-like 3 (KNAT3) | 2.11 Transcription factor | 0 | 4.2 | 0.4 | 0 | GSVIVP00032729001 | 1615625_at |
| Tetracycline transporter protein | 5.3 Transport System | 0 | 4.2 | 0.4 | 0 | GSVIVP00036173001 | 1616707_at |
| Heat shock protein 83 | 2.3 Folding, Sorting & Degradation | 0 | 4.2 | 0.4 | 0 | GSVIVP00037730001 | --- |
| Cys-3-His zinc finger protein | 2.11 Transcription factor | 0 | 7.9 | 0.8 | 0 | GSVIVP00024088001 | 1609812_at |
| Unknown protein | Unknown | 0 | 6.5 | 0.4 | 0 | GSVIVP00002447001 | 1611346_at |
| Polyprotein | 9.0 Transposon | 0 | 5.6 | 0.4 | 0 | GSVIVP00031035001 | --- |
| Proteasome 26S regulatory subunit (RPN6) | 2.3 Folding, Sorting & Degradation | 0 | 6 | 0.4 | 0 | GSVIVP00005933001 | 1607743_at |
| Unknown protein | Unknown | 0 | 6 | 0.4 | 0 | GSVIVP00016798001 | 1618905_at |
| ycf1 | Unknown | 0 | 8.4 | 0.4 | 0 | GSVIVP00012729001 | --- |
| Phosphatidylserine decarboxylase | 1.3 Lipid Metabolism | 0 | 7.4 | 0.4 | 0 | GSVIVP00031553001 | 1616691_at |
| Unknown protein | Unknown | 0 | 9.8 | 0.4 | 0 | GSVIVP00032731001 | 1619465_at |
| Unknown protein | Unknown | 0 | 9.8 | 0.4 | 0.5 | GSVIVP00007158001 | 1608348_a_at |
| Kinesin motor protein | 4.1 Cell Motility | 0 | 9.3 | 0.4 | 0.5 | GSVIVP00009412001 | --- |
| Hydroxyproline-rich glycoprotein | 4.3 Cell Wall | 0 | 24.7 | 1.2 | 1.1 | GSVIVP00027006001 | 1611683_s_at |
| Heat shock cognate 70 kDa protein 1 | 5.3 Transport System | 0 | 6.1 | 1.2 | 0.5 | GSVIVP00021301001 | 1609949_at |
| Chaperone binding | 2.3 Folding, Sorting & Degradation | 0 | 5.6 | 0.8 | 0.5 | GSVIVP00027167001 | 1609513_a_at |
| Ribosomal protein L2 50S | 2.2 Translation | 0 | 6 | 0.8 | 0.5 | GSVIVP00013198001 | --- |
| Glutathione peroxidase 4 | 1.6 Metabolism of Other Amino Acids | 0 | 6 | 0.8 | 0.5 | GSVIVP00023686001 | 1608089_at |
| Zinc finger (DNL type) | 2.11 Transcription factor | 0 | 6.1 | 0.8 | 0.5 | GSVIVP00015520001 | 1614364_at |
| Anthocyanidin reductase | 1.9 Biosynthesis of Secondary Metabolites | 0 | 7.9 | 0.8 | 0.5 | GSVIVP00005344001 | --- |
| Light harvesting complex PSII (LHCB6) | 1.21 Photosynthesis | 0 | 7.9 | 0.8 | 0.5 | GSVIVP00009554001 | 1611860_at |
| Sodium- and lithium-tolerant 1 (SLT1) | 7.0 Stress | 0 | 7.4 | 0.8 | 0.5 | GSVIVP00018505001 | 1617496_s_at |
| Splicing factor | 2.1 Transcription | 0 | 8.8 | 0.8 | 0.5 | GSVIVP00036145001 | 1610665_s_at |
| Unknown | Unknown | 0 | 4.2 | 0 | 0.5 | Vvi.10850 | --- |
| Alcohol dehydrogenase 1 | 1.1 Carbohydrate Metabolism | 0 | 4.2 | 0 | 0.5 | GSVIVP00013864001 | 1619190_at |
| VFB1 (VIER F-BOX PROTEINE 1) | 3.1 Signal Transduction | 0 | 4.2 | 0 | 0.5 | GSVIVP00014879001 | 1615580_at |
| Unknown | Unknown | 0 | 4.2 | 0 | 0.5 | GSVIVP00021483001 | 1611244_at |
| WNK3 (Arabidopsis WNK kinase 3) | 3.1 Signal Transduction | 0 | 4.2 | 0 | 0.5 | GSVIVP00027208001 | --- |
| Transposase, IS4 | 9.0 Transposon | 0 | 4.2 | 0 | 0.5 | GSVIVP00037072001 | --- |
| NAC domain containing protein 57 | 2.11 Transcription factor | 0 | 4.7 | 0 | 0.5 | GSVIVP00014676001 | 1615964_at |
| GATA transcription factor 6 | 2.11 Transcription factor | 0 | 4.7 | 0 | 0.5 | GSVIVP00020624001 | 1608686_at |
| Polyprotein | 9.0 Transposon | 0 | 10.7 | 0 | 1.1 | GSVIVP00019562001 | --- |
| GCN5 N-acetyltransferase (GNAT) | Unclear | 0 | 5.1 | 0 | 0.5 | GSVIVP00018185001 | 1613649_at |
| Glutathione S-transferase 25 GSTU7 | 1.6 Metabolism of Other Amino Acids | 0 | 5.6 | 0 | 0.5 | GSVIVP00017766001 | 1610243_at |
| SnRK2-8 | 3.2 Hormone Signaling | 0 | 6.5 | 0 | 0.5 | GSVIVP00015191001 | 1609336_s_at |
| ARP protein (REF) | Unclear | 0 | 6.5 | 0 | 0.5 | GSVIVP00036573001 | 1613655_at |
| Disease resistance protein RPS2 | 7.0 Stress | 0 | 6.1 | 0 | 0.5 | GSVIVP00035328001 | 1621775_at |
| Amidohydrolase 3 | Unclear | 0 | 7.4 | 0 | 0.5 | GSVIVP00030922001 | 1621638_at |
| Heat shock transcription factor A6B | 2.11 Transcription factor | 0 | 9.8 | 0 | 0.5 | GSVIVP00032192001 | 1610122_at |
| Galactinol synthase | 1.1 Carbohydrate Metabolism | 0 | 12.1 | 0 | 0.5 | GSVIVP00033193001 | --- |
| Hydrolase, alpha/beta fold | Unclear | 0 | 5.1 | 0.4 | 0.5 | GSVIVP00016782001 | 1621005_at |
| MLK/Raf-related protein kinase 1 | 3.1 Signal Transduction | 0 | 5.1 | 0.4 | 0.5 | GSVIVP00023765001 | 1606647_at |
| Exocyst subunit EXO70 family protein B1 | 5.3 Transport System | 0 | 5.1 | 0.4 | 0.5 | GSVIVP00024003001 | 1620717_at |
| PSII light harvesting complex gene 2.3 | 1.21 Photosynthesis | 0 | 6.5 | 0.4 | 0.5 | GSVIVP00002956001 | --- |
| Heat shock protein 16.9 kDa class I | 2.3 Folding, Sorting & Degradation | 0 | 29.8 | 1.2 | 3.2 | GSVIVP00018364001 | --- |
| Chaperone | 2.3 Folding, Sorting & Degradation | 0 | 7.9 | 0.4 | 1.1 | GSVIVP00001312001 | --- |
| Cell division protein FtsZ | 4.2 Cell Growth & Death | 0 | 8.8 | 0.4 | 1.1 | GSVIVP00024154001 | 1607332_at |
| UDP-glucose:glycoprotein glucosyltransferase | 1.1 Carbohydrate Metabolism | 0 | 8.4 | 0.4 | 1.1 | GSVIVP00026475001 | --- |
| Receptor protein kinase | 3.1 Signal Transduction | 0 | 5.1 | 0 | 1.1 | GSVIVP00021510001 | 1610478_at |
| Unknown protein | Unknown | 0 | 6.5 | 0 | 1.1 | GSVIVP00012208001 | 1617692_at |
| RNase H domain-containing protein | 2.1 Transcription | 0 | 6.1 | 0 | 1.1 | GSVIVP00032831001 | --- |
| Ubiquitin domain containing 1 | Unclear | 0 | 10.7 | 0 | 1.6 | GSVIVP00027849001 | 1608957_at |
| Myb domain protein 4 | 2.11 Transcription factor | 0 | 9.3 | 0.4 | 1.6 | GSVIVP00030469001 | 1617998_at |
| Retrotransposon protein, unclassified | 9.0 Transposon | 1.9 | 10.7 | 0.4 | 0 | GSVIVP00002653001 | 1607151_at |
| RNase H domain-containing protein | 2.1 Transcription | 1.9 | 11.6 | 0.4 | 0 | GSVIVP00016966001 | --- |
| Unknown | Unknown | 1.9 | 12.1 | 0 | 0 | Vvi.9802 | --- |
| Unknown | Unknown | 1.9 | 11.6 | 0 | 0 | GSVIVP00035663001 | --- |
| Unknown | Unknown | 1.9 | 8.4 | 0 | 0 | GSVIVP00008866001 | 1617984_at |
| Ethylene-responsive transcription factor 5 | 2.11 Transcription factor | 1.9 | 9.3 | 0 | 0 | GSVIVP00014265001 | 1619390_at |
| Cytochrome c oxidase subunit III | 1.2 Energy Metabolism | 1.9 | 10.2 | 0 | 0 | GSVIVP00035611001 | 1620991_at |
| RNA polymerase beta subunit | 2.1 Transcription | 1.9 | 10.2 | 0.8 | 0 | GSVIVP00013249001 | --- |
| Integrase core domain containing protein | 9.0 Transposon | 1.9 | 8.4 | 0.4 | 0 | GSVIVP00016976001 | --- |
| Ribosomal protein S12 30S | 2.2 Translation | 3.8 | 15.3 | 0.4 | 0 | GSVIVP00026426001 | --- |
| Auxin-binding protein ABP19a precursor | 3.2 Hormone Signaling | 3.8 | 30.7 | 4.4 | 0.5 | GSVIVP00016457001 | 1612090_s_at |
| Heat shock protein 17.4 kDa class I | 2.3 Folding, Sorting & Degradation | 7.5 | 38.6 | 5.2 | 2.1 | GSVIVP00018355001 | --- |
| ATP synthase beta chain 2, mitochondrial | 1.2 Energy Metabolism | 7.5 | 50.7 | 5.2 | 3.7 | GSVIVP00030440001 | 1614881_at |
| Leucine-rich repeat protein kinase | 3.1 Signal Transduction | 1.9 | 18.6 | 0.8 | 1.6 | GSVIVP00001442001 | 1618208_s_at |
| Photosystem I subunit O (PSAO) | 1.21 Photosynthesis | 1.9 | 15.8 | 1.2 | 1.6 | GSVIVP00037303001 | --- |
| BCL-2-associated athanogene 5 (BAG5) | 4.2 Cell Growth & Death | 1.9 | 26.1 | 0.4 | 0 | GSVIVP00006978001 | 1608429_at |
| Heat shock protein Cytosolic class II | 2.3 Folding, Sorting & Degradation | 1.9 | 28.8 | 0.4 | 1.1 | GSVIVP00032242001 | 1618391_at |
| RNA polymerase beta | 2.1 Transcription | 1.9 | 19.1 | 1.2 | 0.5 | GSVIVP00034404001 | --- |
| PSI light harvesting complex gene 2 (LHCA2) | 1.21 Photosynthesis | 9.4 | 15.8 | 5.2 | 2.6 | GSVIVP00002813001 | 1614409_at |
| Inositol-3-phosphate synthase | 1.3 Lipid Metabolism | 7.5 | 14.4 | 4.4 | 2.1 | GSVIVP00019458001 | 1613429_s_at |
| Glutamine synthetase cytosolic isozyme 1 | 1.5 Amino Acid Metabolism | 16.1 | 23.3 | 7.1 | 4.7 | GSVIVP00017890001 | 1607313_s_at |
| Lactoylglutathione lyase | 1.1 Carbohydrate Metabolism | 3.8 | 9.3 | 1.6 | 3.2 | GSVIVP00010980001 | 1609781_s_at |
| Phosphoribulokinase | 1.21 Photosynthesis | 7.5 | 14.4 | 2.4 | 4.2 | GSVIVP00014038001 | 1614716_at |
| Heat shock 22 kDa protein | 2.3 Folding, Sorting & Degradation | 5.7 | 13.1 | 3.2 | 2.6 | GSVIVP00029927001 | 1616889_at |
| Vitamin C defective (VTC2) | 1.1 Carbohydrate Metabolism | 20.7 | 52.1 | 19.9 | 15.8 | GSVIVP00027274001 | 1609268_at |
| Sedoheptulose-1,7-bisphosphatase (SBPase) | 1.21 Photosynthesis | 1.9 | 6 | 0.8 | 0.5 | GSVIVP00011932001 | 1613167_s_at |
| Peptide methionine sulfoxide reductase msrB | 1.4 Nucleotide Metabolism | 1.9 | 6.1 | 1.2 | 0.5 | GSVIVP00027935001 | 1621722_at |
| Fructose 1,6-bisphosphatase | 1.21 Photosynthesis | 1.9 | 6.5 | 0.8 | 0 | GSVIVP00021439001 | --- |
| Heat shock protein 16.9 kDa class I | 2.3 Folding, Sorting & Degradation | 9.4 | 31.2 | 5.1 | 0.5 | GSVIVP00018363001 | 1612385_at |
| Ribosomal protein S3 30S | 2.2 Translation | 5.7 | 15.3 | 3.1 | 0 | GSVIVP00013200001 | 1610728_at |
| Malate dehydrogenase [NADP] (NADP-MDH) | 1.21 Photosynthesis | 1.9 | 4.7 | 0 | 0.5 | GSVIVP00018102001 | 1611274_at |
| 4-nitrophenylphosphatase | 1.1 Carbohydrate Metabolism | 1.9 | 4.7 | 0 | 0 | GSVIVP00004877001 | 1622148_at |
| Heat shock protein 16.9 kDa class I | 2.3 Folding, Sorting & Degradation | 1.9 | 4.7 | 0 | 0 | GSVIVP00018365001 | --- |
| Tetratricopeptide helical | Unclear | 1.9 | 4.7 | 0 | 0 | GSVIVP00020351001 | --- |
| Metal ion binding | Unclear | 1.9 | 5.1 | 0 | 0 | GSVIVP00008146001 | --- |
| NADH-plastoquinone oxidoreductase subunit 5 | 1.2 Energy Metabolism | 1.9 | 5.1 | 0 | 0 | GSVIVP00012723001 | --- |
| Haloacid dehalogenase hydrolase | 1.1 Carbohydrate Metabolism | 1.9 | 4.2 | 0 | 0 | GSVIVP00015908001 | 1614290_at |
| Cytochrome b6 petB | 1.21 Photosynthesis | 1.9 | 4.2 | 0 | 0 | GSVIVP00035255001 | --- |
| Ribosomal protein S4 | 2.2 Translation | 13.2 | 30.7 | 0.4 | 0 | GSVIVP00008898001 | --- |
| Leucine-rich repeat family | 7.0 Stress | 9.4 | 22.3 | 1.6 | 0 | GSVIVP00025163001 | 1612341_s_at |
| Rubiso RBCL | 1.21 Photosynthesis | 7.5 | 17.2 | 0.8 | 0 | GSVIVP00035648001 | 1607106_s_at |
| Ycf2 | Unclear | 18.9 | 37.2 | 0.4 | 0.5 | GSVIVP00013254001 | 1610762_at |
| WRKY DNA-binding protein 33 | 2.11 Transcription factor | 3.8 | 7.9 | 0.4 | 0.5 | GSVIVP00023994001 | 1610064_at |
| Unknown protein | Unknown | 1.9 | 6.5 | 0 | 0.5 | GSVIVP00016315001 | 1608194_at |
| Thioredoxin-like protein CDSP32 | 2.3 Folding, Sorting & Degradation | 1.9 | 7.4 | 0.4 | 0.5 | GSVIVP00015512001 | 1608019_s_at |
| CYP72A1 | 1.9 Biosynthesis of Secondary Metabolites | 1.9 | 5.6 | 0.4 | 0 | GSVIVP00000202001 | 1619325_at |
| RNase H domain-containing protein | 2.1 Transcription | 3.8 | 11.2 | 0.8 | 0 | GSVIVP00009250001 | --- |
| NADH dehydrogenase subunit 4 | 1.2 Energy Metabolism | 13.2 | 38.6 | 2.4 | 0 | GSVIVP00035618001 | 1613394_at |
| Unknown | Unknown | 1.9 | 5.6 | 0 | 0 | GSVIVP00032352001 | --- |
| ATP synthase CF0 A subunit | 1.21 Photosynthesis | 32.1 | 91.2 | 2.8 | 0 | GSVIVP00013243001 | 1614676_s_at |
| Germin | 8.0 Storage | 1.9 | 6.1 | 0 | 0 | GSVIVP00000051001 | 1622369_at |
| Gag-pol polyprotein | 9.0 Transposon | 1.9 | 6.5 | 0 | 0 | GSVIVP00000297001 | --- |
| Unknown | Unknown | 3.8 | 13 | 0.4 | 0 | GSVIVP00021155001 | --- |
| ATP synthase CF1 alpha subunit | 1.21 Photosynthesis | 15.1 | 48.4 | 1.1 | 0 | GSVIVP00035586001 | 1609916_s_at |
| Mg-chelatase subunit XANTHA-F | 3.2 Hormone Signaling | 3.8 | 10.7 | 0 | 0.5 | GSVIVP00025597001 | 1607673_at |
| RNA recognition motif (RRM)-containing | Unclear | 3.8 | 5.6 | 0 | 0.5 | GSVIVP00032468001 | 1619133_at |
| NADH-plastoquinone oxidoreductase subunit I | 1.2 Energy Metabolism | 13.2 | 17.7 | 0.4 | 0 | GSVIVP00012687001 | --- |
| Unknown | Unknown | 5.7 | 9.8 | 1.1 | 0 | GSVIVP00010098001 | --- |
| 1,4-alpha-D-glucan maltohydrolase | 1.1 Carbohydrate Metabolism | 3.8 | 6.5 | 0.4 | 0 | GSVIVP00015862001 | 1616107_s_at |
| Unknown | Unknown | 7.5 | 13 | 0.8 | 0.5 | GSVIVP00035620001 | --- |
| NADH dehydrogenase subunit K | 1.2 Energy Metabolism | 49 | 74.4 | 1.6 | 0.5 | GSVIVP00013193001 | 1610039_s_at |
| Unknown protein | Unknown | 13.2 | 21.4 | 0 | 0 | GSVIVP00023592001 | --- |
| ATP synthase CF0 C chain | 1.21 Photosynthesis | 18.9 | 30.7 | 1.1 | 0 | GSVIVP00028377001 | --- |
| Clp protease proteolytic subunit | 2.3 Folding, Sorting & Degradation | 11.3 | 15.3 | 2.4 | 1.1 | GSVIVP00013213001 | --- |
| Ferredoxin:NADP+ Oxidoreductase PETH | 1.21 Photosynthesis | 7.5 | 9.8 | 0.8 | 1.1 | GSVIVP00036774001 | 1608562_at |
| Unknown | Unknown | 11.3 | 20 | 1.2 | 3.2 | GSVIVP00030259001 | 1617747_at |
| RNA polymerase beta subunit | 2.1 Transcription | 3.8 | 6.5 | 1.2 | 0 | GSVIVP00012661001 | 1622010_at |
| Membrane protein ycf1 (RF1) | Unclear | 15.1 | 25.6 | 4.4 | 0 | GSVIVP00029581001 | --- |
| Acetyl-coA carboxylase carboxyltransferase beta | 1.3 Lipid Metabolism | 11.3 | 21.9 | 3.2 | 0 | GSVIVP00026057001 | --- |
| Acetyl-CoA carboxylase beta subunit | 1.3 Lipid Metabolism | 13.2 | 25.6 | 4.4 | 0.5 | GSVIVP00035650001 | 1610156_at |
| Cytochrome f | 1.21 Photosynthesis | 11.3 | 21.9 | 5.1 | 0 | GSVIVP00035653001 | 1611247_s_at |
