# Supplementary material for: Identification of tissue-specific, abiotic stress-responsive gene expression patterns in wine grape (Vitis vinifera L.) based on curation and mining of large-scale EST data sets
Source: BMC Plant Biol. 2011 May 18;11:86. doi: 10.1186/1471-2229-11-86 (PMC3224124; doi:10.1186/1471-2229-11-86)
Supplement: Additional file 2 — List of genes within the Leaf cluster (L, n = 127). Genes in the L cluster of differentially expressed tags are listed with their VitisNet-derived annotated gene description and functional category. EST frequencies (f, tags per 10,000) are shown for each library type: leaf f(L), stressed leaf f(SL), berry f(B), stressed berry f(SB). Gene IDs are for corresponding 8.4X draft genome identifiers or NCBI UniGene models. Corresponding Affymetrix Vitis GeneChip® probeset identifiers are also shown if available. [file 1471-2229-11-86-S2.DOCX]

**Additional File 2 - List of genes within the Leaf cluster L (L, n = 127).**

Genes in the **L** cluster of differentially expressed tags are listed with their *Vitis*Net-derived annotated gene description and functional category. EST frequencies (f, tags per 10,000) are shown for each library type: leaf f(L), stressed leaf f(SL), berry f(B), stressed berry f(SB). Gene IDs are for corresponding 8.4X draft genome identifiers or NCBI UniGene models. Corresponding Affymetrix *Vitis* GeneChip^®^ probeset identifiers are also shown if available.

| Gene Description | Functional Category | f(L) | f(SL) | f(B) | f(SB) | Gene ID | probeset |
| --- | --- | --- | --- | --- | --- | --- | --- |
| Ribosomal protein L13A 60S | 2.2 Translation | 5.7 | 0 | 7.1 | 3.2 | GSVIVP00031707001 | 1612561_s_at |
| ADP-ribosylation factor A1B | 5.3 Transport System | 7.5 | 0.5 | 9.6 | 7.4 | GSVIVP00002732001 | 1616740_s_at |
| Thioredoxin H-type 1 | 2.3 Folding, Sorting and Degradation | 15.1 | 2.3 | 19.5 | 21.1 | GSVIVP00014532001 | 1614872_s_at |
| PSI reaction center subunit II | 1.21 Photosynthesis | 15.1 | 4.2 | 12.3 | 3.2 | GSVIVP00019961001 | 1611733_s_at |
| Secretion-associated RAS (SAR1) | 5.3 Transport System | 13.2 | 1.4 | 8.4 | 4.2 | GSVIVP00024546001 | --- |
| Unknown | Unknown | 7.5 | 0 | 5.2 | 0 | Vvi.8097 | --- |
| Unknown protein | Unknown | 13.2 | 0 | 8.4 | 1.6 | GSVIVP00002111001 | 1609896_at |
| Ribosomal protein L37a 60S | 2.2 Translation | 5.7 | 0 | 4.8 | 0 | GSVIVP00019030001 | 1620178_s_at |
| Unknown | Unknown | 11.3 | 0.5 | 10.8 | 0 | GSVIVP00020385001 | --- |
| Metallothionein 2b | 7.0 Stress | 11.3 | 0 | 12.3 | 0.5 | GSVIVP00024336001 | 1615445_at |
| Non-specific lipid-transfer protein | 8.0 Storage | 103.7 | 0 | 95.1 | 25.3 | GSVIVP00018836001 | 1619031_at |
| Calcium-binding protein | Unclear | 15.1 | 7.4 | 2.4 | 3.2 | GSVIVP00017815001 | 1619084_at |
| LHCB2.1 (Photosystem II) | 1.21 Photosynthesis | 13.2 | 6.5 | 2.4 | 0 | GSVIVP00029136001 | 1613691_s_at |
| Unknown | Unknown | 16.1 | 8.8 | 2.4 | 0 | GSVIVP00006895001 | --- |
| Peptidyl-prolyl cis-trans isomerase FKBP-type | 2.3 Folding, Sorting and Degradation | 5.7 | 2.8 | 0 | 0 | GSVIVP00017809001 | 1619560_at |
| Acetyl-CoA carboxylase carboxyltransferase beta | 1.3 Lipid Metabolism | 7.5 | 3.3 | 0 | 0 | GSVIVP00014289001 | --- |
| Glycine dehydrogenase | 1.5 Amino Acid Metabolism | 11.3 | 5.6 | 0.8 | 0 | GSVIVP00032211001 | 1617066_at |
| ATPase subunit 9 | 1.2 Energy Metabolism | 11.3 | 4.2 | 1.1 | 0 | GSVIVP00035666001 | 1620484_at |
| Circadian clock associated 1 (CCA1) | 2.11 Transcription factor | 15.1 | 6 | 1.6 | 1.1 | GSVIVP00026185001 | 1607032_s_at |
| Rubisco small subunit | 1.21 Photosynthesis | 550.5 | 220.5 | 15.9 | 25.3 | GSVIVP00017679001 | 1616847_s_at |
| Double strand break repair protein (XRCC4) | 2.3 Folding, Sorting and Degradation | 18.9 | 10.2 | 0.4 | 2.6 | GSVIVP00001815001 | 1621898_s_at |
| Carbonic anhydrase | 1.2 Energy Metabolism | 16.1 | 8.8 | 0.8 | 1.1 | GSVIVP00020732001 | 1613995_at |
| Unknown | Unknown | 3.8 | 4.2 | 0 | 0 | Vvi.10096 | --- |
| Galactinol synthase | 1.1 Carbohydrate Metabolism | 47.1 | 48.4 | 0.4 | 1.1 | GSVIVP00002727001 | 1617035_s_at |
| Cytochrome b6/f complex subunit IV petD | 1.21 Photosynthesis | 99.9 | 86.1 | 1.6 | 0 | GSVIVP00012660001 | 1613588_at |
| Solanesyl diphosphate synthase | 1.3 Lipid Metabolism | 5.7 | 4.2 | 0.4 | 0 | GSVIVP00027948001 | 1607360_at |
| NADH dehydrogenase subunit 2 | 1.2 Energy Metabolism | 9.4 | 6.5 | 0.8 | 0.5 | GSVIVP00014071001 | 1609373_at |
| Unknown | Unknown | 18.9 | 11.6 | 0 | 0 | GSVIVP00017889001 | --- |
| Unknown protein | Unknown | 15.1 | 11.2 | 3.6 | 2.1 | GSVIVP00026973001 | 1617840_at |
| Cytochrome B6-F complex iron-sulfur subunit, PETC | 1.21 Photosynthesis | 13.2 | 8.4 | 2.4 | 1.6 | GSVIVP00027575001 | 1620919_at |
| Photosystem II 22 kDa protein PSBS | 1.21 Photosynthesis | 20.7 | 12.6 | 2.4 | 2.6 | GSVIVP00015393001 | 1611582_s_at |
| Ribosomal protein S8 30S | 2.2 Translation | 7.5 | 6 | 1.6 | 0 | GSVIVP00013227001 | 1614503_at |
| DnaJ homolog, subfamily A, member 2 | 5.3 Transport System | 9.4 | 7.4 | 2.8 | 0 | GSVIVP00037272001 | 1619225_s_at |
| Alanine transaminase. | 1.21 Photosynthesis | 11.3 | 10.7 | 1.6 | 2.1 | GSVIVP00009478001 | 1606795_at |
| Rubisco activase | 1.21 Photosynthesis | 30.2 | 26.5 | 3.2 | 1.6 | GSVIVP00024270001 | 1616918_s_at |
| Photosystem I reaction center subunit VI PSAH | 1.21 Photosynthesis | 18.9 | 16.7 | 2.8 | 1.1 | GSVIVP00034383001 | 1611515_s_at |
| Unknown | Unknown | 16.1 | 14.9 | 3.2 | 0 | GSVIVP00032889001 | --- |
| LHCA3 (PSI light harvesting complex gene 3) | 1.21 Photosynthesis | 22.6 | 19.5 | 5.6 | 6.9 | GSVIVP00023035001 | 1613447_s_at |
| LHCII type I CAB-1 | 1.21 Photosynthesis | 15.1 | 6.1 | 5.6 | 0 | GSVIVP00027936001 | 1621038_at |
| Ferredoxin (PETF) | 1.21 Photosynthesis | 30.2 | 9.8 | 11.2 | 5.8 | GSVIVP00035477001 | 1615927_s_at |
| Pathogenesis protein 10 | 7.0 Stress | 33.9 | 7.9 | 13.5 | 6.3 | GSVIVP00033076001 | 1614464_s_at |
| Ubiquitin-conjugating enzyme E2 I | 2.3 Folding, Sorting and Degradation | 9.4 | 0.5 | 1.6 | 1.6 | GSVIVP00019543001 | 1609392_s_at |
| Transport protein particle (TRAPP) component Bet3 | 5.3 Transport System | 7.5 | 0 | 0.8 | 2.1 | GSVIVP00008897001 | 1616141_at |
| Clp protease adaptor protein | 2.3 Folding, Sorting and Degradation | 13.2 | 0.5 | 1.6 | 3.2 | GSVIVP00030302001 | 1618153_at |
| NADP-dependent D-sorbitol-6-phosphate dehydrogenase | 1.1 Carbohydrate Metabolism | 9.4 | 0 | 0.8 | 2.1 | GSVIVP00035974001 | 1612979_at |
| Unknown | Unknown | 5.7 | 0 | 1.6 | 0 | Vvi.2762 | --- |
| Unknown protein | Unknown | 13.2 | 0 | 4.4 | 1.6 | GSVIVP00032394001 | 1614627_at |
| Photosystem II PsbO protein | 1.21 Photosynthesis | 11.3 | 1.4 | 3.1 | 0 | GSVIVP00013467001 | 1622302_s_at |
| Metallothionein | 7.0 Stress | 691.9 | 68.8 | 146.2 | 43.2 | GSVIVP00027679001 | 1612971_x_at |
| Peroxiredoxin bcp | 1.2 Energy Metabolism | 5.7 | 0.9 | 0.4 | 0 | GSVIVP00016388001 | 1614204_at |
| Unknown protein | Unknown | 5.7 | 0.9 | 0.4 | 0 | GSVIVP00027365001 | 1610525_at |
| Unknown | Unknown | 15.1 | 2.3 | 1.2 | 0 | Vvi.7318 | --- |
| Glycine cleavage system H protein, mitochondrial | 1.5 Amino Acid Metabolism | 28.3 | 4.2 | 2.8 | 0.5 | GSVIVP00015781001 | 1619879_s_at |
| Photosystem II 10 kDa polypeptide PSBR | 1.21 Photosynthesis | 99.9 | 11.2 | 11.2 | 3.2 | GSVIVP00025803001 | 1618031_s_at |
| Thioredoxin M-type | 2.3 Folding, Sorting and Degradation | 11.3 | 1.4 | 0.4 | 0.5 | GSVIVP00014312001 | --- |
| Unknown | Unknown | 5.7 | 0.9 | 0 | 0 | Vvi.7252 | --- |
| WRKY DNA-binding protein 18 | 2.11 Transcription factor | 5.7 | 0.9 | 0 | 0 | GSVIVP00032689001 | 1614806_s_at |
| Photosystem II PsbK | 1.21 Photosynthesis | 90.5 | 17.2 | 3.2 | 1.1 | GSVIVP00019115001 | --- |
| Pathogenesis protein 10 | 7.0 Stress | 9.4 | 1.9 | 0.4 | 0 | GSVIVP00033078001 | --- |
| Zinc transporter ZIP5 | 5.3 Transport System | 7.5 | 1.9 | 0 | 0 | GSVIVP00037540001 | 1618682_at |
| Photosystem I P700 apoprotein A2 | 1.21 Photosynthesis | 22.6 | 6 | 0.4 | 1.1 | GSVIVP00003237001 | 1619283_s_at |
| NADH dehydrogenase subunit 7 | 1.2 Energy Metabolism | 28.3 | 7.4 | 1.1 | 1.1 | GSVIVP00006939001 | 1614348_at |
| Act domain repeat 1 (ACR1) uridylyltransferase | 2.1 Transcription | 7.5 | 2.3 | 0 | 0 | GSVIVP00025253001 | 1610649_s_at |
| Adaptor-related protein complex 4, beta 1 | 5.3 Transport System | 9.4 | 3.3 | 0.4 | 0 | GSVIVP00008592001 | --- |
| Phospholipid-hydroperoxide glutathione peroxidase. | 1.6 Metabolism of Other Amino Acids | 11.3 | 3.7 | 0.4 | 0 | GSVIVP00032791001 | 1619072_at |
| Adenosine 5' phosphosulfate reductase | 1.2 Energy Metabolism | 9.4 | 2.3 | 1.2 | 0 | GSVIVP00025889001 | 1621587_at |
| Ribosomal protein S18 30S | 2.2 Translation | 18.9 | 4.2 | 2.8 | 0 | GSVIVP00035601001 | --- |
| Catalase 3 | 3.3 Plant-Specific Signaling | 16.1 | 4.2 | 1.6 | 0 | GSVIVP00002880001 | 1610871_s_at |
| Unknown | Unknown | 32.1 | 7.4 | 3.2 | 0 | GSVIVP00006207001 | --- |
| Maturase | 2.1 Transcription | 35.8 | 8.4 | 3.6 | 0 | GSVIVP00035577001 | 1618476_s_at |
| Photosystem II oxygen-evolving complex precursor | 1.21 Photosynthesis | 20.7 | 5.6 | 3.6 | 1.1 | GSVIVP00018847001 | 1613494_s_at |
| Photosystem II PsbD | 1.21 Photosynthesis | 15.1 | 4.7 | 2.4 | 0 | GSVIVP00013233001 | 1618679_s_at |
| Photosystem I subunit XI (PSAL) | 1.21 Photosynthesis | 16.1 | 5.6 | 3.2 | 0 | GSVIVP00025097001 | 1618370_at |
| Hydroxymethylbutenyl 4-diphosphate synthase | 1.3 Lipid Metabolism | 5.7 | 0 | 0.8 | 0.5 | GSVIVP00024531001 | 1613715_at |
| Transducin family protein / WD-40 repeat | Unclear | 7.5 | 0 | 1.6 | 0.5 | GSVIVP00033452001 | 1611248_at |
| DNA-directed RNA Polymerase II subunit F | 2.1 Transcription | 5.7 | 0 | 0.8 | 0 | GSVIVP00007392001 | 1606437_at |
| ABC transporter C member 11 | 5.3 Transport System | 5.7 | 0 | 0.8 | 0 | GSVIVP00028389001 | 1612875_at |
| Zinc finger (CCCH-type) family protein | 2.11 Transcription factor | 5.7 | 0 | 0.8 | 0 | GSVIVP00028802001 | 1609344_at |
| Heat shock protein 81-1 | 2.3 Folding, Sorting and Degradation | 5.7 | 0 | 0.8 | 0 | GSVIVP00037726001 | 1618066_a_at |
| Ribosomal protein S3 30S | 2.2 Translation | 5.7 | 0 | 0.8 | 0 | GSVIVP00038203001 | 1614138_at |
| Dehydroascorbate reductase | 1.1 Carbohydrate Metabolism | 11.3 | 0 | 1.2 | 0 | GSVIVP00032988001 | 1611203_at |
| Small heat stress protein class CIII | 2.3 Folding, Sorting and Degradation | 15.1 | 0.5 | 1.6 | 0 | GSVIVP00034024001 | 1622628_at |
| Peroxisomal membrane protein | Unclear | 9.4 | 0.5 | 1.6 | 0 | GSVIVP00027620001 | 1613841_s_at |
| Aminomethyltransferase | 1.5 Amino Acid Metabolism | 7.5 | 0.5 | 0.4 | 1.1 | GSVIVP00010970001 | 1606585_at |
| Early light-inducable protein (ELIP1) | 1.2 Energy Metabolism | 435.5 | 31.2 | 9.2 | 47.1 | GSVIVP00020057001 | 1617173_s_at |
| Unknown | Unknown | 9.4 | 0.5 | 1.1 | 0.5 | Vvi.7873 | --- |
| Aspartyl protease | 2.3 Folding, Sorting and Degradation | 179.1 | 8.4 | 8.4 | 11.1 | GSVIVP00006869001 | 1611219_s_at |
| Fiber protein Fb11 | Unclear | 11.3 | 0.5 | 0.8 | 0.5 | GSVIVP00008632001 | 1613931_at |
| Quinone oxidoreductase | 1.2 Energy Metabolism | 13.2 | 0 | 0.8 | 1.1 | GSVIVP00002699001 | 1609784_s_at |
| Ribosomal protein L21 / CL21 (RPL21) 50S | 2.2 Translation | 11.3 | 0 | 0.4 | 1.1 | GSVIVP00026834001 | 1613550_at |
| Phytoene synthase | 1.9 Biosynthesis of Secondary Metabolites | 7.5 | 0 | 0 | 0.5 | GSVIVP00018857001 | 1610602_a_at |
| Glu-tRNA(Gln) amidotransferase subunit C | 1.5 Amino Acid Metabolism | 5.7 | 0 | 0 | 0.5 | GSVIVP00032138001 | 1606926_at |
| Unknown | Unknown | 32.1 | 0 | 0 | 0 | Vvi.17373 | --- |
| Unknown | Unknown | 26.4 | 0 | 0 | 0 | Vvi.17371 | --- |
| Unknown | Unknown | 7.5 | 0 | 0 | 0 | Vvi.17382 | --- |
| Unknown | Unknown | 7.5 | 0 | 0 | 0 | Vvi.17389 | --- |
| Unknown | Unknown | 26.4 | 0 | 0 | 0 | GSVIVP00011927001 | --- |
| Unknown protein | Unknown | 7.5 | 0 | 0 | 0 | GSVIVP00000876001 | 1611830_at |
| NADH dehydrogenase subunit 4L | 1.2 Energy Metabolism | 13.2 | 0 | 0 | 0 | GSVIVP00013262001 | 1614095_at |
| Unknown protein | Unknown | 7.5 | 0 | 0 | 0 | GSVIVP00018589001 | 1606572_at |
| Unknown | 1.21 Photosynthesis | 9.4 | 0 | 0 | 0 | GSVIVP00025262001 | 1614462_at |
| Unknown protein | Unknown | 49 | 0.5 | 0 | 0 | GSVIVP00029417001 | 1610934_s_at |
| Unknown | Unknown | 5.7 | 0 | 0.4 | 0 | Vvi.7323 | --- |
| Unknown | Unknown | 5.7 | 0 | 0.4 | 0 | Vvi.5783 | --- |
| Unknown | Unknown | 5.7 | 0 | 0.4 | 0 | Vvi.2444 | --- |
| F-box family protein | 2.3 Folding, Sorting and Degradation | 5.7 | 0 | 0.4 | 0 | GSVIVP00010432001 | --- |
| Unknown protein | Unknown | 5.7 | 0 | 0.4 | 0 | GSVIVP00020372001 | 1609070_a_at |
| Thioesterase family | Unclear | 5.7 | 0 | 0.4 | 0 | GSVIVP00026224001 | 1622890_at |
| Malate dehydrogenase, glyoxysomal precursor | 1.21 Photosynthesis | 5.7 | 0 | 0.4 | 0 | GSVIVP00038538001 | 1613332_at |
| Proton gradient regulation 5 (PGR5) | Unclear | 13.2 | 0 | 0.8 | 0 | GSVIVP00020332001 | 1606996_s_at |
| Myosin heavy chain | 4.1 Cell Motility | 20.7 | 0 | 1.1 | 0 | GSVIVP00024232001 | 1613733_at |
| Unknown | Unknown | 160.3 | 3.3 | 8.8 | 0 | Vvi.6907 | --- |
| Fructose-bisphosphate aldolase | 1.21 Photosynthesis | 56.6 | 0.9 | 3.2 | 0 | GSVIVP00035963001 | 1616002_s_at |
| Unknown | Unknown | 9.4 | 0 | 0.4 | 0 | GSVIVP00010999001 | --- |
| Ribosomal protein L28 (CL28) 50S | 2.2 Translation | 9.4 | 0 | 0.4 | 0 | GSVIVP00020314001 | 1607422_s_at |
| Unknown | Unknown | 11.3 | 0 | 0.4 | 0 | GSVIVP00009043001 | --- |
| Fructose-bisphosphate aldolase | 1.21 Photosynthesis | 30.2 | 0 | 1.1 | 0 | GSVIVP00036826001 | 1621944_at |
| Unknown protein | Unknown | 148.9 | 1.9 | 3.1 | 0 | GSVIVP00035259001 | --- |
| Pentatricopeptide (PPR) repeat-containing protein | Unclear | 5.7 | 0.5 | 0.4 | 0 | GSVIVP00025778001 | 1608232_at |
| Unknown | Unknown | 7.5 | 0.5 | 0.4 | 0 | Vvi.6891 | --- |
| 1-Aminocyclopropane-1-carboxylate oxidase 1 | 3.2 Hormone Signaling | 9.4 | 0.5 | 0.4 | 0 | GSVIVP00012824001 | 1615952_s_at |
| Rubisco small chain C | 1.21 Photosynthesis | 9.4 | 0.5 | 0 | 0 | GSVIVP00017680001 | --- |
| Thylakoid membrane phosphoprotein 14 kDa | Unclear | 16.1 | 0.9 | 0 | 0 | GSVIVP00026176001 | 1611476_at |
| Unknown | 2.3 Folding, Sorting and Degradation | 49 | 2.8 | 1.2 | 0 | GSVIVP00023317001 | 1607782_x_at |
| Ribosomal RNA 23S | 2.2 Translation | 173.5 | 8.4 | 2.8 | 0 | GSVIVP00035369001 | --- |
| RADIALIS-like protein 6 | 2.11 Transcription factor | 24.5 | 2.3 | 0.4 | 0 | GSVIVP00036374001 | 1610512_at |
| Cytokinin-repressed protein CR9 | 3.2 Hormone Signaling | 24.5 | 1.9 | 0 | 0 | GSVIVP00037947001 | 1616731_at |
