# Supplementary material for: Identification of tissue-specific, abiotic stress-responsive gene expression patterns in wine grape (Vitis vinifera L.) based on curation and mining of large-scale EST data sets
Source: BMC Plant Biol. 2011 May 18;11:86. doi: 10.1186/1471-2229-11-86 (PMC3224124; doi:10.1186/1471-2229-11-86)
Supplement: Additional file 3 — List of genes within the Stress Berry cluster (SB, n = 127). Genes in the SB cluster of differentially expressed tags are listed with their VitisNet-derived annotated gene description and functional category. EST frequencies (f, tags per 10,000) are shown for each library type: leaf f(L), stressed leaf f(SL), berry f(B), stressed berry f(SB). Gene IDs are for corresponding 8.4X draft genome identifiers or NCBI UniGene models. Corresponding Affymetrix Vitis GeneChip® probeset identifiers are also shown if available. [file 1471-2229-11-86-S3.DOCX]

**Additional File 3 - List of genes within the Stress Berry cluster (SB, n = 127).**

Genes in the **SB** cluster of differentially expressed tags are listed with their *Vitis*Net-derived annotated gene description and functional category. EST frequencies (f, tags per 10,000) are shown for each library type: leaf f(L), stressed leaf f(SL), berry f(B), stressed berry f(SB). Gene IDs are for corresponding 8.4X draft genome identifiers or NCBI UniGene models. Corresponding Affymetrix *Vitis* GeneChip^®^ probeset identifiers are also shown if available.

| Gene Description | Functional Category | f(L) | f(SL) | f(B) | f(SB) | Gene ID | probeset |
| --- | --- | --- | --- | --- | --- | --- | --- |
| Ribosomal protein L32 (RPL32A) 60S | 2.2 Translation | 0 | 0.5 | 7.6 | 7.4 | GSVIVP00003384001 | 1611402_at |
| Harpin-induced protein | 7.0 Stress | 0 | 0.5 | 7.2 | 6.9 | GSVIVP00021516001 | 1612504_s_at |
| Ubiquitin-conjugating enzyme 13 (UBC13), E2 | 2.3 Folding, Sorting and Degradation | 0 | 0 | 6.4 | 5.8 | GSVIVP00021300001 | 1608461_at |
| Alcohol dehydrogenase 6 | 1.1 Carbohydrate Metabolism | 0 | 0 | 9.6 | 7.9 | GSVIVP00034834001 | 1608263_a_at |
| Serine carboxypeptidase III | 2.3 Folding, Sorting and Degradation | 0 | 2.8 | 15.5 | 12.1 | GSVIVP00018113001 | 1609361_s_at |
| Unknown | Unknown | 0 | 0 | 5.1 | 6.3 | GSVIVP00003809001 | 1608122_at |
| Sucrose-phosphate synthase | 1.1 Carbohydrate Metabolism | 0 | 0 | 5.6 | 7.4 | GSVIVP00006423001 | 1615374_at |
| Expansin [*Vitis labrusca x Vitis vinifera*] EXPA8 | 3.2 Hormone Signaling | 0 | 0.5 | 14.7 | 19.5 | GSVIVP00029445001 | --- |
| Nodulin MtN3 | 7.0 Stress | 0 | 0 | 4.8 | 7.4 | GSVIVP00006971001 | 1611217_at |
| Glutathione S-transferase 25 GSTU25 | 1.6 Metabolism of Other Amino Acids | 0 | 0 | 5.1 | 7.9 | GSVIVP00012193001 | --- |
| Magnesium transporter CorA | 5.3 Transport System | 0 | 0 | 5.2 | 7.9 | GSVIVP00026430001 | 1619112_at |
| SEPALLATA3 | 2.11 Transcription factor | 0 | 0 | 6.8 | 12.1 | GSVIVP00002777001 | 1614965_at |
| Alpha-expansin 1 precursor | 3.2 Hormone Signaling | 0 | 0 | 10.8 | 18.1 | GSVIVP00015487001 | 1610418_at |
| Chitinase class IV | 1.1 Carbohydrate Metabolism | 1.9 | 0 | 21.1 | 31.1 | GSVIVP00034644001 | 1613461_s_at |
| Glutathione S-transferase 25 GSTU25 | 1.6 Metabolism of Other Amino Acids | 1.9 | 0 | 11.6 | 13.7 | GSVIVP00000218001 | 1613204_at |
| Peptidyl-prolyl cis-trans isomerase ROC5 | 2.3 Folding, Sorting and Degradation | 3.8 | 3.3 | 28.3 | 35.9 | GSVIVP00014412001 | 1615682_s_at |
| Phosphopyruvate hydratase | 1.1 Carbohydrate Metabolism | 0 | 4.2 | 22.3 | 25.8 | GSVIVP00021683001 | 1609004_s_at |
| Unknown | Unknown | 5.7 | 10.2 | 17.9 | 28.5 | GSVIVP00015547001 | 1622639_at |
| Aquaporin PIP2B | 5.3 Transport System | 3.8 | 10.2 | 23.9 | 29 | GSVIVP00024536001 | 1610982_at |
| Translationally-controlled tumor protein | Unclear | 5.7 | 13.1 | 41 | 60.1 | GSVIVP00019752001 | 1620870_s_at |
| Non-specific lipid-transfer protein | 8.0 Storage | 9.4 | 22.3 | 50.1 | 96.5 | GSVIVP00037489001 | --- |
| Iron sulfur cluster assembly protein 1 | Unclear | 9.4 | 9.3 | 3.2 | 17.4 | GSVIVP00002729001 | 1619354_s_at |
| Cysteine protease CP1 | 2.3 Folding, Sorting and Degradation | 3.8 | 7.9 | 3.6 | 15.8 | GSVIVP00014875001 | 1613772_s_at |
| Phospholipid hydroperoxide glutathione peroxidase | 1.6 Metabolism of Other Amino Acids | 3.8 | 7.4 | 3.6 | 17.9 | GSVIVP00000409001 | 1609654_at |
| Cellulase | 4.3 Cell Wall | 1.9 | 0.5 | 1.2 | 6.3 | GSVIVP00027658001 | 1611360_s_at |
| Pi starvation-induced protein | 7.0 Stress | 1.9 | 0.5 | 0.8 | 6.9 | GSVIVP00038672001 | 1622040_at |
| Thiazole biosynthetic enzyme (ARA6) | 1.8 Metabolism of Cofactors and Vitamins | 5.7 | 2.8 | 5.2 | 13.7 | GSVIVP00027385001 | 1622464_at |
| Cysteine protease | 2.3 Folding, Sorting and Degradation | 9.4 | 5.1 | 9.6 | 21.1 | GSVIVP00016442001 | 1607999_s_at |
| Small ubiquitin-like modifier 2 (SUM2) | 2.3 Folding, Sorting and Degradation | 7.5 | 0.9 | 4.8 | 12.7 | GSVIVP00025945001 | 1612973_s_at |
| Peptidyl-prolyl cis-trans isomerase ROC7 | 2.3 Folding, Sorting and Degradation | 15.1 | 2.8 | 14.3 | 27.4 | GSVIVP00024100001 | 1611204_s_at |
| Decapping 1 (DCP1) | 5.3 Transport System | 0 | 0 | 0 | 3.7 | GSVIVP00021562001 | 1618623_at |
| Cell division cycle 48-interacting UBX-domain protein | 4.2 Cell Growth and Death | 0 | 0 | 0 | 3.2 | GSVIVP00020575001 | 1613472_at |
| Seed maturation protein PM21 | 7.0 Stress | 0 | 0 | 0 | 3.2 | GSVIVP00019627001 | 1611384_at |
| APETALA2 (AP2) floral homeotic protein | 2.11 Transcription factor | 0 | 0 | 0 | 3.2 | GSVIVP00019538001 | 1607496_at |
| ABA-responsive protein HVA22F | 3.2 Hormone Signaling | 0 | 0 | 0 | 3.2 | GSVIVP00018936001 | --- |
| Elongation factor-1 alpha 3 | 2.2 Translation | 0 | 0 | 0 | 3.7 | GSVIVP00018233001 | 1620764_a_at |
| Glutathione S-transferase 25 GSTU25 | 1.6 Metabolism of Other Amino Acids | 0 | 0 | 0 | 3.2 | GSVIVP00006642001 | --- |
| Ubiquilin-1 | 2.3 Folding, Sorting and Degradation | 0 | 0 | 0 | 3.2 | GSVIVP00011747001 | 1622793_at |
| Tyrosine specific protein phosphatase | 2.3 Folding, Sorting and Degradation | 0 | 0 | 0 | 3.2 | GSVIVP00012822001 | 1613079_at |
| Unknown | Unknown | 0 | 0 | 0 | 3.7 | GSVIVP00015049001 | 1617620_at |
| Serine O-acetyltransferase (SAT-52) | 1.5 Amino Acid Metabolism | 0 | 0 | 0 | 3.7 | GSVIVP00016589001 | 1622685_at |
| Protease inhibitor/seed storage/lipid transfer protein (LTP) | 8.0 Storage | 0 | 0 | 0 | 10 | GSVIVP00020938001 | 1615158_at |
| Globulin-1 S allele precursor | 8.0 Storage | 0 | 0 | 0 | 3.2 | GSVIVP00023755001 | 1618698_at |
| Zinc finger (C3HC4-type RING finger) | 2.11 Transcription factor | 0 | 0 | 0 | 3.2 | GSVIVP00023937001 | 1606575_at |
| Universal stress protein (USP) family protein | 7.0 Stress | 0 | 0 | 0 | 3.7 | GSVIVP00027111001 | 1612110_at |
| WRKY DNA-binding protein 23 | 2.11 Transcription factor | 0 | 0 | 0 | 3.2 | GSVIVP00028138001 | 1622333_at |
| Globulin 11S | 8.0 Storage | 0 | 0 | 0 | 3.2 | GSVIVP00028589001 | 1608617_at |
| Nucleobase-ascorbate transporter 6 (NAT6) | 5.3 Transport System | 0 | 0 | 0 | 3.7 | GSVIVP00028899001 | 1611507_at |
| Jasmonate ZIM-domain protein 1 | 2.11 Transcription factor | 0 | 0 | 0 | 3.2 | GSVIVP00034049001 | 1610372_at |
| Late embryogenesis abundant protein D-34 (LEA D-34) | 7.0 Stress | 0 | 0 | 0 | 4.2 | GSVIVP00020000001 | 1611316_at |
| Wound-induced | 7.0 Stress | 0 | 0 | 0 | 4.2 | GSVIVP00029193001 | 1612664_at |
| RNA recognition motif (RRM)-containing | Unclear | 0 | 0 | 0 | 4.7 | GSVIVP00035357001 | --- |
| Hypoxia-responsive | 7.0 Stress | 0 | 0 | 0 | 4.7 | GSVIVP00029400001 | 1616127_at |
| 1-Cys peroxiredoxin | 1.5 Amino Acid Metabolism | 0 | 0 | 0 | 11.6 | GSVIVP00019700001 | 1613815_at |
| Galactosyl transferase GMA12/MNN10 | 1.7 Glycan Biosynthesis and Metabolism | 0 | 0 | 0.4 | 41.1 | GSVIVP00016294001 | 1615471_at |
| Pro-X carboxypeptidase Lysosomal | 2.3 Folding, Sorting and Degradation | 0 | 0 | 0.4 | 45.9 | GSVIVP00021554001 | 1611639_at |
| Unknown | 4.2 Cell Growth and Death | 0 | 0 | 0.4 | 20.6 | GSVIVP00025377001 | --- |
| Transcription initiation factor TFIIB | 2.1 Transcription | 0 | 0 | 0.4 | 4.7 | GSVIVP00000846001 | 1606502_at |
| 4-Coumarate-CoA ligase | 1.9 Biosynthesis of Secondary Metabolites | 0 | 0 | 0.4 | 4.2 | GSVIVP00009148001 | 1614240_at |
| GTPase activating protein | 3.1 Signal Transduction | 0 | 0 | 0.4 | 4.2 | GSVIVP00017844001 | 1607469_at |
| Splicing factor YT521-B | 2.1 Transcription | 0 | 0 | 0.4 | 4.2 | GSVIVP00025479001 | 1611553_s_at |
| Dehydration-induced protein (ERD15) | 3.2 Hormone Signaling | 0 | 0 | 0.4 | 4.2 | GSVIVP00029383001 | 1610057_at |
| Osmotin | 7.0 Stress | 0 | 0.5 | 1.6 | 14.8 | GSVIVP00001102001 | 1613811_a_at |
| Osmotin | 7.0 Stress | 0 | 0 | 0.4 | 10 | GSVIVP00001108001 | --- |
| Invertase/pectin methylesterase inhibitor | 4.3 Cell Wall | 0 | 0 | 0.8 | 20.6 | GSVIVP00015833001 | 1621352_at |
| Early nodulin 93 | 3.1 Signal Transduction | 0 | 0 | 1.1 | 19.5 | GSVIVP00016856001 | 1608410_at |
| Hydrolase, alpha/beta fold | Unclear | 0 | 0 | 0.4 | 8.1 | GSVIVP00032291001 | 1610799_at |
| Unknown | 7.0 Stress | 0 | 0 | 0.4 | 5.8 | GSVIVP00002075001 | 1620137_s_at |
| Cellulase | 4.3 Cell Wall | 0 | 0 | 0.4 | 6.3 | GSVIVP00030726001 | 1620050_at |
| Meprin and TRAFy domain-containing protein | Unclear | 0 | 0.5 | 1.2 | 21.6 | GSVIVP00020158001 | 1615839_at |
| Gamma carbonic anhydrase-like 2 | 1.2 Energy Metabolism | 0 | 0.5 | 0 | 4.2 | GSVIVP00017955001 | 1613104_at |
| Bromo-adjacenty (BAH) domain-containing protein | 2.1 Transcription | 0 | 0.5 | 0 | 5.3 | GSVIVP00020788001 | 1621434_at |
| Ankyrin repeat | 5.3 Transport System | 0 | 0.5 | 0.4 | 6.3 | GSVIVP00021308001 | 1621542_at |
| Ribosomal protein L7A (RPL7aB) 60S | 2.2 Translation | 0 | 0.5 | 0.4 | 5.8 | GSVIVP00038087001 | 1607974_at |
| Dehydrin 1b | 7.0 Stress | 0 | 1.4 | 0.8 | 13.7 | GSVIVP00036892001 | 1621592_s_at |
| DEAD box RNA helicase | 5.3 Transport System | 0 | 3.3 | 3.2 | 35.3 | GSVIVP00036305001 | 1607258_at |
| gamma-Glutamyl hydrolase precursor | 1.8 Metabolism of Cofactors and Vitamins | 0 | 0 | 0.8 | 5.3 | GSVIVP00027851001 | 1617216_s_at |
| Unknown | Unknown | 0 | 0 | 1.6 | 11.1 | Vvi.773 | --- |
| Unknown | Unknown | 0 | 0 | 1.2 | 8.4 | GSVIVP00031748001 | --- |
| Extensin | 4.3 Cell Wall | 0 | 0 | 1.2 | 7.4 | GSVIVP00030479001 | 1622641_at |
| 3-oxoacyl-reductase | 1.7 Glycan Biosynthesis and Metabolism | 0 | 0 | 2.4 | 18.5 | GSVIVP00023262001 | 1609277_at |
| Xyloglucan endotransglucosylase/hydrolase 30 | 4.3 Cell Wall | 0 | 3.7 | 1.6 | 9.5 | GSVIVP00015670001 | 1615809_at |
| Unknown | Unknown | 0 | 1.4 | 0.4 | 6.9 | GSVIVP00028083001 | --- |
| Stem-specific protein TSJT1 | Unclear | 0 | 1.4 | 0.8 | 6.3 | GSVIVP00027636001 | 1618110_s_at |
| Cofilin | 4.1 Cell Motility | 0 | 4.2 | 2.8 | 16.9 | GSVIVP00036103001 | --- |
| Late embryogenis abundant protein 5 | 7.0 Stress | 9.4 | 12.1 | 5.1 | 63.3 | GSVIVP00028026001 | 1609922_s_at |
| Haloacid dehalogenase hydrolase | 1.1 Carbohydrate Metabolism | 0 | 1.4 | 5.2 | 9.5 | GSVIVP00021335001 | 1616302_at |
| RNA-binding protein 45 (RBP45) | 5.3 Transport System | 0 | 0.5 | 4.4 | 7.9 | GSVIVP00027337001 | 1613058_a_at |
| Vacuolar processing enzyme gamma | 2.3 Folding, Sorting and Degradation | 0 | 0.9 | 5.2 | 10 | GSVIVP00032155001 | 1621718_at |
| RNA-binding protein 47B (ATRBP47B) | Unclear | 0 | 0 | 3.2 | 7.4 | GSVIVP00036552001 | 1608677_at |
| Snakin-1 | 3.2 Hormone Signaling | 0 | 0 | 29.5 | 63.8 | GSVIVP00007725001 | --- |
| Unknown | Unknown | 0 | 0 | 7.2 | 14.2 | GSVIVP00020222001 | --- |
| MADS-box protein SEEDSTICK | 2.11 Transcription factor | 0 | 0 | 5.2 | 10.5 | GSVIVP00021934001 | 1621836_at |
| Thaumatin | 7.0 Stress | 0 | 2.8 | 19.1 | 45.4 | GSVIVP00001105001 | 1620390_s_at |
| ATP citrate lyase a-subunit | 1.1 Carbohydrate Metabolism | 0 | 0 | 2.4 | 6.3 | GSVIVP00033143001 | 1622588_s_at |
| Thaumatin SCUTL2 | 7.0 Stress | 0 | 0 | 3.2 | 8.1 | GSVIVP00014680001 | 1616617_at |
| 1,4-beta-Mannan endohydrolase | 1.1 Carbohydrate Metabolism | 0 | 0 | 4.8 | 12.1 | GSVIVP00014580001 | 1612185_at |
| Major latex protein 22 | 7.0 Stress | 0 | 0 | 30.3 | 76.5 | GSVIVP00030517001 | 1617876_a_at |
| Unknown | Unknown | 0 | 0 | 50.6 | 126.6 | GSVIVP00020242001 | --- |
| Albumin, 2S | 8.0 Storage | 1.9 | 1.4 | 57.4 | 152.9 | GSVIVP00027389001 | 1615170_s_at |
| Unknown | Unknown | 0 | 0.5 | 165.7 | 492 | GSVIVP00020223001 | --- |
| Hydroperoxide lyase (HPL1) | 1.3 Lipid Metabolism | 1.9 | 0 | 4.8 | 7.9 | GSVIVP00025918001 | 1606577_at |
| Globulin isoform 3 11S | 8.0 Storage | 15.1 | 0 | 62.5 | 112.3 | GSVIVP00028585001 | 1620946_a_at |
| Ubiquitin-fold modifier 1 precursor | Unclear | 1.9 | 0 | 3.2 | 9.5 | GSVIVP00015334001 | 1610077_at |
| Proline-rich family protein | Unclear | 3.8 | 1.9 | 11.6 | 24.3 | GSVIVP00009989001 | --- |
| Calmodulin-7 (CAM7) | 3.1 Signal Transduction | 18.9 | 6.5 | 36.2 | 87 | GSVIVP00017337001 | 1610944_at |
| Fructose-bisphosphate aldolase | 1.21 Photosynthesis | 1.9 | 0 | 6.8 | 16.3 | GSVIVP00030426001 | 1620673_at |
| Heat shock cognate 70 kDa protein 1 | 5.3 Transport System | 0 | 2.3 | 2.4 | 8.1 | GSVIVP00024351001 | 1616246_at |
| Unknown | Unknown | 1.9 | 3.3 | 5.2 | 16.9 | GSVIVP00001486001 | 1613847_s_at |
| Cupin | 8.0 Storage | 0 | 0.9 | 1.6 | 7.4 | GSVIVP00026516001 | 1607320_s_at |
| Fatty acid multifunctional protein (MFP2) | 1.3 Lipid Metabolism | 0 | 0.9 | 1.6 | 7.4 | GSVIVP00033022001 | 1616386_s_at |
| Sucrose synthase | 1.1 Carbohydrate Metabolism | 0 | 0.9 | 3.2 | 12.7 | GSVIVP00016378001 | 1619223_s_at |
| Unknown | Unknown | 0 | 2.3 | 6.8 | 32.2 | GSVIVP00027237001 | --- |
| Inorganic pyrophosphatase | 1.2 Energy Metabolism | 0 | 1.9 | 4.4 | 15.8 | GSVIVP00024902001 | 1609853_at |
| Thaumatin VVTL1 | 7.0 Stress | 0 | 1.4 | 15.9 | 54.3 | GSVIVP00001103001 | --- |
| Unknown | Unknown | 0 | 0 | 8.8 | 34.3 | GSVIVP00020240001 | --- |
| Xyloglucan endotransglucosylase/hydrolase 32 | 4.3 Cell Wall | 0 | 0 | 15.9 | 59.6 | GSVIVP00022167001 | 1621251_s_at |
| Aspartyl protease | 2.3 Folding, Sorting and Degradation | 0 | 0 | 11.9 | 43.8 | GSVIVP00027239001 | 1606471_at |
| Eukaryotic translation initiation factor 5A isoform II | 2.2 Translation | 1.9 | 0.9 | 3.1 | 14.2 | GSVIVP00004748001 | 1611557_s_at |
| Globulin-1 S allele precursor | 8.0 Storage | 5.7 | 0 | 16.3 | 58.5 | GSVIVP00023754001 | --- |
| Acyl-CoA binding protein | Unclear | 1.9 | 25.1 | 13.9 | 21.6 | GSVIVP00003181001 | 1609433_s_at |
| Heat shock cognate 70 kDa protein 1 | 5.3 Transport System | 9.4 | 21.9 | 5.6 | 17.9 | GSVIVP00018481001 | 1613630_s_at |
| RAB GTPase RAB1C | 3.1 Signal Transduction | 0 | 9.3 | 1.6 | 8.4 | GSVIVP00014195001 | 1606813_s_at |
| Unknown | Unknown | 0 | 12.1 | 1.1 | 13.2 | Vvi.7561 | --- |
| Glycine-rich RNA-binding protein 3 (GR-RBP3) | 7.0 Stress | 1.9 | 9.8 | 1.2 | 14.2 | GSVIVP00016201001 | 1613722_s_at |
| CBL-interacting protein kinase 10 (CIPK10) | 3.1 Signal Transduction | 5.7 | 32.6 | 1.6 | 47.1 | GSVIVP00023961001 | 1615895_at |
| RD22 | 7.0 Stress | 5.7 | 38.6 | 11.6 | 49 | GSVIVP00032481001 | 1616426_at |
