# Supplementary material for: Identification of tissue-specific, abiotic stress-responsive gene expression patterns in wine grape (Vitis vinifera L.) based on curation and mining of large-scale EST data sets
Source: BMC Plant Biol. 2011 May 18;11:86. doi: 10.1186/1471-2229-11-86 (PMC3224124; doi:10.1186/1471-2229-11-86)
Supplement: Additional file 4 — List of genes within the Berry cluster B (B, n = 130). Genes in the B cluster of differentially expressed tags are listed with their VitisNet-derived annotated gene description and functional category. EST frequencies (f, tags per 10,000) are shown for each library type: leaf f(L), stressed leaf f(SL), berry f(B), stressed berry f(SB). Gene IDs are for corresponding 8.4X draft genome identifiers or NCBI UniGene models. Corresponding Affymetrix Vitis GeneChip® probeset identifiers are also shown if available. [file 1471-2229-11-86-S4.DOCX]

**Additional File 4 - List of genes within the Berry cluster B (B, n = 130).**

Genes in the **B** cluster of differentially expressed tags are listed with their *Vitis*Net-derived annotated gene description and functional category. EST frequencies (f, tags per 10,000) are shown for each library type: leaf f(L), stressed leaf f(SL), berry f(B), stressed berry f(SB). Gene IDs are for corresponding 8.4X draft genome identifiers or NCBI UniGene models. Corresponding Affymetrix *Vitis* GeneChip^®^ probeset identifiers are also shown if available.

| Gene Description | Functional Category | f(L) | f(SL) | f(B) | f(SB) | Gene ID | probeset |
| --- | --- | --- | --- | --- | --- | --- | --- |
| Isopiperitenol dehydrogenase | 1.9 Biosynthesis of Secondary Metabolites | 0 | 0 | 4.4 | 0 | GSVIVP00000079001 | 1618595_at |
| Aminopeptidase N | 1.6 Metabolism of Other Amino Acids | 0 | 0 | 3.6 | 0 | GSVIVP00000444001 | 1615015_at |
| Ubiquinol-cytochrome C reductase iron-sulfur subunit | 1.2 Energy Metabolism | 0 | 0 | 3.6 | 0 | GSVIVP00006205001 | 1609752_at |
| Diacylglycerol kinase | 3.1 Signal Transduction | 0 | 0 | 3.6 | 0 | GSVIVP00007033001 | 1614177_at |
| Protease inhibitor/seed storage/lipid transfer protein (LTP) | 8.0 Storage | 0 | 0 | 3.6 | 0 | GSVIVP00007385001 | --- |
| Cytochrome b5 domain-containing protein | Unclear | 0 | 0 | 3.6 | 0 | GSVIVP00009206001 | 1606958_s_at |
| Histone H2B | 2.4 Replication and Repair | 0 | 0 | 3.6 | 0 | GSVIVP00018093001 | 1622737_at |
| Unknown | Unknown | 0 | 0 | 3.6 | 0 | GSVIVP00018921001 | 1613074_at |
| Histone deacetylase (HD2A) | 4.2 Cell Growth and Death | 0 | 0 | 3.1 | 0 | GSVIVP00022095001 | 1612053_at |
| Unknown protein | Unknown | 0 | 0 | 3.1 | 0 | GSVIVP00023194001 | 1617055_at |
| Tropinone reductase | 1.5 Amino Acid Metabolism | 0 | 0 | 3.1 | 0 | GSVIVP00024239001 | 1617214_s_at |
| Nitrogen regulatory protein PII 1 | 5.3 Transport System | 0 | 0 | 3.6 | 0 | GSVIVP00028171001 | 1607955_at |
| Poly(ADP-ribose) like glycohydrolase | Unclear | 0 | 0 | 4.4 | 0 | GSVIVP00034768001 | 1613106_at |
| Invertase/pectin methylesterase inhibitor | 4.3 Cell Wall | 0 | 0 | 4.4 | 0 | GSVIVP00035726001 | 1609651_at |
| Unknown | Unknown | 0 | 0 | 5.2 | 0 | GSVIVP00001230001 | 1607851_at |
| Invertase/pectin methylesterase inhibitor | 4.3 Cell Wall | 0 | 0 | 5.2 | 0 | GSVIVP00016390001 | 1622535_at |
| Ribosomal protein S28 (RPS28C) 40S | 2.2 Translation | 0 | 0 | 5.6 | 0 | GSVIVP00025456001 | 1622158_s_at |
| Lipase GDSL | 1.3 Lipid Metabolism | 0 | 0 | 5.2 | 0 | GSVIVP00026719001 | 1615130_at |
| Xyloglucan endotransglucosylase/hydrolase 23 | 4.3 Cell Wall | 0 | 0 | 5.2 | 0 | GSVIVP00031405001 | 1610073_at |
| Adenosylhomocysteinase | 1.6 Metabolism of Other Amino Acids | 0 | 0 | 5.2 | 0 | GSVIVP00031656001 | 1619097_a_at |
| LHCII type I CAB-1 | 1.21 Photosynthesis | 0 | 0 | 5.1 | 0 | GSVIVP00003413001 | 1618116_s_at |
| Eukaryotic translation initiation factor 5A isoform II | 2.2 Translation | 0 | 0 | 6.8 | 0 | GSVIVP00016285001 | 1613385_s_at |
| Tubulin beta-4 chain | 4.1 Cell Motility | 0 | 0 | 4.8 | 0 | GSVIVP00017326001 | 1616815_at |
| Epicotyl-specific tissue protein | Unclear | 0 | 0 | 6.8 | 0 | GSVIVP00036409001 | 1612030_at |
| Proteinase inhibitor se60 | 2.3 Folding, Sorting and Degradation | 0 | 0 | 14.3 | 0 | GSVIVP00003667001 | 1611222_at |
| Heat shock protein 17.4 kDa class I | 2.3 Folding, Sorting and Degradation | 0 | 0 | 7.6 | 0 | GSVIVP00024197001 | 1621652_at |
| Cationic peroxidase | 1.5 Amino Acid Metabolism | 0 | 0 | 6.4 | 0 | GSVIVP00010753001 | --- |
| Chitinase class IV | 1.1 Carbohydrate Metabolism | 0 | 0 | 15.5 | 0.5 | GSVIVP00034646001 | 1621319_s_at |
| Unknown | Unknown | 0 | 0.5 | 18.3 | 0 | Vvi.5308 | --- |
| Bet v I allergen | 7.0 Stress | 0 | 0.9 | 17.1 | 0 | GSVIVP00028882001 | 1610299_at |
| Metallothionein | 7.0 Stress | 1.9 | 3.7 | 64.1 | 1.1 | GSVIVP00021317001 | 1607049_s_at |
| Hydrolase, alpha/beta fold | Unclear | 0 | 0.5 | 4.8 | 0 | GSVIVP00016338001 | 1609457_at |
| Unknown | Unknown | 0 | 0.5 | 5.6 | 0 | Vvi.268 | --- |
| Unknown | Unknown | 0 | 0.9 | 7.2 | 0 | GSVIVP00038158001 | 1622194_at |
| Stress-induced | 7.0 Stress | 0 | 1.4 | 11.9 | 0 | GSVIVP00038592001 | 1619147_at |
| Superoxide dismutase | 3.1 Signal Transduction | 0 | 0.9 | 8.8 | 0.5 | GSVIVP00008877001 | 1616657_at |
| DNA-binding protein | 2.4 Replication and Repair | 1.9 | 0 | 17.1 | 0.5 | GSVIVP00020747001 | 1609471_s_at |
| Proteasome 20S beta subunit F1 (PBF1) | 2.3 Folding, Sorting and Degradation | 3.8 | 0 | 39.4 | 2.6 | GSVIVP00026350001 | 1607436_at |
| Ribosomal protein P2 (RPP2A) acidic 60S | 2.2 Translation | 0 | 1.4 | 7.6 | 1.6 | GSVIVP00021645001 | 1612384_s_at |
| Ribosomal protein S2 (RPS2D) 40S | 2.2 Translation | 0 | 0.9 | 7.1 | 2.1 | GSVIVP00025575001 | 1622564_at |
| Zinc finger (CCCH-type) family protein | 2.11 Transcription factor | 0 | 0.5 | 5.6 | 1.1 | GSVIVP00025561001 | 1611185_at |
| Ribosomal protein L11 (RPL11D) 60S | 2.2 Translation | 0 | 0.9 | 7.6 | 1.6 | GSVIVP00035844001 | 1613245_s_at |
| Ribosomal protein S27 (ARS27A) 40S | 2.2 Translation | 0 | 2.3 | 17.1 | 4.2 | GSVIVP00006221001 | 1620069_s_at |
| Pectate lyase | 4.3 Cell Wall | 0 | 0.9 | 10.4 | 2.6 | GSVIVP00020459001 | 1618576_at |
| Protein transport protein SEC61 gamma subunit | 5.3 Transport System | 0 | 0.5 | 7.2 | 1.1 | GSVIVP00037864001 | 1621995_at |
| Ribosomal protein S16 (RPS16A) 40S | 2.2 Translation | 0 | 0.9 | 6.8 | 1.1 | GSVIVP00036621001 | 1610092_at |
| Pollen Ole e 1 allergen and extensin | 4.3 Cell Wall | 0 | 0.9 | 7.1 | 1.1 | GSVIVP00011900001 | 1622009_at |
| Ribosomal protein L38 (RPL38B) 60S | 2.2 Translation | 0 | 0.9 | 8.8 | 1.1 | GSVIVP00010306001 | 1612270_s_at |
| Pollen Ole e 1 allergen and extensin | 4.3 Cell Wall | 0 | 1.4 | 10.4 | 1.1 | GSVIVP00015265001 | 1608493_at |
| Ribosomal protein P3 (RPP3B) acidic 60S | 2.2 Translation | 0 | 0 | 5.6 | 1.1 | GSVIVP00001545001 | 1618101_at |
| Basic helix-loop-helix ILR3 | 2.11 Transcription factor | 0 | 0 | 5.6 | 1.1 | GSVIVP00027432001 | 1610421_at |
| CYP78A4 | 1.9 Biosynthesis of Secondary Metabolites | 0 | 0.5 | 13.1 | 2.6 | GSVIVP00000025001 | 1607719_at |
| Unknown protein | Unknown | 0 | 0.5 | 15.5 | 3.2 | GSVIVP00032599001 | 1614520_s_at |
| Endochitinase C, basic | 1.1 Carbohydrate Metabolism | 9.4 | 0 | 142.6 | 29.5 | GSVIVP00011591001 | --- |
| Ribosomal protein L24 60S | 2.2 Translation | 0 | 0 | 4.8 | 0.5 | GSVIVP00010804001 | 1607953_at |
| Hydrogenobyrinic acid a,c-diamide synthase | 5.3 Transport System | 0 | 0 | 5.1 | 0.5 | GSVIVP00019628001 | 1619687_at |
| Chalcone synthase | 1.9 Biosynthesis of Secondary Metabolites | 0 | 0 | 5.6 | 0.5 | GSVIVP00006341001 | 1606663_at |
| Indole-3-acetate beta-glucosyltransferase | 1.9X hormone biosynth | 0 | 0 | 4.4 | 0.5 | GSVIVP00024021001 | 1620575_at |
| Ripening-related protein | Unclear | 0 | 0 | 18.3 | 2.1 | GSVIVP00010880001 | 1622854_s_at |
| Ribosomal protein P1 (RPP1B) acidic 60S | 2.2 Translation | 0 | 0 | 8.4 | 1.1 | GSVIVP00037178001 | 1622420_at |
| Bet v I allergen | 7.0 Stress | 0 | 0.5 | 13.9 | 1.1 | GSVIVP00030529001 | 1612444_at |
| Ribosomal protein S9 (RPS9C) 40S | 2.2 Translation | 1.9 | 2.3 | 9.6 | 2.6 | GSVIVP00000124001 | 1610517_s_at |
| Unknown protein | 4.2 Cell Growth and Death | 1.9 | 0.5 | 8.4 | 2.6 | GSVIVP00026965001 | 1614614_at |
| Ribosomal protein L26 (RPL26A) 60S | 2.2 Translation | 1.9 | 0 | 6.4 | 2.1 | GSVIVP00038278001 | 1608425_at |
| Ribosomal protein S21 (RPS21C) 40S | 2.2 Translation | 1.9 | 1.4 | 11.2 | 3.2 | GSVIVP00019777001 | 1610997_at |
| DRM1 dormancy/auxin associated | 3.2 Hormone Signaling | 1.9 | 0.5 | 11.2 | 2.6 | GSVIVP00003066001 | 1607503_s_at |
| Cyclase | Unclear | 3.8 | 1.9 | 28.7 | 7.9 | GSVIVP00036644001 | 1618366_a_at |
| Elongation factor 1-beta 1 | 2.2 Translation | 1.9 | 0 | 5.6 | 0.5 | GSVIVP00017287001 | 1620700_at |
| Triosephosphate isomerase, cytosolic | 1.21 Photosynthesis | 1.9 | 0 | 5.2 | 0.5 | GSVIVP00018496001 | 1610469_at |
| DnaJ homolog, subfamily A, member 2 | 5.3 Transport System | 5.7 | 0 | 16.3 | 0.5 | GSVIVP00002640001 | 1611047_at |
| Omega-6 fatty acid desaturase (FAD2) | 1.7 Glycan Biosynthesis and Metabolism | 1.9 | 0 | 6.4 | 0.5 | GSVIVP00024716001 | 1617693_at |
| Transaldolase | 1.1 Carbohydrate Metabolism | 1.9 | 0 | 7.2 | 0.5 | GSVIVP00007295001 | --- |
| Histone H3 | 2.4 Replication and Repair | 1.9 | 0 | 8.4 | 0 | GSVIVP00008852001 | 1612573_at |
| Ras GTP-binding protein (RAN3) | 5.3 Transport System | 1.9 | 0.9 | 6.8 | 1.1 | GSVIVP00032600001 | 1607281_at |
| Aquaporin TMP-C | 5.3 Transport System | 9.4 | 6 | 45 | 5.3 | GSVIVP00000433001 | 1615517_x_at |
| Protease inhibitor/seed storage/lipid transfer protein (LTP) | 8.0 Storage | 3.8 | 0.5 | 14.7 | 2.1 | GSVIVP00009621001 | 1619613_at |
| Histone H4 | 2.4 Replication and Repair | 3.8 | 0.9 | 15.5 | 2.6 | GSVIVP00018516001 | 1613076_at |
| Nodulin MtN3 family | 7.0 Stress | 1.9 | 0 | 8.8 | 1.6 | GSVIVP00017991001 | 1615375_at |
| GRP7 (CCR2) | 5.3 Transport System | 3.8 | 1.4 | 9.6 | 2.1 | GSVIVP00036381001 | 1616828_s_at |
| Spiral 1 like 2 | 4.1 Cell Motility | 3.8 | 0.9 | 8.4 | 2.1 | GSVIVP00038038001 | 1618440_s_at |
| Ubiquitin-conjugating enzyme E2 D/E | 2.3 Folding, Sorting and Degradation | 5.7 | 1.4 | 12.7 | 3.7 | GSVIVP00002469001 | 1618769_at |
| Ketol-acid reductoisomerase | 1.8 Metabolism of Cofactors and Vitamins | 3.8 | 0.9 | 8.4 | 1.1 | GSVIVP00018719001 | 1615699_s_at |
| Ribosomal protein S11 (RPS11C) 40S | 2.2 Translation | 3.8 | 1.4 | 9.2 | 1.1 | GSVIVP00032736001 | 1610354_s_at |
| Fructose-bisphosphate aldolase cytoplasmic isozyme | 1.21 Photosynthesis | 11.3 | 5.1 | 21.9 | 7.4 | GSVIVP00021665001 | 1608614_at |
| Metallothionein | 7.0 Stress | 28.3 | 6 | 57.4 | 21.6 | GSVIVP00024335001 | --- |
| Unknown protein | Unknown | 5.7 | 0 | 10.8 | 0 | GSVIVP00027651001 | 1610720_at |
| Ribosomal protein L27 (RPL27C) 60S | 2.2 Translation | 3.8 | 0 | 7.1 | 0.5 | GSVIVP00030172001 | 1610389_s_at |
| Aquaporin DELTA-TIP | 5.3 Transport System | 3.8 | 0 | 9.1 | 0 | GSVIVP00034350001 | 1610955_s_at |
| Glycine-rich protein | Unclear | 11.3 | 3.7 | 21.9 | 0 | GSVIVP00036072001 | 1619498_a_at |
| Unknown protein | Unknown | 0 | 5.1 | 9.2 | 0.5 | GSVIVP00012867001 | 1620349_at |
| RD22 | 7.0 Stress | 0 | 3.3 | 10.8 | 0 | GSVIVP00032485001 | 1621818_at |
| LHCII type I CAB-1 | 1.21 Photosynthesis | 7.5 | 9.8 | 26.7 | 1.1 | GSVIVP00003412001 | 1612273_at |
| Peroxiredoxin-5 | 1.2 Energy Metabolism | 3.8 | 4.7 | 16.7 | 12.1 | GSVIVP00016713001 | 1620288_s_at |
| Aquaporin PIP3 | 5.3 Transport System | 1.9 | 1.9 | 11.6 | 9.5 | GSVIVP00036133001 | 1612244_s_at |
| Related to Ubiquitin 1 (RUB1) | 3.2 Hormone Signaling | 16.1 | 9.3 | 35.8 | 28.5 | GSVIVP00035709001 | 1617504_s_at |
| Ribosomal protein S15 (RPS15D) 40S | 2.2 Translation | 3.8 | 0.5 | 9.1 | 10 | GSVIVP00020133001 | 1616502_at |
| Abscisic stress ripening protein 2 (ASR2) | 7.0 Stress | 54.7 | 21.4 | 151.8 | 152.4 | GSVIVP00031749001 | 1616455_s_at |
| Actin 7 (ACT7) / actin 2 | 4.1 Cell Motility | 3.8 | 5.1 | 16.7 | 8.1 | GSVIVP00034893001 | 1606368_s_at |
| Glyceraldehyde-3-phosphate dehydrogenase | 1.1 Carbohydrate Metabolism | 3.8 | 6.5 | 21.5 | 11.6 | GSVIVP00009717001 | 1608869_s_at |
| Elongation factor 1-alpha 1 | 2.2 Translation | 7.5 | 28.8 | 62.5 | 46.4 | GSVIVP00024491001 | 1615105_s_at |
| Ribosomal protein S29 (RPS29C) 40S | 2.2 Translation | 0 | 2.8 | 11.2 | 6.3 | GSVIVP00016417001 | 1608093_at |
| Ribosomal protein S25 (RPS25E) 40S | 2.2 Translation | 0 | 2.3 | 11.6 | 4.2 | GSVIVP00007016001 | 1616442_a_at |
| Gibberellin-regulated protein 1 (GASA1) | 3.2 Hormone Signaling | 0 | 1.4 | 10.8 | 4.7 | GSVIVP00014423001 | 1609893_at |
| Acyl carrier protein 2 (ACP2) | 5.3 Transport System | 0 | 0 | 6.4 | 2.1 | GSVIVP00008887001 | 1606532_s_at |
| PQ-loop repeat protein | Unclear | 0 | 0 | 6.4 | 2.1 | GSVIVP00025009001 | 1611561_a_at |
| Ribosomal protein S17 (RPS17A) 40S | 2.2 Translation | 0 | 0 | 7.6 | 2.6 | GSVIVP00013816001 | 1618317_at |
| SHATTERPROOF 2 | 2.11 Transcription factor | 0 | 0 | 6.8 | 2.6 | GSVIVP00018932001 | 1619742_at |
| Polygalacturonase inhibitor protein | 7.0 Stress | 0 | 0 | 7.1 | 2.6 | GSVIVP00024648001 | 1610756_at |
| Allergenic protein Pt2L4 | 7.0 Stress | 0 | 0 | 11.6 | 4.2 | GSVIVP00025871001 | 1615272_at |
| Pathogenesis-related protein-4 (Chitinase) | 1.1 Carbohydrate Metabolism | 0 | 0 | 7.2 | 2.1 | GSVIVP00007453001 | 1618835_s_at |
| Aquaporin PIP2;4 | 5.3 Transport System | 0 | 0 | 9.2 | 2.6 | GSVIVP00023192001 | 1615415_s_at |
| CYCP4;1 (cyclin p4;1) | 4.2 Cell Growth and Death | 0 | 0.5 | 8.8 | 3.7 | GSVIVP00022839001 | --- |
| Thioredoxin H | 2.3 Folding, Sorting and Degradation | 0 | 0.5 | 8.4 | 3.2 | GSVIVP00036859001 | 1618911_at |
| Invertase/pectin methylesterase inhibitor | 4.3 Cell Wall | 0 | 0 | 52.1 | 22.7 | GSVIVP00029882001 | 1615974_at |
| Non-specific lipid transfer protein LTP1 | 3.1 Signal Transduction | 0 | 0 | 19.1 | 8.1 | GSVIVP00037486001 | 1622416_at |
| Elongation factor EF-2 | 2.2 Translation | 0 | 0.9 | 8.8 | 4.7 | GSVIVP00025739001 | 1615787_at |
| GDP-mannose 3,5-epimerase 1 | 1.1 Carbohydrate Metabolism | 0 | 0.5 | 7.6 | 4.2 | GSVIVP00020104001 | 1607874_at |
| Unknown protein | Unknown | 0 | 0.5 | 15.1 | 8.4 | GSVIVP00028751001 | 1613834_at |
| Unknown | Unknown | 0 | 2.8 | 56.2 | 28.5 | GSVIVP00027242001 | 1618589_s_at |
| Glucan endo-1,3-beta-glucosidase 3 precursor | 1.1 Carbohydrate Metabolism | 0 | 0 | 5.1 | 2.6 | GSVIVP00016211001 | 1616192_at |
| Kiwellin ripening-related protein grip22 | 7.0 Stress | 1.9 | 0 | 55.4 | 30.1 | GSVIVP00024571001 | 1615212_at |
| Vacuolar invertase 1, GIN1 | 1.1 Carbohydrate Metabolism | 0 | 0 | 7.2 | 4.2 | GSVIVP00029907001 | 1611613_at |
| Unknown | Unknown | 0 | 0 | 10.8 | 6.3 | GSVIVP00031070001 | 1614329_s_at |
| Unknown | Unknown | 0 | 0 | 27.9 | 16.3 | GSVIVP00027235001 | --- |
| DNA-damage-repair/toleration protein (DRT100) | 2.4 Replication and Repair | 0 | 0 | 11.2 | 7.4 | GSVIVP00008840001 | 1619667_at |
| Gamma-thionin precursor | 2.3 Folding, Sorting and Degradation | 0 | 0.5 | 9.1 | 6.3 | GSVIVP00035146001 | 1608412_at |
| Ribosomal protein L19 (RPL19B) 60S | 2.2 Translation | 0 | 0.9 | 19.5 | 14.2 | GSVIVP00028043001 | 1621149_at |
| Cofilin | 4.1 Cell Motility | 1.9 | 0 | 9.1 | 4.7 | GSVIVP00024377001 | 1606442_at |
| S-adenosylmethionine synthetase 1 (SAM1) | 1.6 Metabolism of Other Amino Acids | 1.9 | 0 | 14.3 | 7.4 | GSVIVP00019707001 | 1614282_s_at |
| 7S globulin precursor, basic | 8.0 Storage | 3.8 | 0 | 31.5 | 20 | GSVIVP00022863001 | 1614711_at |
