# Supplementary material for: Identification of tissue-specific, abiotic stress-responsive gene expression patterns in wine grape (Vitis vinifera L.) based on curation and mining of large-scale EST data sets
Source: BMC Plant Biol. 2011 May 18;11:86. doi: 10.1186/1471-2229-11-86 (PMC3224124; doi:10.1186/1471-2229-11-86)
Supplement: Additional file 5 — List of primers used for real-time qRT-PCR of shoot and berry gene expression. Primers were generated for real-time qRT-PCR of genes for comparison with microarray and EST frequency results. Gene name, gene model or contig identifier, forward primer (FP) and reverse primer (RP) sequences, and product size are shown. [file 1471-2229-11-86-S5.DOCX]

**Additional File 5 – List of primers used for real-time qRT-PCR of shoot and berry gene expression.**

Primers were generated for quantitative real-time RT-PCR of genes for comparison with microarray and EST frequency results. Name, gene or contig identifier, primer sequences and product size are shown.

|  | | | |
| --- | --- | --- | --- |
| **Gene Name** | **Gene Model** | **Primers (FP/RP)** | **Product size (bp)** |
| Unknown protein | GSVIVP00018707001 | (FP) 5’-GGAAGAATTCCTTACAGAGGAAGA-3' | 130 |
|  |  | (RP) 5’-GATTGGCTAGATCATTGATACAAA-3’ |  |
| Putative proline-rich cell wall protein | TC71834 | (FP) 5’-GCTCCACCACCCATCCAT-3' | 111 |
|  |  | (RP) 5’-GAACATGATGCACGAGATAGCA-3’ |  |
| Hypothetical protein | GSVIVP00028026001 | (FP) 5’-GCGACAGACGCTCTTGAAA-3' | 80 |
|  |  | (RP) 5’-ACAATCTTGAACGGCTGATACA-3’ |  |
| Hypothetical protein | GSVIVP00001371001 | (FP) 5’-AGCGCAGTACAACGGAGAGT-3' | 121 |
|  |  | (RP) 5’-CGCCCGTGAAGTAAATCATC-3’ |  |
| RD22-like | GSVIVP00032481001 | (FP) 5’-TGCTCAAAGTTAAGCCAGGA-3' | 116 |
|  |  | (RP) 5’-GCGCGGAACTAACAAATCTC-3’ |  |
| Hypothetical protein | GSVIVP00029417001 | (FP) 5’-CAGGCAGGTGGACAAGTTT-3' | 137 |
|  |  | (RP) 5’-AAAGGACAAGGAAAGTCCAGTT-3’ |  |
| ycf1 | GSVIVP00035570001 | (FP) 5’-ATGAATCGCTATTGGTTTGATAC-3' | 150 |
|  |  | (RP) 5’-GCATGTGTGCATTTGTTTACTT-3’ |  |
| Chloroplast latex aldolase | GSVIVP00035963001 | (FP) 5’-GCTCAGCTCGGGAAGTACAC-3' | 95 |
|  |  | (RP) 5’-TGCAAGTGCAGCAGCTTAGT-3’ |  |
| Glycine cleavage system H protein | GSVIVP00015781001 | (FP) 5’-CTTGATGGGTCCAAAGGAAT-3' | 111 |
|  |  | (RP) 5’-AGCTCAAGGTAAGTCGGTTCA-3’ |  |
| Nodulin 93 | GSVIVP00016856001 | (FP) 5’-TTCATAGTTGCGGACAAGACA-3' | 141 |
|  |  | (RP) 5’-ACATAACAGGTTTGCTCATGGA-3’ |  |
| Pectate lyase B | GSVIVP00020459001 | (FP) 5’-GAGCCAAGTCCTCTTCCAT-3' | 103 |
|  |  | (RP) 5’-CCCATTCAATTAACTGAGATTTAC-3’ |  |
| Similar to At4g36570 | TC45330 | (FP) 5’-TTCGGGACCTCAAGCATATC-3' | 109 |
|  |  | (RP) 5’-CCTTGGACTTTATTGCAGCTTT-3’ |  |
| Acyl-CoA synthetase | GSVIVP00009148001 | (FP) 5’-GATTCTCTCCCAAA-3' | 81 |
|  |  | (RP) 5’-AGCAGTAGAGATTTGGGCAAGG-3’ |  |
| Class II HSP | GSVIVP00032242001 | (FP) 5’-GCCCAAGATCATAGAGGTCAAG-3' | 102 |
|  |  | (RP) 5’-CCCAGACCAAATTCACAGAAA-3’ |  |
| Nthsp18p | GSVIVP00001312001 | (FP) 5’-AAGTTCCTTCGTCGGTTCAG-3' | 120 |
|  |  | (RP) 5’-CACCTCAGCCTTCTTCACCT-3’ |  |
| ATP synthase alpha chain | GSVIVP00035586001 | (FP) 5’-CGCTTTCTACTTCAGGAACAA-3' | 198 |
|  |  | (RP) 5’-TATCAGTAAAGAAATTTGGAATGATTT-3’ |  |
| Auxin-binding protein ABP19a | GSVIVP00016457001 | (FP) 5’-AACGATTTGCCAACTGAACTG-3' | 146 |
|  |  | (RP) 5’-AACAACAGATGAGTGATGATGGA-3’ |  |
| CCR protein | GSVIVP00037947001 | (FP) 5’-AGAGGCTTGCGATGATCTTG-3' | 93 |
|  |  | (RP) 5’-TTTCCCTGATGATGGAGACTT-3’ |  |
| Galactinol synthase | GSVIVP00019669001 | (FP) 5’-TTGGGAATTCGTGGAGTACAG-3' | 99 |
|  |  | (RP) 5’-GAAATGGCCGTCTTGTGAGT-3’ |  |
| Putative wound-induced protein | GSVIVP00029187001 | (FP) 5’-AGAGAGGAGAAGGCCAAGCA-3' | 140 |
|  |  | (RP) 5’-GGAATATGCCCTCCCTGGAT-3’ |  |
| eIF4a | GSVIVP00034135001 | (FP) 5’-TGCCATCAAATGTTGCTGAT-3' | 76 |
|  |  | (RP) 5’-TTGAAATAAGAAACCCCTTACCTC-3’ |  |
