# Supplementary material for: Identification of tissue-specific, abiotic stress-responsive gene expression patterns in wine grape (Vitis vinifera L.) based on curation and mining of large-scale EST data sets
Source: BMC Plant Biol. 2011 May 18;11:86. doi: 10.1186/1471-2229-11-86 (PMC3224124; doi:10.1186/1471-2229-11-86)
Supplement: Additional file 6 — List of primers used for real-time qRT-PCR of root gene expression. Primers were generated for real-time qRT-PCR corroboration of root-enriched gene expression estimated by EST frequency. Gene name, NCBI gene locus identifier, forward primer (FP) and reverse primer (RP) sequences and product size are shown. [file 1471-2229-11-86-S6.DOCX]

**Additional File 6 – List of primers used for real-time qRT-PCR of root gene expression.**

Primers were generated for quantitative real-time RT-PCR corroboration of root-enriched gene expression estimated by EST frequency. Name, gene identifier, primer sequences and product size are shown.

|  | | | |
| --- | --- | --- | --- |
| **Gene Name** | **NCBI Gene Symbol** | **Primers Used (FP/RP)** | **Product Size (bp)** |
| Resveratrol O-methyltransferase | LOC100259140 | (FP) 5’-TGAGCTCCCAGTCAACCCAGAGA-3’ | 51 |
|  |  | (RP) 5’-CGCATGAGACGGTACACGCATT-3’ |  |
| NGATHA1 transcription factor | LOC100259130 | (FP) 5’-TGAGCGCGGTGTTGGAGAATCA-3’ | 59 |
|  |  | (RP) 5’-GCCGGCGCCTCCAGTCTAT-3’ |  |
| Cinnamyl-alcohol dehydrogenase | LOC100261642 | (FP) 5’-GGTGGCCAATGAGCGCTATGTGATT-3’ | 68 |
|  |  | (RP) 5’-GGAGCGGTGCTCCCTTGTCA-3’ |  |
| AP2/ERF transcription factor | LOC100250476 | (FP) 5’-AGCAGCAATGGACGCAGCATGA-3’ | 130 |
|  |  | (RP) 5’-ATCAGCCGACTGCACCATACGA-3’ |  |
| (E, E) –alpha-Farnesene Synthase | LOC100260714 | (FP) 5’-AAGGGTGCACGTTGCTTCTAGTGT-3’ | 80 |
|  |  | (RP) 5’-TCAACTCATCATACGCCTCCTGCTT-3’ |  |
| Myb transcription factor-like a | LOC100265807 | (FP) 5’-GCCATTGGCCACAGATGGAGGAT-3’ | 54 |
|  |  | (RP) 5’-GCGGAGGCCGGCGAATCA-3’ |  |
| Aquaporin TIP1;4 | LOC100250080 | (FP) 5’-GCAACCGGCGGATTGACAACA-3’ | 54 |
|  |  | (RP) 5’-TGCGCTCACGCCAGAGGATAG-3’ |  |
| Flavonol 3-O-glucosyltransferase | LOC100251889 | (FP) 5’-GATGCAGTCCACCAGCTCATCCT-3’ | 137 |
|  |  | (RP) 5’-GCCGCACTGGACGTGGAGAA-3’ |  |
| Myb transcription factor-like b | LOC100243049 | (FP) 5’-CAACAGCAGCCACCGCAACA-3’ | 52 |
|  |  | (RP) 5’-GTGACCAGCACCGCCTCTGT-3’ |  |
| Nitrate reductase 2 (NR2) | LOC100264320 | (FP) 5’-GTCCACGGCCACGTCTACGA-3’ | 93 |
|  |  | (RP) 5’-AGTGCAGTCGGTGCCAGCAT-3’ |  |
| Actin 7 (control gene) | LOC100232968 | (FP) 5’-CTTGCATCCCTCAGCACCTT-3’ | 82 |
|  |  | (RP) 5’-TCCTGTGGACAATGGATGGA-3’ |  |
